# Disease-specific prioritization of non-coding GWAS variants based on chromatin accessibility

**DOI:** 10.1016/j.xhgg.2024.100310

**Published:** 2024-05-21

**Authors:** Qianqian Liang, Abin Abraham, John A. Capra, Dennis Kostka

**Affiliations:** 1 Department of Computational & Systems Biology and Center for Evolutionary Biology and Medicine, University of Pittsburgh School of Medicine, Pittsburgh, PA, USA; 2 Department of Human Genetics, University of Pittsburgh School of Public Health, Pittsburgh, PA, USA; 3 Children’s Hospital of Philadelphia, Philadelphia, PA, USA; 4 Department of Epidemiology & Biostatistics and Bakar Computational Health Sciences Institute, University of California, San Francisco, San Francisco, CA, USA

## Abstract

Non-protein-coding genetic variants are a major driver of the genetic risk for human disease; however, identifying which non-coding variants contribute to diseases and their mechanisms remains challenging. *In silico* variant prioritization methods quantify a variant’s severity, but for most methods, the specific phenotype and disease context of the prediction remain poorly defined. For example, many commonly used methods provide a single, organism-wide score for each variant, while other methods summarize a variant’s impact in certain tissues and/or cell types. Here, we propose a complementary disease-specific variant prioritization scheme, which is motivated by the observation that variants contributing to disease often operate through specific biological mechanisms. We combine tissue/cell-type-specific variant scores (e.g., GenoSkyline, FitCons2, DNA accessibility) into disease-specific scores with a logistic regression approach and apply it to ∼25,000 non-coding variants spanning 111 diseases. We show that this disease-specific aggregation significantly improves the association of common non-coding genetic variants with disease (average precision: 0.151, baseline = 0.09), compared with organism-wide scores (GenoCanyon, LINSIGHT, GWAVA, Eigen, CADD; average precision: 0.129, baseline = 0.09). Further on, disease similarities based on data-driven aggregation weights highlight meaningful disease groups, and it provides information about tissues and cell types that drive these similarities. We also show that so-learned similarities are complementary to genetic similarities as quantified by genetic correlation. Overall, our approach demonstrates the strengths of disease-specific variant prioritization, leads to improvement in non-coding variant prioritization, and enables interpretable models that link variants to disease via specific tissues and/or cell types.

## Teaser

Non-coding genetic variants constitute the majority of disease-associated genetic variation in humans. In this study, Liang et al. show that variant prioritization within a specific disease context improves performance and that it enables the linking of variants to disease via specific tissues and cell types.

## Introduction

Characterizing non-coding genetic variants in the human genome is essential for making progress toward better understanding the genetic components of disease, as ∼90% of disease-associated variants discovered by genome-wide association studies (GWASs) are located in non-protein-coding regions.^1^ Furthermore, whole-genome sequencing discovers disease-associated variants genome-wide^2^^,^^3^ and is increasingly becoming an assay of choice. Therefore, approaches for characterizing and prioritizing non-coding variants can be expected to play an increasingly important role, especially when assessing discovered variants in the context of functional follow-up experimental studies.

Efforts to computationally characterize and better understand non-coding variants take advantage of sequence, functional genomics, comparative genomics, and epigenomics data,^4^^,^^5^^,^^6^ and more. These data are combined and used to train and develop supervised and/or unsupervised models that attempt to quantify a variant’s impact.^7^ We find it conceptually useful to distinguish between variant scores that model overall impact (that is on the level of the whole organism, organism-level scores) and scores that quantify impact in a specific context, like a tissue or a cell type (i.e., tissue-level scores). Examples of methods for obtaining organism-level scores are CADD,^8^ Eigen,^9^ and LINSIGHT,^10^ while scores from methods like GenoSkyline,^11^ Fitcons2,^12^ and FUN-LDA^13^ are tissue specific. In addition, GenoNet^14^ is an approach that leverage existing disease-specific scores together with genome-wide functional annotation to better predict organism-level and tissue-specific variant impact. RegBase,^15^ on the other hand, combines existing scores for broad categories like pathogenic, cancer-driver, or regulatory variants. Often, interest in a set of variants is from the perspective of studying a specific disease. In that case, organism-level scores are likely to be overly general. That is, a variant’s impact might be considered high because it disrupts the functional role of a sequence element. However, that functional role may be unrelated to the disease of interest. In one study, for instance, organism-level scores like CADD and DANN were unable to discover an enrichment signal for brain-related traits, while context-specific variant scores focusing on relevant tissues were successful.^16^ This demonstrates that tissue-specific scores can address the issue of disease specificity to some extent. Therefore, approaches like TSEA-db^17^ and efforts like EpiMap^18^ aim to identify disease-relevant tissues. Despite these approaches and their successes, it remains the case that aspects of disease-relevant tissues typically remain unknown, and often, more than one tissue is implicated with a specific trait (termed “multifactorial” and “polyfactorial” traits).^18^ This suggests the use of disease-specific variant scores that characterize variants in the context of a specific disease phenotype of interest.

Computational methods for disease-specific variant prioritization do exist. Some approaches are geared toward one disease (e.g., congenital heart disease,^19^ amyotrophic lateral sclerosis^20^) or toward a specific class of diseases (e.g., autoimmune diseases^21^). This focus prevents them from being readily adapted to other disease types. Others, like DIVAN,^22^ PINES,^23^ and ARVIN,^24^ cover a broader range of disease types. Of these, ARVIN requires *a priori* knowledge of disease-relevant tissues, whereas DIVAN and PINES do not. PINES uses an enrichment-based method to predict and up-weight disease-relevant tissues/cell types, whereas DIVAN uses a more complex machine learning algorithm. The PINES approach has been evaluated on a relatively small set of traits, while DIVAN’s more complex model renders understanding the relationship between different tissues and diseases difficult.

**Figure 1:**
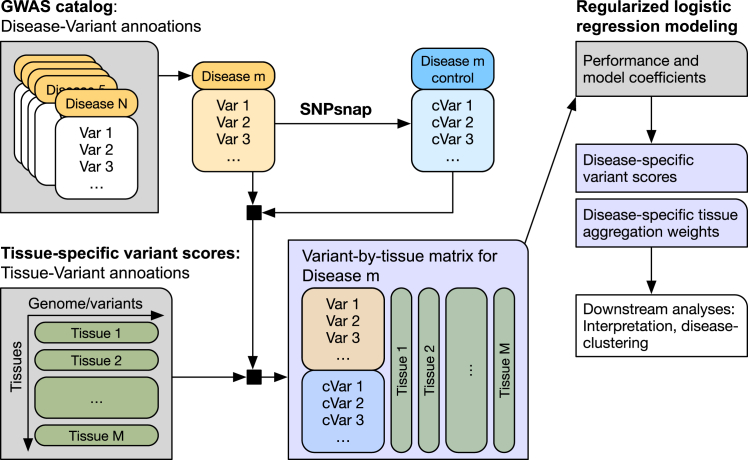
Summary of our approach Variant annotations from the GWAS Catalog were augmented with disease-specific control variants using SNPsnap. For each disease, a logistic regression model relates disease-variant association to tissue-specific variant scores. Results are disease-specific variant scores and disease-specific tissue aggregation weights that can serve as input for further analyses, like the calculation of disease-disease similarities.

In this work, we derive disease-specific variant scores by combining published tissue-specific scores (Figure 1). We use a carefully regularized logistic regression approach to derive data-driven disease-specific combination weights, which allow us to better associate variants with disease. In addition, they enable us to quantify a similarity between different disease phenotypes. Using the NHGRI-EBI GWAS Catalog,^1^ we compiled a benchmark dataset containing about 25,000 phenotype-associated non-protein-coding single-nucleotide variants (SNVs) across 111 disease phenotypes (together with matched random controls). We then demonstrate that using disease-specific combination weights outperforms conventional organism-level approaches, that our interpretable model has competitive performance, and that it enables a disease similarity measure that captures information complementary to established measures like genetic correlation.

## Results

### Non-coding GWAS variants associated with disease phenotypes and matched controls

In order to study variant prioritization methods, we created a dataset of “positive” (i.e., disease-associated) non-coding variants, matched with a random set of “negative” or “control” variants. This setup allowed us to quantitatively assess prioritization methods based on their performance in discriminating positive from control variants.

#### Disease-associated non-coding SNVs

**Figure 2:**
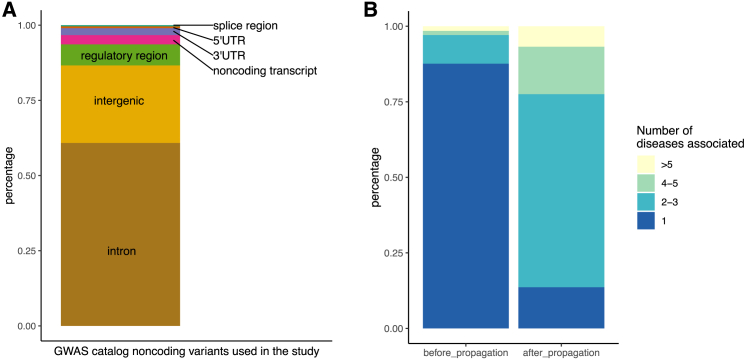
Disease-associated non-coding SNVs (A) Genomic context of non-coding SNVs used in this study. (B) Percentage of the SNVs used that are annotated to 1, 2–3, 4–5, or more than 5 disease phenotypes before and after propagating SNV-phenotype associations according to EFO parent-child annotations. Genomic context annotation is adapted from the CONTEXT column from the GWAS Catalog, where we combine splice donor, splice region, and splice acceptor variants into splice variants and combine transcription factor binding variants and regulatory regions variants into regulatory region variants.

We used a subset of SNVs reported in the EBI/NIH GWAS Catalog^1^ to compile an inventory of disease-associated non-coding variants. Specifically, we focused on reported variants that (1) do not overlap protein-coding sequence (see materials and methods) and (2) are associated with a disease phenotype as noted in the Experimental Factor Ontology (EFO) trait description, which is provided within the catalog. We define disease phenotypes as descendants of the EFO term “disease” (EFO: 0000408). Focusing on disease terms with at least 100 annotated SNVs resulted in 26,080 associations involving 20,656 SNVs and 67 disease phenotypes. The EFO provides parent-child relations between disease terms (parent = more general, child = more specific), and propagating SNVs from child terms to parent terms increased the number of disease phenotypes with at least 100 SNVs, resulting in 77,028 associations between 25,516 SNVs and 111 diseases. We find that most of the SNVs we recover are located in intronic (60.5%) and intergenic (25.8%) sequences (Figure 2A) and that a majority of SNVs are directly annotated to a single disease phenotype (Figure 2B). After propagating annotated SNVs from child to parent terms, SNV-to-disease annotations become predominantly many:many (Figure 2B). Data SD1 lists disease terms and corresponding numbers of disease-associated SNVs.

#### Control SNVs

For each disease-associated SNV, we selected ∼10 matched control SNVs using a re-implementation of the SNPsnap approach^25^ while avoiding duplicate control SNVs across the overall dataset (see materials and methods). This yielded 255,137 control SNVs (for some disease-associated SNVs, we could not retrieve the full ten control SNVs). With these results, we have access to data for 111 disease terms, containing disease-associated SNVs together with matched controls. Data SD2 and SD3 contain information about all disease and control SNVs used in this study, respectively.

### Disease-specific scores improve non-coding variant prioritization

Before we describe our approach for disease-specific variant scoring, we evaluated the performance of existing organism-level scores on our dataset in disease-specific non-coding variant prioritization with organism-level variant scores is only moderately successful. We then detail our approach in disease-specific aggregation weights for tissue-specific variant scores and DHS scoring outperforms other scores, before we show that it outperforms current organism-level scores in DHS tissue-weighted scoring outperforms organism-level variant scores. Our overall approach is summarized in Figure 1.

#### Disease-specific non-coding variant prioritization with organism-level variant scores is only moderately successful

**Figure 3:**
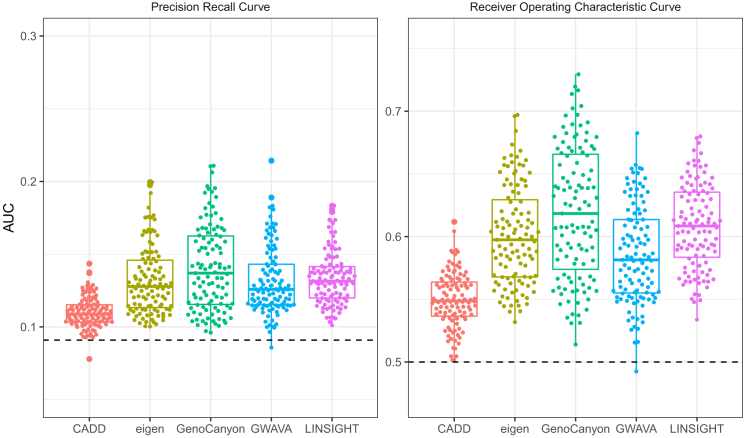
Organism-level variant scores are moderately successful in prioritizing noncoding disease-associated variants Different organism-level variant prioritization scores are shown on the x axis, and the y axis displays performance in terms of average precision (area under the precision recall curve, left) and area under the receiver-operator curve (right). Each point represents a specific disease term from the experimental factor ontology. Horizontal lines spanning datasets show expectations under random guessing.

We assessed how well current commonly used organism-level variant scores are able to prioritize disease-associated vs. control SNVs for the 111 disease terms we studied. Figure 3 summarizes the results, where boxplots of two performance measures (area under the receiver operator characteristic curve [AUROC] and average precision [= area under the precision-recall curve]) are shown for CADD,^8^ Eigen,^9^ GenoCanyon,^26^ GWAVA,^27^ and LINSIGHT^10^ scores. We find that organism-level scores, while improving upon random guessing, are only moderately successful in correctly prioritizing disease-associated non-coding variants. Comparing variant scores with one another, we find that relative performance differences appear overall robust with respect to the metric employed (AUROC vs. average precision). It is qualitatively visible that CADD performs less favorably than other methods but also that there are differences between them. We therefore compared performance between different scores in more detail.

We studied the performance of different scores at two levels of resolution: in aggregate across all disease terms and for each disease term separately. For both approaches, we used Wilcoxon signed-rank tests to decide whether one score significantly outperforms another score (= significant *p* value) or whether performance is tied (=non-significant *p* value); see the materials and methods section. The results are summarized in Table 1. We find that GenoCanyon has better performance compared with other variant scores, followed by LINSIGHT, GWAVA, and Eigen, while CADD is consistently outperformed by the other methods. Performance differences between LINSIGHT, GWAVA, and Eigen are not significant when aggregating across disease terms (last three columns in Table 1); however, when counting individual terms, LINSIGHT has the most wins and fewest losses, while Eigen has the most losses and fewest wins, leading to the ordering displayed in Table 1. Data SD4 and SD5 contain results for all comparisons. Overall, these quantitative results are in line with the visual impression from Figure 3. Next, we investigated if the performance of organism-level variant scores could be improved by using tissue-specific scoring approaches.

**Table 1:** Relative performance of organism-level variant scores

Score/method	By disease term			Aggregated		
	Wins	Losses	Ties	Wins	Losses	Ties
GenoCanyon	307	106	31	4	0	0
LINSIGHT	281	146	17	1	1	2
GWAVA	221	196	27	1	1	2
eigen	219	201	24	1	1	2
CADD	24	403	17	0	4	0

Wins, losses, and ties refer to significantly better (or worse, or tied) performance across all possible pairings (see materials and methods). Columns 2-4 summarize separate comparisons for each disease term (for each row, there are four other methods and 111 terms, i.e., 444 comparisons), while the last three columns represent the results of comparisons between scores aggregated across terms. Average precision was used as the performance metric, and Wilcoxon signed-rank tests were used to determine wins and losses (*p* values equal or larger than 0.05 are reported as ties).

#### Disease-specific aggregation weights for tissue-specific variant scores

We studied three tissue-specific scores for variant prioritization to explore if their usage can improve the performance of organism-level scores. Specifically, we used Genoskyline^11^ and Fitcons2^12^ as scores designed to prioritize variants, and we also evaluated DNase I hypersensitivity (DHS) profiles from the ENCODE project.^6^ All of these scores are available for contexts^5^ spanning a diverse set of cell and tissue types, including heart, brain, immune cells, and more.

**Figure 4:**
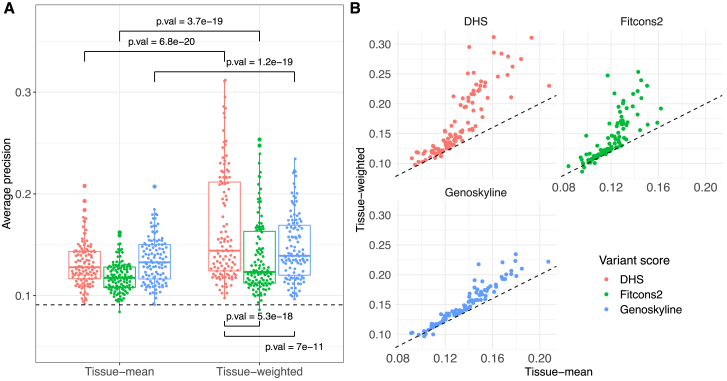
Disease-specific tissue weights improve variant prioritization Performance of three tissue-specific variant scores (DHS, Fitcons2, GenoSkyline) is used to prioritize non-coding disease-associated variants for disease terms using two approaches: tissue-mean (i.e., disease agnostic, baseline) on the left side and tissue-weighted (i.e., disease specific) on the right side. *p* values were calculated using a Wilcoxon signed-rank test (A). Scatterplot of tissue-mean vs. tissue-weighted performance (average precision) for each tissue-specific score; dashed line denotes the diagonal (B).

For each tissue-specific score, we assess two approaches to prioritize variants. First, as a baseline approach, we aggregate scores across tissues in a disease-agnostic way. That is, for a specific variant, we average scores at the variant position across all tissues (termed tissue-mean), essentially producing an organism-level type score, independent of the disease term under consideration. Second, we aggregate scores across tissues in a disease-specific way. Briefly, we train a regularized logistic regression model for each disease term that learns disease-specific tissue aggregation weights. In a nested cross-validation setup, learned weights are then applied to held-out variants, allowing for a fair performance assessment of this approach (termed tissue-weighted); see the materials and methods section. Figure 4 summarizes our findings.

In Figure 3A, we show the tissue-mean performance (measured by average precision) for the three scores we study on the left and tissue-weighted performance on the right. For all three scores, tissue-weighted significantly outperforms tissue-mean (Wilcoxon signed-ranks test, *p* < 0.0001). Figure 4B shows tissue-mean vs. tissue-weighted comparisons for each score, and we see that in almost all disease terms, tissue-weighted outperforms tissue-mean. See Data SD6 and SD7 for tissue-mean vs. tissue-weighted performances for each disease term and for aggregated performances across all disease terms. The improvement remains evident if we limit disease-associated SNVs to one variant per linkage disequilibrium (LD) block and also when we ensure that the SNVs in the training and test datasets are not on the same chromosome (see Figures S17–S20 and the supplemental information for more details).

**Figure 5:**
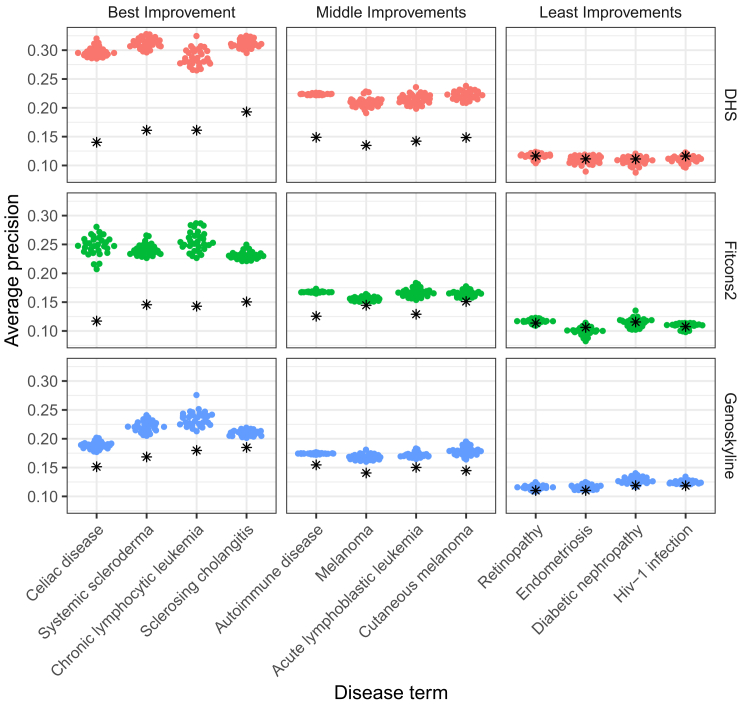
Improvement through disease-specific tissue weights is consistent across scores but varies with disease term Shown is the performance of tissue-weighted variant scores (colored points) vs. tissue-mean (black asterisks) as a baseline, for three tissue scores (rows) and four diseases, stratified by improvement observed: best improvement for the first column, moderate improvement for the middle column, and least improvement for the right column. The x axes denote disease terms and the y axis average precision. Different points for tissue-weighted scores represent different data splits in the nested cross-validation procedure.

While the performance gain for tissue-weighted is broadly consistent across diseases, for some it is more pronounced than for others. To illustrate this observation, we selected four disease terms with a high performance gain, four terms with a medium gain, and four terms where we observed the least gain (best improvement, ranking 1–4; middle improvement, ranking 20–23; least improvement, ranking 108–111). Figure 5 shows our findings, where variability in tissue-weighted performance induced by varying train-test-fold splits during cross-validation is also displayed. We see that for celiac disease (EFO: 0001060), systemic scleroderma (EFO: 0000717), chronic lymphocytic leukemia (EFO: 0000095), and sclerosing cholangitis (EFO: 0004268), performance is consistently improved for all three tissue-weighted scores, while for retinopathy (EFO: 0003839), endometriosis (EFO: 0001065), diabetic nephropathy (EFO: 0000401), and HIV-1 infection (EFO: 0000180), we find no improvement. We also note that disease terms with a pronounced improvement appear to have a better baseline (i.e., tissue-mean) performance than disease terms where we find little or no benefit of the tissue-weighted approach. The improvement for diseases shown in Figure 5 is largest for DHS, but, consistent with Figure 4, we see improvement for Fitcons2 and GenoSkyline as well.

#### DHS scoring outperforms other scores

Having used three tissue-specific scores to derive disease-specific variant scores, we assessed their relative performance in this context. To quantify relative performance, we proceed similarly to organism-level scores. Focusing on pairwise comparisons, we find that disease-specific scores derived from DHS scores outperform GenoSkyline and Fitcons2 for most disease terms and on average (see Table 2). This can also be observed in Figures 4 and 5, which often show higher average precision values for DHS than for the other two scores. Notably, the baseline (i.e., tissue-mean) performance of DHS does not appear significantly better than that of Genoskyline (Figure 4). Data SD8 and SD9 contain details for comparisons between DHS, Fitcons2, and GenoSkyline for all disease terms. Next, we explored whether disease-specific tissue weights outperform organism-level scores.

**Table 2:** DHS outperforms other tissue-specific scores

Score/method	By disease term			Aggregated		
	Wins	Losses	Ties	Wins	Losses	Ties
DHS	180	22	20	2	0	0
Genoskyline	96	94	32	1	1	0
Fitcons2	19	179	24	0	2	0

Wins, losses, and ties refer to significantly better (or worse, or tied) performance across all possible score pairings (see materials and methods). Columns 2-4 summarize separate comparisons for each disease term (for each row, there are two other methods and 111 terms, i.e., 222 comparisons), while the last three columns represent the results of comparisons aggregated over disease terms. Average precision was used as the performance metric, and the Wilcoxon signed-rank test was used to determine wins and losses (*p* values equal or larger than 0.05 are reported as ties).

#### DHS tissue-weighted scoring outperforms organism-level variant scores

To compare the DHS tissue-weighted score with organism-level scores, we directly contrasted their performance. Similar to before, Table 3 summarizes DHS “wins” (= significantly better performance of DHS tissue-weighted, *p* ≤ 0.05), losses, and ties, compared with five organism-level variant scores, individually (i.e., per disease term) and aggregated across disease terms. In addition, Table S4 summarizes pairwise comparisons between tissue-weighted DHS and each organism-level score. We find that DHS tissue-weighted outperforms all organism-level scores in the aggregated analyses and that it outperforms all other scores on the majority of disease terms (it only performs significantly worse than any other score in 44 out of 550 comparisons).

**Table 3:** DHS outperforms organism-level variant scores

Score/method	By disease term			Aggregated		
	Wins	Losses	Ties	Wins	Losses	Ties
DHS	474	44	37	5	0	0
GenoCanyon	314	198	43	4	1	0
LINSIGHT	298	230	27	1	2	2
GWAVA	233	289	33	1	2	2
eigen	223	299	33	1	2	2
CADD	28	510	17	0	5	0

Wins, losses, and ties refer to significantly better (or worse, or tied) performance across all possible score pairings (see materials and methods). Columns 2-4 summarize separate comparisons for each disease term (for each row, there are two other methods and 111 terms, i.e., 555 comparisons), while the last three columns represent the results of comparisons aggregated over terms. Average precision was used as the performance metric, and the Wilcoxon signed-rank test was used to determine wins and losses (*p* values equal or larger than 0.05 were reported as ties).

GenoCanyon is the most competitive organism-level score, where DHS is significantly better for 92 terms out of 111 (∼83%). Interestingly, LINSIGHT performs better against DHS than GenoCanyon, which is the best overall performing organism-level score (see Table S4). Data SD10 contains detailed results for each comparison. We also find that DHS outperforms organism-level scores when aggregating over disease terms (also see Data SD11).

**Figure 6:**
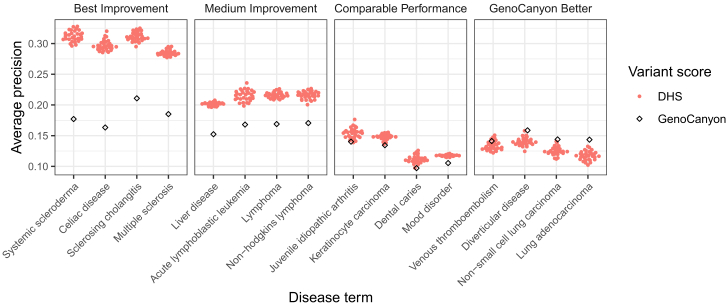
DHS disease-specific tissue weights improve variant prioritization compared with organism-level scores For four strata (best improvement, middle improvement, comparable performance, and worse performance) we selected four disease terms and compared performance results. GenoCanyon (best organism-level score) performance is denoted in black and DHS tissue-weighted in red. Different performances of DHS tissue-weighted represent variation different data splits during nested cross-validation (see materials and methods).

To illustrate the gain in performance, we selected four example disease terms where disease-specific variant prioritization yielded high improvements, medium improvements, comparable performance, and worse performance, respectively. Selection was based on ranking differences between DHS and GenoCanyon: best improvement, ranks 1–4; medium improvements, ranks 25–28; comparable performance, ranks 64–67; and GenoCanyon better, ranks 108–111. The results are summarized in Figure 6, where we find substantial improvements using tissue-weighted scoring for systemic scleroderma (EFO: 0000717), celiac disease (EFO: 0001060), sclerosing cholangitis (EFO: 0004268), and multiple sclerosis (EFO: 0003885), for which we have already noticed substantial improvement of DHS tissue-weighted over DHS tissue-mean. Disease terms where GenoCanyon performs better include venous thromboembolism (EFO: 0004286), diverticular disease (EFO: 0009959), non-small cell lung carcinoma (EFO: 0003060), and lung adenocarcinoma (EFO: 0000571).

To make DHS tissue-weighted scores available, we generated pre-computed scores for 111 diseases at every base across the genome (for chromosomes 1–22, available at https://doi.org/10.7910/DVN/AUAJ7K). Scores were calculated at 25 bp resolution using the hg19 assembly, in the same resolution and on the same assembly as the DHS scores.

### DHS scoring performs well compared with DIVAN

Here, we compare the performance of tissue-weighted DHS scoring with DIVAN,^22^ a disease-specific variant score for 45 diseases. DIVAN is based on a more complicated feature selection and ensemble learning framework, and it uses a variety of other functional genomics features in addition to DHS. To compare our method with DIVAN, we mapped EFO disease terms to MeSHs and use MeSH terms in this section (see Data SD12). Because DIVAN is used as a supervised learning approach, and because the published model was trained using GWAS SNVs, it was necessary to create specific train and test datasets to ensure a meaningful comparison between tissue-weighted DHS and DIVAN.

Therefore, to assess the performance of both DIVAN and DHS, we created a test set of disease-associated variants (and their matched controls) that were published later than 2016 (DIVAN’s publication date). That is, these variants are unlikely to have been a part of DIVAN’s training data. We also created a training set for DHS tissue-weighted containing only SNVs published prior to 2016. This resulted in training data that (1) are distinct from the test set and (2) draw on similar information that was available for DIVAN’s training. Further on, we only selected disease terms for this training/test data combination where at least 20 term-associated SNVs were present in the training data and at least 50 SNVs were present in the test data. This approach yielded 29 disease terms for this analysis. We then re-trained tissue-weighted DHS on this training data and compared it with DIVAN on the test data. In addition, we added the organism-level GenoCanyon score as a reference.

**Figure 7:**
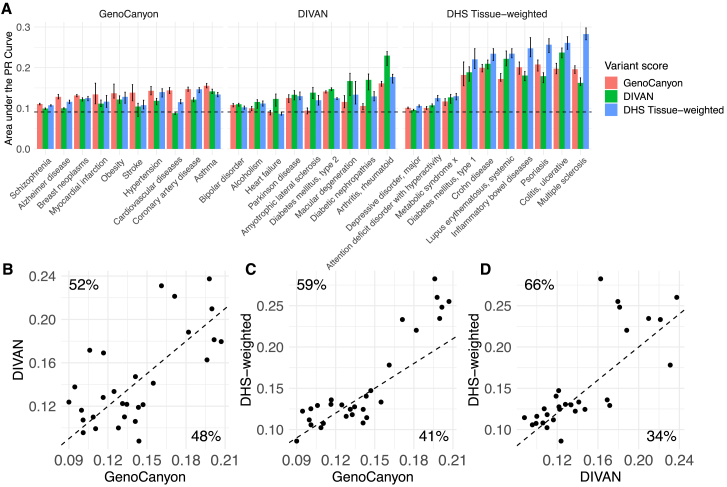
DHS tissue-weighted scoring outperforms DIVAN Performance of DIVAN, GenoCanyon, and DHS tissue-weighted across a test set, with disease terms grouped by the best-performing method. Vertical stripe indicates the minimum and maximum performance of 30 bootstrap samples (A). Performance scatterplots of GenoCanyon vs. DIVAN performance (B), GenoCanyon vs. DHS-weighted (C), and DIVAN vs. DHS-weighted performance (D). Average precision was used for these plots; dashed lines denote equal performance. Percentages denote the fraction of points above and below the diagonal, respectively.

To assess performance, we performed all pairwise comparisons for each disease term and evaluated performance based on average precision. Table 4 summarizes observations, where we find that DHS performs significantly better than GenoCanyon and DIVAN in a majority of comparisons; however, there is a substantial number of comparisons (22 out of 58) where either GenoCanyon or DIVAN outperforms DHS. Figure 7 further illustrates these comparisons. In Figure 7A, we show the performance across disease terms, grouped by the best-performing method. We see that tissue-weighted DHS outperforms DIVAN and GenoCanyon substantially on multiple sclerosis (MeSH: D009103), psoriasis (MeSH: D011565), and inflammatory bowel disease (MeSH: D015212); DIVAN outperforms GenoCanyon and DHS on arthritis, rheumatoid (MeSH: D001172) and heart failure (MeSH: D006333); and GenoCanyon outperforms DHS and DIVAN on stroke (MeSH: D020521) and Alzheimer disease (MeSH: D000544). In Figures 7B–7D, we directly summarize comparison results; we observe that the DHS tissue-weighted score often has an advantage in terms where prioritization efforts are overall more successful (top right quadrants). Finding overall good performance for our approach, we next more closely examined the disease-specific tissue aggregation weights we derive with our approach.

**Table 4:** DHS tissue-weighted disease-specific scoring outperforms DIVAN

Score	Wins	Losses	Ties	Winning percentage (%)
DHS	34	22	2	61
GenoCanyon	26	31	1	46
DIVAN	25	32	1	44

Across 29 disease terms, this table summarizes all pairwise comparison for DHS tissue-weighted, GenoCanyon, and DIVAN using a specifically created test dataset. Wins, losses, and ties refer to significantly better (or worse, or tied) performance. Average precision was used as the performance metric, and the Wilcoxon signed-rank test was used to determine wins and losses (*p* values equal or larger than 0.05 were ties). Winning percentage = \#wins/(\#wins+\#losses).

### Disease-specific tissue weights reflect biomedical relevance

In addition to prioritizing SNPs, we can interpret the disease-specific tissue weights that our model learns in the context of disease mechanisms. Specifically, large tissue weights implicate tissues with a prominent role in associating SNVs with a disease in our model; therefore, one may hypothesize that such tissues or cell types have a function in the etiology of that disease. To investigate this hypothesis, we analyzed tissue weights of the top-performing models we derived, where each model represents a different disease.

The results are summarized in Table 5; they include the two top-performing models, systemic scleroderma (rank 1) and sclerosing cholangitis (rank 2). In order to report a diverse range of diseases, we next excluded any diseases that are descendants of immune system disease (EFO: 0000540) or lymphoma (EFO: 0000574). From the remaining diseases, we identify the next three highest-ranked models: colorectal adenoma (rank 15), atrial fibrillation (rank 20), and cutaneous melanoma (rank 21). For each disease, we list the five tissues with the largest tissue weights and their tissue group.

**Table 5:** Top-ranked tissues for five diseases

Rank	ID	Tissue name	Group
**Systemic scleroderma**			

1	E116	GM12878 lymphoblastoid cells	blood
2	E032	primary B cells from peripheral blood	blood
3	E041	primary T helper cells PMA-I stimulated	blood
4	E123	K562 leukemia cells	blood
5	E030	primary neutrophils from peripheral blood	blood

**Sclerosing cholangitis**			

1	E116	GM12878 lymphoblastoid cells	blood
2	E061	foreskin melanocyte primary cells skin03	skin
3	E102	rectal mucosa donor 31	gi_rectum
4	E041	primary T helper cells PMA-I stimulated	blood
5	E029	primary monocytes from peripheral blood	blood

**Colorectal adenoma**			

1	E102	rectal mucosa donor 31	gi_rectum
2	E110	stomach mucosa	gi_stomach
3	E057	foreskin keratinocyte primary cells skin02	skin
4	E101	rectal mucosa donor 29	gi_rectum
5	E028	breast variant human mammary epithelial cells (vHMECs)	breast

**Atrial fibrillation**			

1	E083	fetal heart	heart
2	E108	skeletal muscle female	muscle
3	E107	skeletal muscle male	muscle
4	E088	fetal lung	lung
5	E120	HSMM skeletal muscle myoblast cells	muscle

**Cutaneous melanoma**			

1	E061	foreskin melanocyte primary cells skin03	skin
2	E059	foreskin melanocyte primary cells skin01	skin
3	E117	HeLa-S3 cervical carcinoma cell line	cervix
4	E041	primary T helper cells PMA-I stimulated	blood
5	E122	HUVEC umbilical vein endothelial primary cells	vascular

This shows the top-five tissues with the largest tissue weights in the corresponding model we derive, for five diseases. The first column is the tissue rank, the second the tissue’s roadmap ID, the third the tissue name, the fourth the tissue group.

The tissues we associate with disease, overall, appear reasonable and generally are in line with existing knowledge about disease mechanisms. Systemic scleroderma is an autoimmune disorder that can affect skin and internal organs. We find that GM12878 lymphoblastoid cells (a type of B cell) are among the highest-weighted tissues, as were other types of B cells (primary B cells and B cell lymphoma, respectively). This is in line with previous studies that have shown that B cells play a role in system scleroderma.^28^^,^^29^ Sclerosing cholangitis is an inflammatory condition that leads to scarring and narrowing of the bile ducts.^30^ We highlight various inflammation-related types of blood cells, such as T cells and monocytes, which were previously suggested to play a role in the disease.^31^ Colorectal adenoma is a benign tumor that develops in the lining of the colon or rectum. Our model identified rectal mucosa and stomach mucosa as the most-highly weighted tissues, and the function of rectal mucosa in colorectal cancer has been previously studied.^32^ While the direct relationship between other gastrointestinal tissues and the development of colorectal adenoma has not been established, the association between gastrointestinal microbiome and colorectal adenomas has been discovered.^33^ Regarding atrial fibrillation, our approach highlights fetal heart and lung tissues. In addition, we identified skeletal muscle cells. In the case of cutaneous melanoma, a type of skin cancer, our approach emphasizes foreskin melanocyte cells and a specific type of T cell. Apart from these, we highlight cervical carcinoma cell lines and endothelial primary cells.

Overall, we conclude that the tissue weights we derive carry biomedically meaningful information and are able to highlight tissue contexts that may play a role in disease etiology. To further explore this finding, we used a resource of the epimap consortium,^18^ where disease-tissue associations are reported that derived differently from the ones we obtained in two key ways: first, epimap uses their enhancer definitions based on a much larger set of genome annotations. Second, epimap’s enrichment test contrasts disease-associated SNP enrichment in a specific tissue’s enhancer set compared to all enhancers, whereas our method effectively compares open chromatin harboring disease-associated SNPs vs. control SNPs tissue by tissue. Nevertheless, the results are summarized in Table S7, and we find that out of the 25 tissues we associate with disease terms, 14 have an estimated false discovery rate of less than 4% in the epimap analysis as well. Notably, a ground truth for these association is generally unknown, but we interpret the overlap in associations as encouraging, while complementary associations are expected, given the differences in methodology. Based on this overall finding of meaningful disease-tissue associations, we next further explored the use of tissue weights in disease characterization.

### Disease-term similarity based on DHS tissue-weighted modeling reveals meaningful groups

Disease-specific tissue weights for aggregating DHS scores, which are learned by our approach, can highlight tissues and cell types with a role in the disease (see previous section). Therefore, we derived and explored a measure for disease similarity based on these weights.

#### Disease similarities based on disease-specific tissue weights for non-coding variant prioritization

In our DHS tissue-weighted approach, for each disease term, DNA accessibility across the same set of tissue and cell-type contexts is used to predict whether a certain SNV is disease associated or not. This results in disease-specific tissue aggregation weights (that is, coefficients in our logistic regression model) ${\left\{\beta^{\left(i\right)}\in R^{d}\right\}}_{i=1}^{n}$, where *i* is indexing disease terms, *n* is the number of disease terms studied, and *d* denotes the number of tissues/cell types with DHS scores. For our similarity measure between two diseases, say *i* and *j*, we then use a version of the Pearson correlation between $\beta^{\left(i\right)}$ and $\beta^{\left(j\right)}$ that takes uncertainty in the estimated aggregation weights into account (see materials and methods). That is, if an overlapping set of tissues/cell types drive the prioritization of SNVs for two diseases, then the similarity is high; if different tissues are used, then the similarity is low.

**Figure 8:**
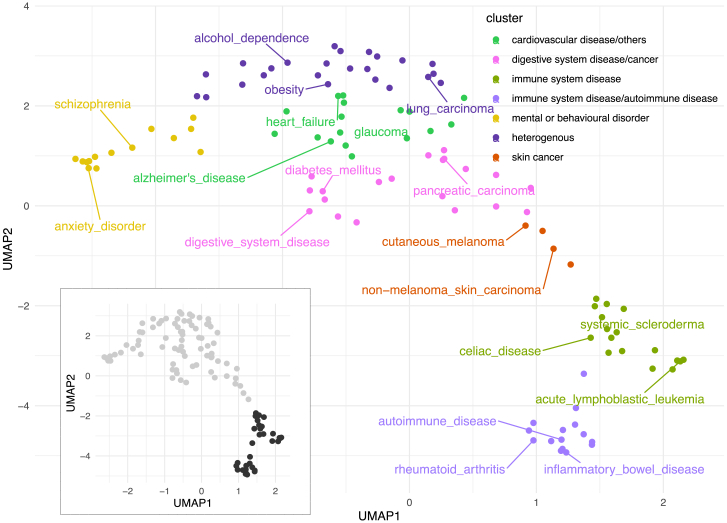
Similarity-based two-dimensional projection visualizes 111 diseases Two dominant disease groups emerge in this visualization (immune system-related disease terms [black] and others [gray], in the inlay). Hierarchical clustering was used to group diseases into seven clusters, with colors indicating broad disease types (see Table 6 for details).

Using this approach, we calculated disease similarities for the 111 disease terms we study. Resulting similarities are visualized in Figure 8, where we show a similarity-based two-dimensional uniform manifold approximation and projection (UMAP) of disease terms. We observe that disease terms segregate into separate groups, with a coarse grouping between immune-related diseases (bottom left inlay, black) and others (bottom left inlay, gray). A higher-resolution group structure was obtained by sub-clustering, where we grouped disease terms into seven groups (Figure 8). Clusters names are based on EFO disease terms that include a large amount of cluster members as child terms (see materials and methods and Figures S10–S16); Table 6 lists disease terms per cluster. In addition to the clear separation of immune-related diseases from others, we also find a very homogeneous group consisting of mental and behavioral disorders, containing terms like schizophrenia (EFO: 0000692) and anxiety disorder (EFO: 0006788), and a group of skin cancers. The remaining three groups are more heterogeneous but with two of them containing several terms related to cardiovascular disease (EFO: 0000319) and digestive system disorders (EFO: 1000218), respectively. By design, similar tissues in each group drive SNP-disease associations, and we next examined which tissues play a role in each of the clusters.

**Table 6:** Disease groups based on DHS tissue-weights

For each disease group, disease terms are shown. The colored squares denote the disease groups in the EFO ontology.

**Figure 9:**
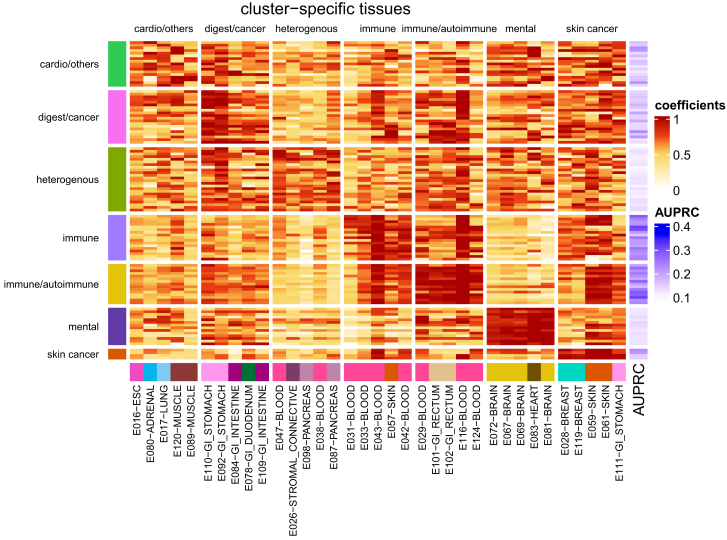
Heatmap of top-five tissue-weights for 111 diseases Regularized model coefficients (i.e., tissue weights) of five disease-cluster-specific tissues (columns) are shown for 111 diseases (rows). Coefficients are scaled by disease, and rows are grouped into sets of cluster-specific tissues (see materials and methods section). Bottom annotation shows tissue names of cluster-specific tissues (names are shown in the format of “tissue name” – “tissue group”; annotation on the left side shows disease cluster, and annotation on the right side shows model performance in terms of AUPRC.

In order to find group-specific tissues, we examined for each cluster the top five tissues that (1) contribute most to disease association and (2) are cluster specific (see materials and methods). The results are summarized in Figure 9; we note that both disease groups related to the immune system highlight blood tissues (such as E043: primary T helper cells from peripheral blood and E116: GM12878 lymphoblastoid cells; see Data SD23 for all names of standard epigenomes), with the group containing inflammatory bowel disease, Crohn disease (CD), and ulcerative colitis (UC) also containing rectum tissues (such as E101: rectal mucosa donor 29). Brain tissues contribute to disease associations for mental and behavioral disorders, skin tissues to skin cancer, and gastrointestinal/stomach tissue to the cluster with digestive system diseases. We also note that a clear association of specific tissues with a disease group correlates with the better classification performance of our model for SNP-disease association (Figure 9; for example, see the immune and immune/autoimmune clusters). We note, though, the corresponding tissue associations are not equally compelling for all clusters, as illustrated in the same figure. While the clusters we derive resemble broader disease groups, for each disease, a specific combination of tissues is used to derive whether a variant might be associated, and some tissues contribute to several clusters. For instance, one blood cell type (E116: GM12878 lymphoblastoid cells) contributes to both immune clusters but also to diseases in the digestive/cancer, heterogeneous, and skin cancer clusters. Another blood cell type (E043: primary T helper cells from peripheral blood) displays a similar pattern. Figure S9 shows the same heatmap as Figure 9 but for all tissues.

Overall, these results suggest that our modeling approach successfully identifies tissues with a role in disease etiology. Finally, we explore how our disease similarities relate to genetic similarities as measured by genetic correlation between diseases.

#### Model-based similarities are complementary to genetic correlation

**Figure 10:**
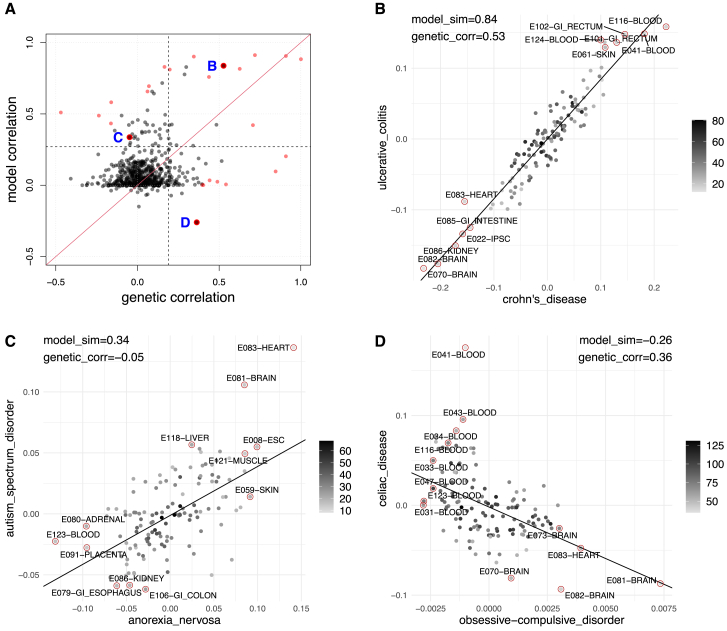
Genetic correlation and model similarity (A) Genetic correlation vs. model similarity for 595 disease pairs. Each point is a disease pair, where the x axis denotes the genetic correlation and y axis is the disease model similarity. For three quadrants, we highlight disease pairs, denoted by B, C, and D. (B–D) Scatterplot of tissue coefficients in three example disease pairs, where (B) shows Crohn disease vs. inflammatory bowel disease, (C) shows anorexia nervosa vs. autism spectrum disorder, and (D) shows celiac disease vs. obsessive-compulsive disorder. Lines denote a weighted linear regression line underlying our disease similarities. Color codes for the weight for each tissue when conducting weighted regression analysis.

Here, we compare the disease-disease similarities we derived ($s_{m}$) with genetic correlations from the GWAS Atlas ($s_{g}$), where genetic correlation measures shared genetic causes between two traits.^34^ For 6,105 possible disease pairs of the 111 diseases terms we study, estimates of genetic correlation for 595 pairs were available from the GWAS Atlas (see materials and methods). Overall, for these 595 disease pairs, we observe only a weak (but statistically significant) correlation between model similarities and genetic correlations (*r* = 0.32, *p* = 2.4E−15), where the scatterplot is shown in Figure 10A.

We also see that most disease pairs are not annotated with substantial genetic correlations or with high model-based similarities (90% of disease pairs have *s*_*m*_ < 0.25 and *s*_*g*_ < 0.2). Therefore, we explored three different regimes: disease pairs where both similarity measures are high (*s*_*m*_ ≥ 0.25 and *s*_*g*_ ≥ 0.20), pairs with high genetic correlations and low model similarity (*s*_*m*_ < 0.25 and *s*_*g*_ ≥ 0.20), and vice versa (quadrants indicated in Figure 10A, named quadrants B, C, and D). The top eight most extreme examples from each regime are summarized in Table 7. In the following, we discuss some examples in more detail. Specifically, we explore two immune system diseases for quadrant B, two mental or behavioral disorders for quadrant C, and one immune system disease and one mental or behavioral disorder for quadrant D. We note that the pairs we examine have no annotated parent-child relationships in the EFO.

**Table 7:** Example disease pairs of genetic correlation and model similarities

Disease 1	Disease 2	*s*_*g*_	*s*_*m*_	Quadrant
Inflammatory bowel disease	ulcerative colitis	1.00	0.88	B
Diabetes mellitus	type 2 diabetes mellitus	0.91	0.91	B
Crohn disease	inflammatory bowel disease	0.72	0.91	B
Sclerosing cholangitis	ulcerative colitis	0.63	0.82	B
Crohn disease	ulcerative colitis	0.53	0.84	B
Ankylosing spondylitis	sclerosing cholangitis	0.35	0.90	B
Inflammatory bowel disease	sclerosing cholangitis	0.44	0.76	B
Bipolar disorder	schizophrenia	0.71	0.42	B
Rheumatoid arthritis	systemic lupus erythematosus	−0.47	0.51	C
Celiac disease	systemic lupus erythematosus	−0.16	0.58	C
Sclerosing cholangitis	systemic lupus erythematosus	−0.24	0.49	C
Crohn disease	sclerosing cholangitis	0.17	0.83	C
Rheumatoid arthritis	sclerosing cholangitis	0.07	0.69	C
Crohn disease	rheumatoid arthritis	0.06	0.66	C
Systemic lupus erythematosus	ulcerative colitis	−0.16	0.43	C
Crohn disease	systemic lupus erythematosus	−0.10	0.49	C
Type 1 diabetes mellitus	type 2 diabetes mellitus	0.85	0.10	D
Diabetes mellitus	type 1 diabetes mellitus	0.91	0.20	D
Celiac disease	obsessive-compulsive disorder	0.36	−0.26	D
Diabetes mellitus	obesity	0.54	0.01	D
Obesity	osteoarthritis	0.49	0.02	D
Attention-deficit hyperactivity disorder	obesity	0.44	0.03	D
Attention-deficit hyperactivity disorder	osteoarthritis	0.40	0.00	D
Obesity	type 1 diabetes mellitus	0.40	0.00	D

This table shows the genetic correlation and model similarity for some disease pairs as we selected. For quadrants B, C, and D, we picked 8 disease pairs, where *s*_*g*_ + *s*_*m*_, *s*_*g*_ – *s*_*m*_, and *s*_*m*_ – *s*_*g*_ are the highest, respectively. *s*_*g*_, genetic correlation; *s*_*m*_, model similarity.

UC (EFO: 0000729) and CD (EFO: 0000384) have both high genetic correlation (*s*_*g*_ = 0.53) and model similarity (*s*_*m*_ = 0.84) (see Figure 10A). This suggests that they share genetic causes and that the same tissues are informative for SNP-disease association. While shared genetic causes for UC and CD have been pointed out (e.g., Yang et al.^35^), our model for SNP-disease association allows us to explore relevant tissue contexts. In Figure 10B, we show a scatterplot of tissue weights for both diseases, where color indicates the importance of each tissue to model similarity (see materials and methods). We observe that open chromatin in blood (E116: GM12878 lymphoblastoid cells; E124: monocytes-CD14+ RO01746 primary cells; and E041: primary T helper cells PMA-I stimulated) and rectum (E102: rectal mucosa donor 31) is positively associated with SNP-disease association in both diseases; this is consistent with a previous study where blood cell types were found to be relevant in many autoimmune diseases, including UC and CD.^36^ In addition, symptoms or complications in the rectum are also observed in UC and CD.^37^ Interestingly, open chromatin in gastrointestinal-intestine (E085: fetal intestine small) is negatively associated with SNP-disease association, along with other intestine tissues (E084: fetal intestine large and E109: small intestine, with the 61th and 86th smallest tissue weights, respectively, among 127 contexts). This indicates that the fetal intestine or small intestine might be less involved in UC and CD etiology compared to their juvenile and adult counterparts.

Autism spectrum disorder (ASD; EFO: 0003756) and anorexia nervosa (AN; EFO: 0004215) are an example where we observe a low genetic correlation (*s*_*g*_ = −0.05) and a moderate high model similarity (*s*_*m*_ = 0.34); a scatterplot of their tissue weights is shown in Figure 10C. Note that we did not choose one of the highlighted pairs in Table 7 for this quadrant because we already discussed an immune-system-related disease pair. We observe that both disease models give heart and brain tissue (E083: fetal heart and E081: fetal brain male) high tissue weights. This is consistent with the observation of brain abnormalities in ASD and AN.^38^^,^^39^ While the presence of the fetal heart is less intuitive, we note that children with abnormal heart development are more likely to develop ASD, suggesting a connection between the disease and the fetal heart.^40^ We also note that while the genetic correlation between ASD and AN is low, a link between the two diseases on the phenotypic level is being suggested^41^^,^^42^; the tissue context we identified could provide information about shared molecular aspects of disease etiology as well.

For obsessive-compulsive disorder (EFO: 0004242) and celiac disease (EFO: 0001060), we observe low model similarities (*s*_*m*_ = −0.26) and moderately high genetic correlation (*s*_*g*_ = 0.36); Figure 10D shows the scatterplot of tissue weights. Several studies have shown that nervous system disease and immune-related diseases have shared genetic backgrounds.^43^^,^^44^ However, in contrast to the other two examples, there is little relation between tissue weights in these two diseases. Blood cell types are highlighted in celiac disease, while brain and fetal heart tissues are highlighted in obsessive-compulsive disorder. For celiac disease, the top six tissue contexts are blood cells, including different types of T cells (E041: primary T helper cells PMA-I stimulated; E043: primary T helper cells from peripheral blood; and E034: primary T cells from peripheral blood) and lymphoblasts (E116: GM12878 lymphoblastoid cells), which is consistent with findings that alterations in T cells and lymphoblasts can lead to celiac disease.^45^^,^^46^

Overall, these examples illustrate that the disease similarities we derive are complementary to genetic correlation. In addition, tissue contexts highlighted by our tissue weights allow for biomedical interpretations of observed similarities (i.e., which are the relevant tissue contexts) and can be used to generate molecular hypotheses about disease etiology.

In summary, our results show that disease-specific variant prioritization performs well for non-coding GWAS variants, compared with organism-level approaches. We also demonstrate that disease-specific tissue weights are biomedically meaningful and can be used to generate hypotheses about disease mechanism. Therefore, we believe that this type of variant characterization is a useful tool for researchers studying the molecular and genetic causes of disease.

## Discussion

Most variant scores prioritize non-coding variants either at the level of the whole organism (e.g., CADD,^8^ GenoCanyon^26^) or provide tissue-specific scores (e.g., GenoSkyline,^11^ Fitcons2^12^). Here, we present a straightforward strategy to combine tissue-specific variant scores in a disease-specific manner. We show that for common genetic variants in the GWAS Catalog,^1^ our approach leads to a better performance than organism-level or tissue-specific scores (see Figure 6). Pre-computed disease-specific prioritization scores are available at https://doi.org/10.7910/DVN/AUAJ7K. Comparing different variant prioritization methods, we note that we use area under the precision-recall curve as an evaluation metric and that the performance of all methods is modest. We believe that is because our analysis (1) focuses explicitly on non-coding variants, (2) stratifies SNVs by disease phenotype, and (3) utilizes unbiased matching of control SNVs (SNPsnap matching; see control variants). Each of these points affects the SNV sets we use for our analysis and therefore the performance metrics we report. For transparency, we provide all disease-associated variants we use (with matched negatives) in Data SD1. As a more general point, we also note that associations reported in the GWAS Catalog contain causal as well as non-causal SNPs, which will also contribute to sub-optimal performance measures of all the variant scores we assess.

We included a comparison with the DIVAN method in our evaluation, which also includes comparing GenoCanyon with DIVAN. Part of this comparison is analogous to results reported in Chen et al.^22^; however, the performances we observed do not agree perfectly, as detailed in Data SD15. Broadly, looking at overlapping/matching disease terms, our results appear more favorable for GenoCanyon. These differences are likely due to different test sets used in the two evaluations (i.e., the GWAS Catalog [this study] vs. Genome-Wide Repository of Associations Between SNPs and Phenotypes).

We also note that there is other research associating variants with disease terms in a similar setting, notably PINES^23^ and LSMM.^47^ We did not compare directly with PINES because no pre-computed scores are available; also, we note that while the performance reported in this publication in terms of the AUROC is higher than our results, a less stringent un-matched test set of random/control variants was used in these analyses. For LSMM, we note that we leverage variants associated with EFO disease terms across studies, while LSMM uses summary statistics on a per-study basis. Using aggregate data from different studies allows our approach to consider parent-child relationships of the experimental factor ontology using variant aggregation (see disease-specific non-coding variant prioritization with organism-level variant scores is only moderately successful).

We demonstrate that our approach can be used to calculate similarities between disease terms (see disease similarities based on disease-specific tissue weights for non-coding variant prioritization). Since this similarity measure is derived from non-coding SNVs associated with disease, one could expect it is largely congruent with genetic correlation between disease traits. However, that is not the case (see Figure 10), most likely because we focus on a small subset of disease-associated SNVs reported in the GWAS Catalog. For example, obsessive-compulsive disorder and celiac disease have a high genetic correlation (*s*_*g*_ = 0.36) but do not share non-coding SNPs in the GWAS Catalog (and low model similarity, *s*_*m*_ = −0.26); on the other hand, ASD and AN have a low genetic correlation (*s*_*g*_ = −0.05) but share a number of significant SNPs in the GWAS Catalog (and relative high model similarity, *s*_*m*_ = 0.34). In addition, the interpretation of model similarity between disease terms is different from genetic correlation; high model similarity implies that disease-associated SNVs reside in DNA-accessible regions in an overlapping set of tissues, but the identity of individual SNVs (and whether they overlap) is inconsequential. For example, asthma and rheumatoid arthritis (RA) have only 15 shared SNPs (out of 732 and 1,283 SNPs in RA and asthma, respectively) but exhibit high model similarity (*s*_*m*_ = 0.53). This shows that model similarity between two diseases can involve similar tissues even if they do not share a genetic background. Further on, we note that estimates of genetic correlation also may depend on the study used. For example, systemic lupus erythematosus (SLE) has a negative genetic correlation (*s*_*g*_ = −0.47) with RA (and other inflammatory diseases) when using the SLE summary statistics from Julia et al.^48^ (as retrieved from the GWAS Atlas^34^), whereas another study (Lu et al.^49^) found SLE to have a positive genetic correlation (*s*_*g*_ = 0.41) with RA when using the SLE summary statistics from Bentham et al.^50^

We note that in our analyses, we used the EFO ontology to aggregate variants annotated in the NIH/EBI GWAS Catalog. That is, for each disease term, directly annotated variants were used, and, in addition, variants annotated to descendant terms in the ontology were also included. This approach allowed us to compile a more exhaustive set of variants per term. However, some amount of caution should be exercised when using disease models with more general terms, such as “cardiovascular disease,” for example, as they may encompass heterogeneous diseases.

Our approach is expected to improve as more variants are associated with disease and as disease associations get more refined. In addition, increasing amounts of epigenomics data, such as epimap^18^ and ENCODE5,^6^ could be incorporated, and they have the potential to improve the disease associations we learn. Further on, recent studies have shown benefits of including functional annotations in rare variant association analyses.^51^^,^^52^ In this context, our DHS-derived scores can be included as data-driven disease-specific combinations of functional genome annotations and included in this type of approach. It is future work to assess if this could be an approach to bring GWAS data and functional annotations to bear in rare variant analysis.

In summary, we have provided a straightforward method to leverage tissue-specific variant scores for disease-specific variant prioritization. We show that this approach performs well compared with current methods, and we show that the resulting association models are interpretable and lead to useful characterization of disease terms. Overall, our contributions are useful for the following two reasons: conceptually, because they highlight the value of disease-specific variant prioritization, and in addition, because we provide pre-computed prioritization scores for 111 disease terms that researchers can use in practice to interpret their variant data.

## Materials and methods

### Data sources and processing

#### Disease-associated variants

Disease-associated non-coding SNVs were retrieved from the NHGRI-EBI Catalog of human GWAS database (GWAS Catalog, v.2020-12-02, downloaded from https://www.ebi.ac.uk/gwas/docs/file-downloads). These data contained 122,396 unique non-coding SNPs spanning 2,782 phenotypes, where non-coding was defined as variants not overlapping with a protein-coding sequence (GENCODEv.36); we also excluded variants annotated as protein-coding sequence variants (e.g., missense variants, frameshift variants) as an SNP’s “functional class” in the GWAS Catalog. Further, variants in the GWAS Catalog are annotated with phenotypes using the EFO (https://www.ebi.ac.uk/efo).^53^ We focused on variants with phenotype terms annotated in the disease domain of the EFO (i.e., all terms/traits/phenotypes we consider are descendants of the term “disease” [EFO: 0000408, EFO v.3.24.0, accessed November 17, 2020]). Further on, SNPs in the human leukocyte antigen region and SNPs with a minor allele frequency (MAF) less than 1% in the European population as reported by the International Genome Sample Resource were excluded (as they cannot be matched to control SNPs with the SNPsnap approach; see below). Out of 31,103 SNVs, a total of 5,225 SNVs were removed. Finally, in our analyses, we restricted ourselves to phenotypes with at least 100 annotated non-coding SNPs. Data SD1 and SD2 contain the 111 phenotypes and 77,028 phenotype-associated SNPs we used in this study. We also grouped SNPs in LD blocks (SNPsnap, *r*^2^ ≥ 0.5) and identify SNPs with the minimum *p* value per block (“representative SNP”); we provide this information, which we use in some of the analyses described below, in Data SD2.

#### Control variants

For each disease-associated SNP, we generated matched control non-coding variants with MAF ≥ 1% using four different strategies, where the non-coding is again defined discussed above (disease-associated variants). The four strategies are as follows:

• Random: for each disease-associated SNP, we selected ten SNPs from common non-coding variants in 1000G (European, EUR) at random (i.e., equal probability for all SNPs) as controls.
• Transcription start site (TSS) matching: we processed common non-coding SNVs and selected a subset of these variants as controls, where the distribution of distances to the nearest protein-coding gene’s TSS are matched between control set and disease-associated SNPs (similar to GWAVA^27^). Specifically, we sorted all common non-coding SNPs by the distance to the nearest TSS and divided them into 50 bins, where each bin contains the same number of SNVs. Then, for each disease-associated SNP, we randomly selected ten control SNPs from the bin containing the disease-associated SNP’s distance to the nearest gene.
• SNPsnap matching: using SNPsnap,^25^ we matched control SNPs to disease-associated variants in terms of MAF, gene density (distance cutoff ld0.8), distance to the nearest gene TSS, and number of SNPs in LD. Our parameters for maximum allowable deviation were 5%, 50%, 20%, and 50%, respectively. We randomly selected ten control SNPs per disease-associated SNP form SNPsnap’s results, and we ensured that there were no duplicated control SNPs for different disease-associated SNPs. If there were less than 10 control SNPs returned by SNPsnap, then we kept all of the control SNPs. If no control SNPs were matched, then we removed the disease-associated SNVs (a total of 311 SNVs) from our analyses.
• SNPsnap-TSS matching: this is essentially the same as in SNPsnap matching but controlling only for the distance to the nearest genes (maximum allowable deviation: 20%); for three other attributes, “maximum allowable deviation” is set to 10,000%. We note that in both SNPsnap matching and SNPsnap-TSS matching, the distance is measured by the distance to the nearest gene, whereas for TSS matching, only protein-coding genes are considered.

In all four matching strategies, we excluded variants annotated in the GWAS Catalog as control SNPs. One control variant can only be matched to one disease-associated SNV. In our research, we chose SNPsnap matching for our main results, but we have compared the different performances of organism-level scores using the four different matching strategies (see supplemental methods and Figures S1 and S2). We also provided the four sets of control variants in Data SD3.

#### Additional data sources and variant scores

We used pre-computed SNP annotations from the following sources: CADD v.1.3 (http://krishna.gs.washington.edu/download/CADD/v1.3/1000G_phase3.tsv.gz); EigenPC v.1.1 (https://xioniti01.u.hpc.mssm.edu/v1.1); Fitcons2 (http://compgen.cshl.edu/fitCons2/hg19); GenoCanyon (http://genocanyon.med.yale.edu/GenoCanyon_Downloads.html); GenoSkylinePlus (http://genocanyon.med.yale.edu/GenoSkylineFiles/GenoSkylinePlus/GenoSkylinePlus_bed.tar.gz); GWAVA v.1.0 (ftp://ftp.sanger.ac.uk/pub/resources/software/gwava/v1.0/VEP_plugin/gwava_scores.bed.gz); LINSIGHT (http://compgen.cshl.edu/%7Eyihuang/tracks/LINSIGHT.bw); and DIVAN (https://sites.google.com/site/emorydivan). DHS accessibility: we downloaded Avocado-imputed^54^ DHS signals for 127 ENCODE biological contexts (tissues/cell types) from https://noble.gs.washington.edu/proj/avocado/data/avocado_full/DNase/.

### Tissue-weighted variant prioritization based on DNase1 hypersensitivity

#### A penalized logistic regression model for context-weighted score averaging

For predicting SNP’s associations with a disease term, we consider SNPs as observations, and each is described as a vector x ∈ R^*d*^ of variant scores in *d* tissues/contexts; we arrange vectors ${\left\{x_{i}\right\}}_{i=1}^{n}$ for *n* observations in a matrix $X\in R^{n\times d}$ together with a vector *y* of *n* binary entries, encoding for each SNP its association with a specific disease term (no = 0/yes = 1). In addition, we denote the average score (across contexts) for an SNP *i* by ${\bar{x}}^{i}$, which is also a basline score because it aggregates across contexts.

We use a logistic regression model of the form

(Equation 1) $$\log\frac{p_{i}}{1-p_{i}}=\alpha {\bar{x}}^{i}+\beta^{\prime }x^{i}\;s.t.\;\alpha \geq 0\text{,}$$

where $\alpha_{0}\in R$, $\alpha \in R_{+}$, and $\beta \in R^{d}$ are regression coefficients and *p*_*i*_ is the probability that SNP *i* is associated with a disease that is studied. We fit a regularized version of the negative log likelihood

(Equation 2) $${\mathrm{argmin}}_{\alpha ,\alpha_{0},\beta }-\frac{1}{2}\sum_{i=1}^{n}\left[\log\left(1-p_{1}\right)+y_{i}\;\log\frac{p_{i}}{1-p_{i}}\right]+\frac{\lambda {\left|\left|\beta \right|\right|}_{2}^{2}}{2}\text{,}$$

where the dependence on $\left\{\alpha ,\alpha_{0},\beta \right\}$ of the first term is through Equation 1. For large regularization parameters λ, this will yield small *β* → 0 and recover the baseline ($\bar{x}$) of unweighted averaging of context scores (scaled by a non-negative factor α). We implemented this approach using the R package glmnet (v.2.0-18^55^) and determined the regularization parameter via 5-fold cross-validation (cv.glmnet function) through maximizing the area under the (cross-validated) ROC curve. In the nested 5-fold cross-validation, we used the inner loop to select the regularization parameter λ and the selected λ to train and test the model in the outer loop. Class weights were employed to balance skewed class sizes.

#### Disease similarities from context-weighted score averaging

Context-weighted score averaging, as described above, results in disease-specific coefficient vectors ($\left\{\beta^{\left(i\right)}\right\}$, with *i* indexing disease terms), together with bootstrap estimates for the standard deviation of each coefficient (that can be arranged in corresponding vectors $\left\{\gamma^{\left(i\right)}\right\}$). Specifically, we use 5-fold cross-validation repeated 10 times, yielding 50 coefficient vectors for each disease. We use their mean for our estimate of $\beta^{\left(i\right)}$ and their standard deviation as an estimate of $\gamma^{\left(i\right)}$. For a pair of diseases $\left(d_{i},d_{j}\right)$, we then define a disease similarity through the similarity of associated coefficient vectors $\beta^{\left(i\right)}$ and $\beta^{\left(j\right)}$, taking into account our estimates of coefficient variability. Specifically, we fit a weighted linear regression model (i.e., regressing vectors $\beta^{\left(i\right)}$ on $\beta^{\left(j\right)}$), with regression weights taking into account coefficient variability as follows:

(Equation 3) $$w_{k}^{\left(i,j\right)}=\frac{1}{\sqrt{s_{k}^{i}s_{k}^{j}}}\;\text{and}\;s_{k}^{\circ }=\alpha \gamma_{k}^{\left(\circ \right)}+\left(1-\alpha \right)m\;\text{for}\;\circ \in \left\{i,j\right\}\text{,}$$

where we chose *m* to be the 25% quantile of all (estimated) standard deviations observed and α = 3/4. Therefore, $s_{k}^{i}$ and $s_{k}^{j}$ are shrunken versions of the standard deviations for the regression coefficients of disease *i* and disease *j* in tissue/context *k*, respectively. Finally, for disease pairs with a positive coefficient from the weighted linear regression, we take the coefficient of determination (*r*^2^) as a similarity measure; for disease pairs with a negative coefficient, we take −*r*.^2^ We note that for constant regression weights $\left\{w^{\left(i,j\right)}\right\}$, this is equal to the Pearson correlation between the coefficient vectors we obtain from context-weighted score averaging (i.e., $\text{cor}\left(\beta^{\left(i\right)},\beta^{\left(j\right)}\right)$.

### Variant prioritization performance

#### Tissue-weighted cross-validation performance

To measure the cross-validation performance of tissue-weighted scores, we use repeated cross-validation^56^ to reduce the variance (due to the random partitioning of data into 5-fold). Here, we repeat fold cross-validation 30 times and record the performance of each repeat. We later use the mean performance of the 30 repeats as the performance of that method, and we also show the variance in figures, such as in Figure 5.

#### Comparing organism-level scores

For each disease, we have disease-associated and control SNVs and corresponding pre-computed organism-level scores. With this setup, we calculate performance metrics of interest (AUROC and average precision) and obtain disease-specific performance metrics for each scoring approach. To compare performances between organism-level scores on the same disease, we use performance measures computed on 30 bootstrap samples (each bootstrap sample randomly contains 90% of disease and control variants) and then employ the Wilcoxon signed-rank test to test to assess differences in performance. This yields *p* values as reported in Data SD4.

With respect to aggregating comparisons across diseases, we note that disease terms can (and do) share SNVs, so performance metrics in different terms are not necessarily independent. Also, disease terms can vary substantially in the number of annotated SNPs. We again use the Wilcoxon signed-rank test^57^ on performance metrics (computed using all disease-associated and control SNVs for each disease term) to compare two organism-level variant score aggregates across diseases. This approach yields *p* values, as reported in Data SD5.

#### Comparing tissue-weighted scores

Tissue-weighted baseline scores (see above) are calculated in the same way as organism-level scores. For tissue-weighted scores with data-driven tissue-specific weighting (see above), we use cross-validated performance measure for each bootstrap sample and the same 30 bootstrap samples as when we compared between organism-level scores. And then, we use the same Wilcoxon signed-rank tests to measure the difference. For comparing scores aggregated across diseases, we again proceed analogous to organism-level scores and use a Wilcoxon signed-rank test on cross-validated disease-specific performance measures. Results are summarized in Data SD8 and SD9.

#### Comparing organism-level and tissue-weighted scores

For comparisons between organism-level and tissue-combined scores, we again use a bootstrap approach: for a specific disease term, we use the Wilcoxon signed-rank tests as discussed above to compare performance measures from organism-level scores with tissue-weighted scores. We note that this approach does not take into account (1) variability in the organism-level scores originating form variability of the data they are derived from or (2) the possibility that organism-level scores may have already used SNPs in their score derivation process, and we use them again for evaluation in their score derivation process. However, we do not expect these issues to substantially confound or results, and we note that incurred bias in our comparisons would expected to be in favor of organism-level scores. The results are summarized in Data SD6–SD11.

#### DIVAN performance assessment and comparison

To assess and compare our performance with DIVAN,^22^ we generated a test set of SNPs from the GWAS Catalog that were (1) added after DIVAN had been published (i.e., after May 28, 2016), (2) not present in the database used to train DIVAN (association result browser: https://www.ncbi.nlm.nih.gov/projects/gapplus/sgap_plus.htm), (3) not within a 1 kb distance around SNPs used to train DIVAN, and (4) annotated to a disease phenotype addressed by DIVAN. Control SNPs were generated using SNPsnap matching, as described above. To be able to satisfy criterion (4), we mapped our disease terms (EFO terms) to disease terms used by DIVAN (MeSH terms) using the EMBL-EBI Ontology Xref Service (https://www.ebi.ac.uk/spot/oxo/, retrieved on April 19, 2020) and were able to resolve 41 out of 45 terms (Data SD12). Of these, we keep terms with 20 or more disease-associated SNPs in the test set and 50 or more SNPs in a training set that we also constructed (see below), yielding 29 overall disease phenotypes that we used in our analysis. In order to fairly compare DIVAN with our logistic regression approach, we constructed a training set using disease-associated SNPs from the GWAS Catalog and the Phenotype-Genotype Integrator (https://www.ncbi.nlm.nih.gov/gap/phegeni),^58^ excluding SNPs in the test dataset describe above or SNPs within 1 kb around test SNPs. Data SD13 summarizes test and training data used for this analysis. The results are summarized in Data SD14.

#### Performance assessment using chromosome holdout

To assess the performance of our DHS tissue-weighted score, we also used a chromosome hold-out strategy with test SNPs on different chromosomes from training data. Specifically, for each disease, we choose a set of chromosomes that contains approximately 20% SNVs with a 1/10 positive-to-negative ratio (the same as the cross-validation setting) as a test set. The selection of test chromosomes is performed for each disease term separately, as disease-associated SNPs differ. To automate the procedure, we deployed (binary) linear programming to pick out chromosomes in the test set for each disease. Specifically, for each disease term, we solve the optimization problem

$${\mathrm{argmax}}_{{\left\{x_{i}\right\}}_{i=1}^{22}}\sum_{i=1}^{22}c_{i}x_{i}\text{,}$$

(Equation 4) $$\text{subject}\;\text{to}\sum_{i=1}^{22}w_{i}^{+}x_{i}\leq 0\;\text{and}\;x_{i}\in \left\{0,1\right\}\text{,}$$

where $\left\{x_{i}\right\}$ are binary indicator variables whether a chromosome is included in the test/hold-out set, $w_{i}^{+}$ and $w_{i}^{-}$ are the fractions of disease-associated ($w_{i}^{+}$) and control SNPs ($w_{i}^{-}$) on chromosome *I*, and weights in the objective function are defined as $c_{i}=w_{i}^{+}-\left|w_{i}^{+}-w_{i}^{-}\right|$. This approach selects, for each disease term, a set of chromosomes to hold out that contain about 20% of disease-associates SNPs and that approximately reflects the overall imbalance between disease-associated and control SNPs. Figures S17 and S18 contain performance evaluations on chromosome hold-out sets.

#### Performance assessment using one SNP per LD block

To assess the effect of SNP correlation on our results, we also performed analyses using only a single representative SNP per LD block (defined by *r*^2^ ≥ 0.5; see disease-associated variants). The results are shown in Figures S19 and S20.

### Comparison with genetic correlation

We retrieved genetic correlation values of disease pairs from the GWAS Atlas.^34^ To be able to use these data, we mapped EFO disease terms (used in the NIH-NCBI GWAS Catalog and in our study) to terms used in the GWAS Atlas study. To do so, we extracted synonyms of each EFO term (as listed on EFO ontology) and compared each synonym to the “trait” and “uniqtrait” columns in the GWAS Atlas data. All matches (with one tolerated letter substitution) were used.

In this approach, a single EFO term can map to multiple GWAS Atlas traits and studies. To estimate the genetic correlation between two EFO terms (say, *d*_*i*_ and *d*_*j*_), we use a weighted combination of genetic correlation values:

(Equation 5) $$r_{g}\left(d_{i},d_{j}\right)=\sum_{l,m}W_{lm}r_{g}\left(s{\left(d_{i}\right)}_{l},s{\left(d_{j}\right)}_{m}\right)\text{,}$$

where $r_{g}\left(\cdot ,\cdot \right)$ is the genetic correlation of two diseases, ${\left\{s\left(d_{i}\right)\right\}}_{i=1}^{r}$ and ${\left\{s\left(d_{j}\right)\right\}}_{j=1}^{s}$ are the GWAS Atlas studies that are mapped to EFO term *d*_*i*_ and *d*_*j*_, respectively, and *w*_*lm*_ is a weight for each combination of the GWAS Atlas studies accounting for the sample sizes of different studies used to estimate genetic correlation values. We choose

(Equation 6) $$w_{lm}=\tilde{w}\left(s{\left(d_{i}\right)}_{l}\right)\cdot \tilde{w}\left(s{\left(d_{j}\right)}_{m}\right)\text{,}$$

where

(Equation 7) $$\tilde{w}\left(s{\left(d_{i}\right)}_{l}\right)=size\left(s{\left(d_{i}\right)}_{l}\right)/\sum_{k}\text{size}\left(s{\left(d_{i}\right)}_{k}\right)\text{,}$$

where “size” denotes the sample size of a study. This scheme puts higher weights on studies with large sample sizes and smaller weights on studies with smaller sample sizes.

### Notes about epimap comparison, cluster annotation, and display

#### Epimap trait-tissue association

To create Table 5, we obtained the latest SNP-centric GWAS enrichments table from the EpiMap Repository at http://compbio.mit.edu/epimap/. We retrieve tissues with adjusted *p* values for each disease. We map the tissue names used in our study (Standard Roadmap Epigenomes, as labeled by EID) to tissue names used in epimap (biosamples, as labeled by BSS biosample ID) by adapting the scripts from https://github.com/cboix/EPIMAP_ANALYSIS/blob/master/metadata_scripts/get_roadmap_mapping.R. If there are more than one biosample tissues mapped to roadmap tissues, then we report the *p* value of the tissue with the most significant result.

#### Disease group names

To name each cluster/group of diseases/EFO terms in Table 6, we choose the EFO term that contains most of the cluster/group members. In Data SD21, we summarize the terms with high term frequency in each cluster, where term frequency is the fraction of the descendant terms present. For example, the EFO term “immune system disease” (EFO: 0000540) has a term frequency of 0.588 in the “immune-1 cluster”; this means that 58.8% of EFO terms in that cluster are descendants of EFO: 0000540. We exclude the terms that are overly broad such as the term “disease” or “experimental factor ontology.” For each cluster, we rank the cluster member EFO terms using term frequency and select as the name a meaningful term with the high term frequency. For one cluster where no term had high frequency, we chose the name “heterogeneous.”

We also show a diagrams of EFO disease term relationships in each cluster in Figures S10–S126. Occasionally, we include ancestor EFO terms not present in the cluster in a diagram, which are marked by asterisks.

#### Dimension reduction and coefficient heatmap

UMAP plot: the two-dimensional UMAP plot of the 111 EFO disease terms in Figure 8 is based on disease similarities based on context-weighted score averaging (see disease similarities from context-weighted score averaging). The umap function of the uwotR package was used with the parameters *n* neighbors = 15, ret model = TRUE, and PCA center = FALSE.

Coefficient heatmap: the heatmap in Figure 9 displays coefficient vectors of models for disease association (see a penalized logistic regression model for context-weighted score averaging), normalized for each disease. Specifically, for each disease and tissue coefficient *x*_*i*_,

(Equation 8) $$\tilde{x_{i}}=\left\{ \begin{array}{cc} \left(x_{i}-\;x_{\min\;}\right)/x_{95} & x_{i}\leq x_{95}\\ 1 & x_{i}>\;x_{95} \end{array}\right.\text{,}$$

where *x*_min_ is the minimum coefficient for a disease and *x*_95_ is the 95% quantile.

Cluster-associated tissues: for each cluster, we show the top five tissues that are most associated with the cluster (Figure 9). To identify these tissues, we conduct a two-sample Wilcoxon test (one-sided) on every tissue, where we compare normalized tissue coefficients for this cluster to the other with the highest coefficients on average. The five tissues with the smallest *p* value are then selected as the top five tissues.

Tissue-associated clusters: for the heatmap with all tissues in Figure S9, we assigned a cluster to each tissue. For each tissue, we calculated the median (across disease terms of a cluster) of the normalized coefficients for all clusters; the cluster with the highest median was assigned.

## Data and code availability

Public data repositories were used as detailed in the materials and methods section, and data underlying the tables and figures are available as supplemental information online. 25-bp-resolution tissue-weighted DHS scores are available for download at https://doi.org/10.7910/DVN/AUAJ7K, and the computer code used to generate the analyses presented is available at https://github.com/kostkalab/nc-gwassnps-score_manuscript.

## Web resources

CADD v.1.3, http://krishna.gs.washington.edu/download/CADD/v1.3/1000G_phase3.tsv.gz
EpiMap, http://compbio.mit.edu/epimap/
DIVAN, https://sites.google.com/site/emorydivan
EigenPC v.1.1, https://xioniti01.u.hpc.mssm.edu/v1.1
EMBL-EBI EFO, https://www.ebi.ac.uk/efo
Fitcons2, http://compgen.cshl.edu/fitCons2/hg19
GenoCanyon, ttp://genocanyon.med.yale.edu/GenoCanyon_Downloads.html
GenoSkyline, http://genocanyon.med.yale.edu/GenoSkylineFiles/GenoSkylinePlus/GenoSkylinePlus_bed.tar.gz
GitHub, https://github.com/cboix/EPIMAP_ANALYSIS/blob/master/metadata_scripts/get_roadmap_mapping.R
GWAVA, ftp://ftp.sanger.ac.uk/pub/resources/software/gwava/v1.0/VEP_plugin/gwava_scores.bed.gz
LINSIGHT, http://compgen.cshl.edu/%7Eyihuang/tracks/LINSIGHT.bw
NCBI gapplus, https://www.ncbi.nlm.nih.gov/projects/gapplus/sgap_plus.htm
DNase Hypersensitivity, https://noble.gs.washington.edu/proj/avocado/data/avocado_full/DNase/

## Supplemental information

Document S1. Supplemental methods, Tables S1–S7, Data SD1–SD25, and Figures S1–S20

Data S1. Legends for Supplemental Data SD1 - SD25

Document S2. Article plus supplemental information
