# Supplementary material for: Disease-specific prioritization of non-coding GWAS variants based on chromatin accessibility
Source: HGG Adv. 2024 May 21;5(3):100310. doi: 10.1016/j.xhgg.2024.100310 (PMC11259938; doi:10.1016/j.xhgg.2024.100310)
Supplement: Document S2. Article plus supplemental information [file mmc3.pdf]

HGG Advances

ARTICLE

Disease-specific prioritization of non-coding GWAS variants based on chromatin accessibility

Qianqian Liang,[1,2] Abin Abraham,[3] John A. Capra,[4] and Dennis Kostka[1,5,*]

Summary

Non-protein-coding genetic variants are a major driver of the genetic risk for human disease; however, identifying which non-coding variants contribute to diseases and their mechanisms remains challenging. *In silico* variant prioritization methods quantify a variant's severity, but for most methods, the specific phenotype and disease context of the prediction remain poorly defined. For example, many commonly used methods provide a single, organism-wide score for each variant, while other methods summarize a variant's impact in certain tissues and/or cell types. Here, we propose a complementary disease-specific variant prioritization scheme, which is motivated by the observation that variants contributing to disease often operate through specific biological mechanisms. We combine tissue/cell-type-specific variant scores (e.g., GenoSkyline, FitCons2, DNA accessibility) into disease-specific scores with a logistic regression approach and apply it to ~25,000 non-coding variants spanning 111 diseases. We show that this disease-specific aggregation significantly improves the association of common non-coding genetic variants with disease (average precision: 0.151, baseline = 0.09), compared with organism-wide scores (GenoCanyon, LINSIGHT, GWAVA, Eigen, CADD; average precision: 0.129, baseline = 0.09). Further on, disease similarities based on data-driven aggregation weights highlight meaningful disease groups, and it provides information about tissues and cell types that drive these similarities. We also show that so-learned similarities are complementary to genetic similarities as quantified by genetic correlation. Overall, our approach demonstrates the strengths of disease-specific variant prioritization, leads to improvement in non-coding variant prioritization, and enables interpretable models that link variants to disease via specific tissues and/or cell types.

Introduction

Characterizing non-coding genetic variants in the human genome is essential for making progress toward better understanding the genetic components of disease, as ~90% of disease-associated variants discovered by genome-wide association studies (GWASs) are located in non-protein-coding regions.[1] Furthermore, whole-genome sequencing discovers disease-associated variants genome-wide[2,3] and is increasingly becoming an assay of choice. Therefore, approaches for characterizing and prioritizing non-coding variants can be expected to play an increasingly important role, especially when assessing discovered variants in the context of functional follow-up experimental studies.

Efforts to computationally characterize and better understand non-coding variants take advantage of sequence, functional genomics, comparative genomics, and epigenomics data,[4–6] and more. These data are combined and used to train and develop supervised and/or unsupervised models that attempt to quantify a variant's impact.[7] We find it conceptually useful to distinguish between variant scores that model overall impact (that is on the level of the whole organism, organism-level scores) and scores that quantify impact in a specific context, like a tissue or a cell type (i.e., tissue-level scores). Examples of methods for obtaining organism-level scores are CADD,[8] Eigen,[9] and LINSIGHT,[10] while scores from methods like GenoSkyline,[11] Fitcons2,[12] and FUN-LDA[13] are tissue specific. In addition, GenoNet[14] is an approach that leverage existing disease-specific scores together with genome-wide functional annotation to better predict organism-level and tissue-specific variant impact. RegBase,[15] on the other hand, combines existing scores for broad categories like pathogenic, cancer-driver, or regulatory variants. Often, interest in a set of variants is from the perspective of studying a specific disease. In that case, organism-level scores are likely to be overly general. That is, a variant's impact might be considered high because it disrupts the functional role of a sequence element. However, that functional role may be unrelated to the disease of interest. In one study, for instance, organism-level scores like CADD and DANN were unable to discover an enrichment signal for brain-related traits, while context-specific variant scores focusing on relevant tissues were successful.[16] This demonstrates that tissue-specific scores can address the issue of disease specificity to some extent. Therefore, approaches like TSEA-db[17] and efforts like EpiMap[18] aim to identify disease-relevant tissues. Despite these approaches

[1]Department of Computational & Systems Biology and Center for Evolutionary Biology and Medicine, University of Pittsburgh School of Medicine, Pittsburgh, PA, USA; [2]Department of Human Genetics, University of Pittsburgh School of Public Health, Pittsburgh, PA, USA; [3]Children's Hospital of Philadelphia, Philadelphia, PA, USA; [4]Department of Epidemiology & Biostatistics and Bakar Computational Health Sciences Institute, University of California, San Francisco, San Francisco, CA, USA
[5]Lead contact
*Correspondence: kostka@pitt.edu
https://doi.org/10.1016/j.xhgg.2024.100310.
© 2024 The Authors. This is an open access article under the CC BY license (http://creativecommons.org/licenses/by/4.0/).

and their successes, it remains the case that aspects of disease-relevant tissues typically remain unknown, and often, more than one tissue is implicated with a specific trait (termed "multifactorial" and "polyfactorial" traits).[18] This suggests the use of disease-specific variant scores that characterize variants in the context of a specific disease phenotype of interest.

Computational methods for disease-specific variant prioritization do exist. Some approaches are geared toward one disease (e.g., congenital heart disease,[19] amyotrophic lateral sclerosis[20]) or toward a specific class of diseases (e.g., autoimmune diseases[21]). This focus prevents them from being readily adapted to other disease types. Others, like DIVAN,[22] PINES,[23] and ARVIN,[24] cover a broader range of disease types. Of these, ARVIN requires *a priori* knowledge of disease-relevant tissues, whereas DIVAN and PINES do not. PINES uses an enrichment-based method to predict and up-weight disease-relevant tissues/cell types, whereas DIVAN uses a more complex machine learning algorithm. The PINES approach has been evaluated on a relatively small set of traits, while DIVAN's more complex model renders understanding the relationship between different tissues and diseases difficult.

In this work, we derive disease-specific variant scores by combining published tissue-specific scores (Figure 1). We use a carefully regularized logistic regression approach to derive data-driven disease-specific combination weights, which allow us to better associate variants with disease. In addition, they enable us to quantify a similarity between different disease phenotypes. Using the NHGRI-EBI GWAS Catalog,[1] we compiled a benchmark dataset containing about 25,000 phenotype-associated non-protein-coding single-nucleotide variants (SNVs) across 111 disease phenotypes (together with matched random controls). We then demonstrate that using disease-specific combination weights outperforms conventional organism-level approaches, that our interpretable model has competitive performance, and that it enables a disease similarity measure that captures information complementary to established measures like genetic correlation.

Results

Non-coding GWAS variants associated with disease phenotypes and matched controls

In order to study variant prioritization methods, we created a dataset of "positive" (i.e., disease-associated) non-coding variants, matched with a random set of "negative" or "control" variants. This setup allowed us to quantitatively assess prioritization methods based on their performance in discriminating positive from control variants.

Disease-associated non-coding SNVs

We used a subset of SNVs reported in the EBI/NIH GWAS Catalog[1] to compile an inventory of disease-associated non-coding variants. Specifically, we focused on reported variants that (1) do not overlap protein-coding sequence (see materials and methods) and (2) are associated with a disease phenotype as noted in the Experimental Factor Ontology (EFO) trait description, which is provided within the catalog. We define disease phenotypes as descendants of the EFO term "disease" (EFO: 0000408). Focusing on disease terms with at least 100 annotated SNVs resulted in 26,080 associations involving 20,656 SNVs and 67 disease phenotypes. The EFO provides parent-child relations between disease terms (parent = more general, child = more specific), and propagating SNVs from child terms to parent terms increased the number of disease phenotypes with at least 100 SNVs, resulting in 77,028 associations between 25,516 SNVs and 111 diseases. We find that most of the SNVs we recover are located in intronic (60.5%) and intergenic (25.8%) sequences (Figure 2A) and that a majority of SNVs are directly annotated to a single disease phenotype (Figure 2B). After propagating annotated SNVs from child to parent terms, SNV-to-disease annotations become predominantly many:many (Figure 2B). Data SD1 lists disease terms and corresponding numbers of disease-associated SNVs.

Control SNVs

For each disease-associated SNV, we selected ∼10 matched control SNVs using a re-implementation of the SNPsnap approach[25] while avoiding duplicate control SNVs across the overall dataset (see materials and methods). This yielded 255,137 control SNVs (for some disease-associated SNVs, we could not retrieve the full ten control SNVs). With these results, we have access to data for 111 disease terms, containing disease-associated SNVs together with matched controls. Data SD2 and SD3 contain information about all disease and control SNVs used in this study, respectively.

Disease-specific scores improve non-coding variant prioritization

Before we describe our approach for disease-specific variant scoring, we evaluated the performance of existing organism-level scores on our dataset in disease-specific non-coding variant prioritization with organism-level variant scores is only moderately successful. We then detail our approach in disease-specific aggregation weights for tissue-specific variant scores and DHS scoring outperforms other scores, before we show that it outperforms current organism-level scores in DHS tissue-weighted scoring outperforms organism-level variant scores. Our overall approach is summarized in Figure 1.

Disease-specific non-coding variant prioritization with organism-level variant scores is only moderately successful

We assessed how well current commonly used organism-level variant scores are able to prioritize disease-associated vs. control SNVs for the 111 disease terms we studied. Figure 3 summarizes the results, where boxplots of two performance measures (area under the receiver operator characteristic curve [AUROC] and average precision [= area under the precision-recall curve]) are

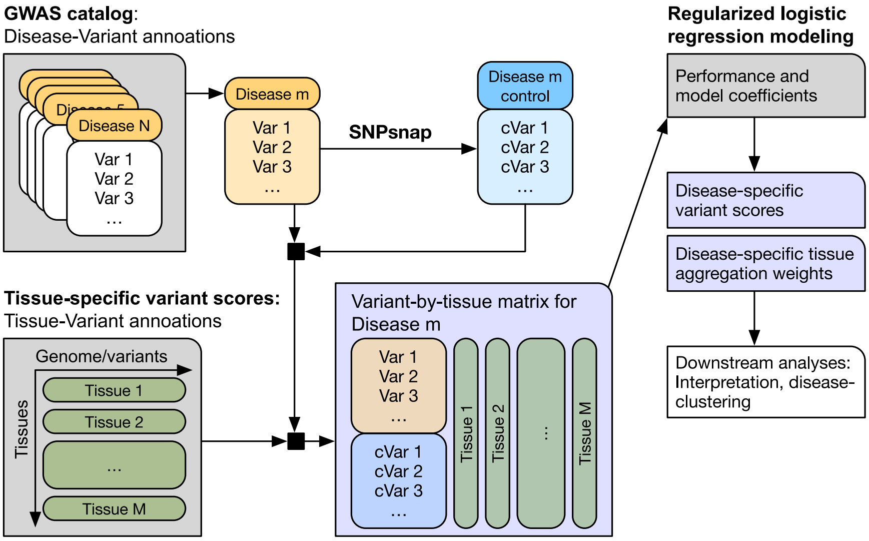

Figure 1. Summary of our approach
Variant annotations from the GWAS Catalog were augmented with disease-specific control variants using SNPsnap. For each disease, a logistic regression model relates disease-variant association to tissue-specific variant scores. Results are disease-specific variant scores and disease-specific tissue aggregation weights that can serve as input for further analyses, like the calculation of disease-disease similarities.

shown for CADD,[8] Eigen,[9] GenoCanyon,[26] GWAVA,[27] and LINSIGHT[10] scores. We find that organism-level scores, while improving upon random guessing, are only moderately successful in correctly prioritizing disease-associated non-coding variants. Comparing variant scores with one another, we find that relative performance differences appear overall robust with respect to the metric employed (AUROC vs. average precision). It is qualitatively visible that CADD performs less favorably than other methods but also that there are differences between them. We therefore compared performance between different scores in more detail.

We studied the performance of different scores at two levels of resolution: in aggregate across all disease terms and for each disease term separately. For both approaches, we used Wilcoxon signed-rank tests to decide whether one score significantly outperforms another score (= significant p value) or whether performance is tied (=non-significant p value); see the materials and methods section. The results are summarized in Table 1. We find that GenoCanyon has better performance compared with other variant scores, followed by LINSIGHT, GWAVA, and Eigen, while CADD is consistently outperformed by the other methods. Performance differences between LINSIGHT, GWAVA, and Eigen are not significant when aggregating across disease terms (last three columns in Table 1); however, when counting individual terms, LINSIGHT has the most wins and fewest losses, while Eigen has the most losses and fewest wins, leading to the ordering displayed in Table 1. Data SD4 and SD5 contain results for all comparisons. Overall, these quantitative results are in line with the visual impression from Figure 3. Next, we investigated if the performance of organism-level variant scores could be improved by using tissue-specific scoring approaches.

Disease-specific aggregation weights for tissue-specific variant scores

We studied three tissue-specific scores for variant prioritization to explore if their usage can improve the performance of organism-level scores. Specifically, we used Genoskyline[11] and Fitcons2[12] as scores designed to prioritize variants, and we also evaluated DNase I hypersensitivity (DHS) profiles from the ENCODE project.[6] All of these scores are available for contexts[5] spanning a diverse set of cell and tissue types, including heart, brain, immune cells, and more.

For each tissue-specific score, we assess two approaches to prioritize variants. First, as a baseline approach, we aggregate scores across tissues in a disease-agnostic way. That is, for a specific variant, we average scores at the variant position across all tissues (termed tissue-mean), essentially producing an organism-level type score, independent of the disease term under consideration. Second, we aggregate scores across tissues in a disease-specific way. Briefly, we train a regularized logistic regression model for each disease term that learns disease-specific tissue aggregation weights. In a nested cross-validation setup, learned weights are then applied to held-out variants, allowing for a fair performance assessment of this approach (termed tissue-weighted); see the materials and methods section. Figure 4 summarizes our findings.

In Figure 3A, we show the tissue-mean performance (measured by average precision) for the three scores we study on the left and tissue-weighted performance on the right. For all three scores, tissue-weighted significantly outperforms tissue-mean (Wilcoxon signed-ranks test, $p < 0.0001$). Figure 4B shows tissue-mean vs. tissue-weighted comparisons for each score, and we see that in almost all disease terms, tissue-weighted outperforms tissue-mean. See Data SD6 and SD7 for tissue-mean vs. tissue-weighted performances for each disease term and for aggregated performances across all disease terms. The improvement remains evident if we limit disease-associated SNVs to one variant per linkage disequilibrium (LD) block and also when we ensure that the SNVs in the training and test datasets are not on the same chromosome (see Figures S17–S20 and the supplemental information for more details).

While the performance gain for tissue-weighted is broadly consistent across diseases, for some it is more pronounced than for others. To illustrate this

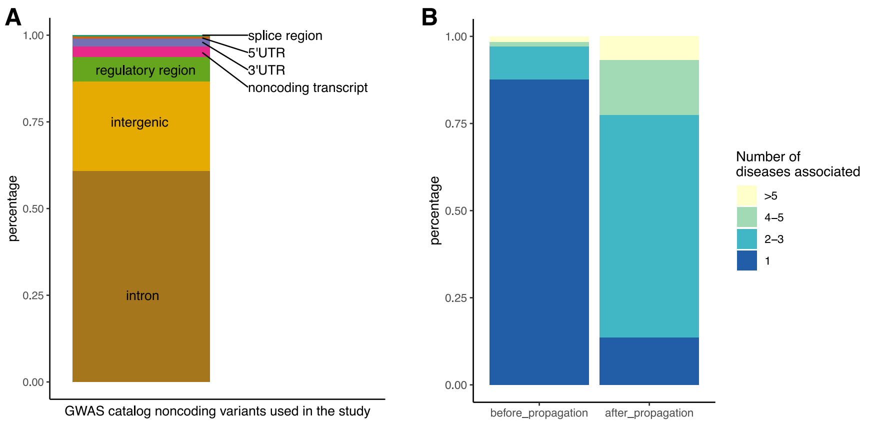

Figure 2. Disease-associated non-coding SNVs
(A) Genomic context of non-coding SNVs used in this study.
(B) Percentage of the SNVs used that are annotated to 1, 2–3, 4–5, or more than 5 disease phenotypes before and after propagating SNV-phenotype associations according to EFO parent-child annotations. Genomic context annotation is adapted from the CONTEXT column from the GWAS Catalog, where we combine splice donor, splice region, and splice acceptor variants into splice variants and combine transcription factor binding variants and regulatory regions variants into regulatory region variants.

observation, we selected four disease terms with a high performance gain, four terms with a medium gain, and four terms where we observed the least gain (best improvement, ranking 1–4; middle improvement, ranking 20–23; least improvement, ranking 108–111). Figure 5 shows our findings, where variability in tissue-weighted performance induced by varying train-test-fold splits during cross-validation is also displayed. We see that for celiac disease (EFO: 0001060), systemic scleroderma (EFO: 0000717), chronic lymphocytic leukemia (EFO: 0000095), and sclerosing cholangitis (EFO: 0004268), performance is consistently improved for all three tissue-weighted scores, while for retinopathy (EFO: 0003839), endometriosis (EFO: 0001065), diabetic nephropathy (EFO: 0000401), and HIV-1 infection (EFO: 0000180), we find no improvement. We also note that disease terms with a pronounced improvement appear to have a better baseline (i.e., tissue-mean) performance than disease terms where we find little or no benefit of the tissue-weighted approach. The improvement for diseases shown in Figure 5 is largest for DHS, but, consistent with Figure 4, we see improvement for Fitcons2 and GenoSkyline as well.

DHS scoring outperforms other scores

Having used three tissue-specific scores to derive disease-specific variant scores, we assessed their relative performance in this context. To quantify relative performance, we proceed similarly to organism-level scores. Focusing on pairwise comparisons, we find that disease-specific scores derived from DHS scores outperform GenoSkyline and Fitcons2 for most disease terms and on average (see Table 2). This can also be observed in Figures 4 and 5, which often show higher average precision values for DHS than for the other two scores. Notably, the baseline (i.e., tissue-mean) performance of DHS does not appear significantly better than that of Genoskyline (Figure 4). Data SD8 and SD9 contain details for comparisons between DHS, Fitcons2, and GenoSkyline for all disease terms. Next, we explored whether disease-specific tissue weights outperform organism-level scores.

DHS tissue-weighted scoring outperforms organism-level variant scores

To compare the DHS tissue-weighted score with organism-level scores, we directly contrasted their performance. Similar to before, Table 3 summarizes DHS "wins" (= significantly better performance of DHS tissue-weighted,

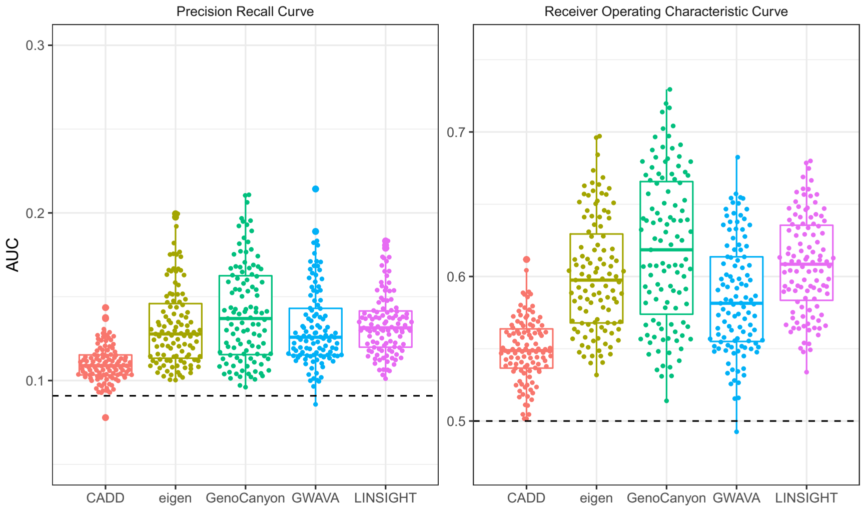

Figure 3. Organism-level variant scores are moderately successful in prioritizing noncoding disease-associated variants
Different organism-level variant prioritization scores are shown on the x axis, and the y axis displays performance in terms of average precision (area under the precision recall curve, left) and area under the receiver-operator curve (right). Each point represents a specific disease term from the experimental factor ontology. Horizontal lines spanning datasets show expectations under random guessing.

Table 1. Relative performance of organism-level variant scores

Score/method	By disease term Wins	Losses	Ties	Aggregated Wins	Losses	Ties
GenoCanyon	307	106	31	4	0	0
LINSIGHT	281	146	17	1	1	2
GWAVA	221	196	27	1	1	2
eigen	219	201	24	1	1	2
CADD	24	403	17	0	4	0

Wins, losses, and ties refer to significantly better (or worse, or tied) performance across all possible pairings (see materials and methods). Columns 2-4 summarize separate comparisons for each disease term (for each row, there are four other methods and 111 terms, i.e., 444 comparisons), while the last three columns represent the results of comparisons between scores aggregated across terms. Average precision was used as the performance metric, and Wilcoxon signed-rank tests were used to determine wins and losses (p values equal or larger than 0.05 are reported as ties).

$p \leq 0.05$), losses, and ties, compared with five organism-level variant scores, individually (i.e., per disease term) and aggregated across disease terms. In addition, Table S4 summarizes pairwise comparisons between tissue-weighted DHS and each organism-level score. We find that DHS tissue-weighted outperforms all organism-level scores in the aggregated analyses and that it outperforms all other scores on the majority of disease terms (it only performs significantly worse than any other score in 44 out of 550 comparisons).

GenoCanyon is the most competitive organism-level score, where DHS is significantly better for 92 terms out of 111 (~83%). Interestingly, LINSIGHT performs better against DHS than GenoCanyon, which is the best overall performing organism-level score (see Table S4). Data SD10 contains detailed results for each comparison. We also find that DHS outperforms organism-level scores when aggregating over disease terms (also see Data SD11).

To illustrate the gain in performance, we selected four example disease terms where disease-specific variant prioritization yielded high improvements, medium improvements, comparable performance, and worse performance, respectively. Selection was based on ranking differences between DHS and GenoCanyon: best improvement, ranks 1–4; medium improvements, ranks 25–28; comparable performance, ranks 64–67; and GenoCanyon better, ranks 108–111. The results are summarized in Figure 6, where we find substantial improvements using tissue-weighted scoring for systemic scleroderma (EFO: 0000717), celiac disease (EFO: 0001060), sclerosing cholangitis (EFO: 0004268), and multiple sclerosis (EFO: 0003885), for which we have already noticed substantial improvement of DHS tissue-weighted over DHS tissue-mean. Disease terms where GenoCanyon performs better include venous thromboembolism (EFO: 0004286), diverticular disease (EFO: 0009959), non-small cell lung carcinoma (EFO: 0003060), and lung adenocarcinoma (EFO: 0000571).

To make DHS tissue-weighted scores available, we generated pre-computed scores for 111 diseases at every base across the genome (for chromosomes 1–22, available at https://doi.org/10.7910/DVN/AUAJ7K). Scores were calculated at 25 bp resolution using the hg19 assembly, in the same resolution and on the same assembly as the DHS scores.

DHS scoring performs well compared with DIVAN

Here, we compare the performance of tissue-weighted DHS scoring with DIVAN,[22] a disease-specific variant score for 45 diseases. DIVAN is based on a more complicated feature selection and ensemble learning framework, and it uses a variety of other functional genomics features in addition to DHS. To compare our method with DIVAN, we mapped EFO disease terms to MeSHs and use MeSH terms in this section (see Data SD12). Because DIVAN is used as a supervised learning approach, and because the published model was trained using GWAS SNVs, it was necessary to create specific train and test datasets to ensure a meaningful comparison between tissue-weighted DHS and DIVAN.

Therefore, to assess the performance of both DIVAN and DHS, we created a test set of disease-associated variants (and their matched controls) that were published later than 2016 (DIVAN's publication date). That is, these variants are unlikely to have been a part of DIVAN's training data. We also created a training set for DHS tissue-weighted containing only SNVs published prior to 2016. This resulted in training data that (1) are distinct from the test set and (2) draw on similar information that was available for DIVAN's training. Further on, we only selected disease terms for this training/test data combination where at least 20 term-associated SNVs were present in the training data and at least 50 SNVs were present in the test data. This approach yielded 29 disease terms for this analysis. We then re-trained tissue-weighted DHS on this training data and compared it with DIVAN on the test data. In addition, we added the organism-level GenoCanyon score as a reference.

To assess performance, we performed all pairwise comparisons for each disease term and evaluated performance based on average precision. Table 4 summarizes observations, where we find that DHS performs significantly better than GenoCanyon and DIVAN in a majority of comparisons; however, there is a substantial number of comparisons (22 out of 58) where either GenoCanyon or DIVAN outperforms DHS. Figure 7 further illustrates these

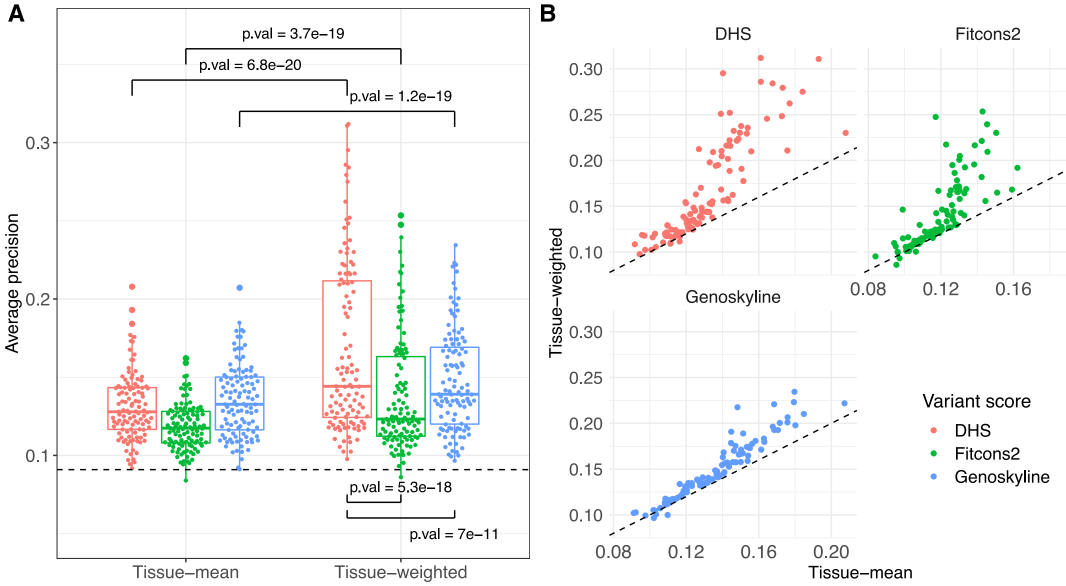

Figure 4. Disease-specific tissue weights improve variant prioritization
Performance of three tissue-specific variant scores (DHS, Fitcons2, GenoSkyline) is used to prioritize non-coding disease-associated variants for disease terms using two approaches: tissue-mean (i.e., disease agnostic, baseline) on the left side and tissue-weighted (i.e., disease specific) on the right side. *p* values were calculated using a Wilcoxon signed-rank test (A). Scatterplot of tissue-mean vs. tissue-weighted performance (average precision) for each tissue-specific score; dashed line denotes the diagonal (B).

comparisons. In Figure 7A, we show the performance across disease terms, grouped by the best-performing method. We see that tissue-weighted DHS outperforms DIVAN and GenoCanyon substantially on multiple sclerosis (MeSH: D009103), psoriasis (MeSH: D011565), and inflammatory bowel disease (MeSH: D015212); DIVAN outperforms GenoCanyon and DHS on arthritis, rheumatoid (MeSH: D001172) and heart failure

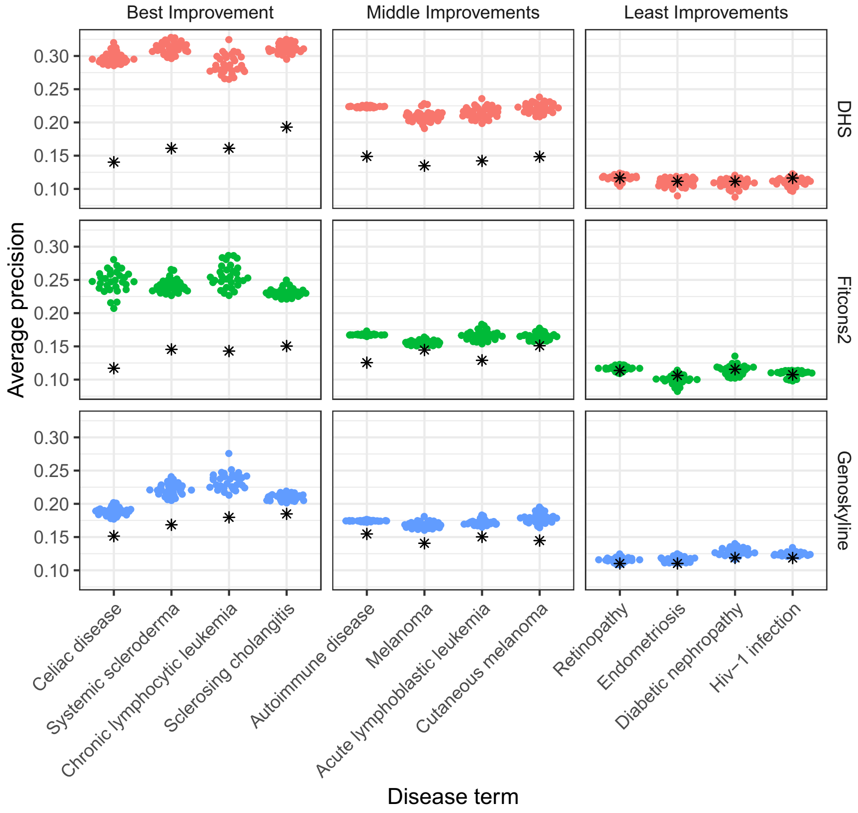

Figure 5. Improvement through disease-specific tissue weights is consistent across scores but varies with disease term
Shown is the performance of tissue-weighted variant scores (colored points) vs. tissue-mean (black asterisks) as a baseline, for three tissue scores (rows) and four diseases, stratified by improvement observed: best improvement for the first column, moderate improvement for the middle column, and least improvement for the right column. The x axes denote disease terms and the y axis average precision. Different points for tissue-weighted scores represent different data splits in the nested cross-validation procedure.

Table 2. DHS outperforms other tissue-specific scores

Score/method	By disease term			Aggregated		
	Wins	Losses	Ties	Wins	Losses	Ties
DHS	180	22	20	2	0	0
Genoskyline	96	94	32	1	1	0
Fitcons2	19	179	24	0	2	0

Wins, losses, and ties refer to significantly better (or worse, or tied) performance across all possible score pairings (see materials and methods). Columns 2-4 summarize separate comparisons for each disease term (for each row, there are two other methods and 111 terms, i.e., 222 comparisons), while the last three columns represent the results of comparisons aggregated over disease terms. Average precision was used as the performance metric, and the Wilcoxon signed-rank test was used to determine wins and losses (p values equal or larger than 0.05 are reported as ties).

(MeSH: D006333); and GenoCanyon outperforms DHS and DIVAN on stroke (MeSH: D020521) and Alzheimer disease (MeSH: D000544). In Figures 7B–7D, we directly summarize comparison results; we observe that the DHS tissue-weighted score often has an advantage in terms where prioritization efforts are overall more successful (top right quadrants). Finding overall good performance for our approach, we next more closely examined the disease-specific tissue aggregation weights we derive with our approach.

Disease-specific tissue weights reflect biomedical relevance

In addition to prioritizing SNPs, we can interpret the disease-specific tissue weights that our model learns in the context of disease mechanisms. Specifically, large tissue weights implicate tissues with a prominent role in associating SNVs with a disease in our model; therefore, one may hypothesize that such tissues or cell types have a function in the etiology of that disease. To investigate this hypothesis, we analyzed tissue weights of the top-performing models we derived, where each model represents a different disease.

The results are summarized in Table 5; they include the two top-performing models, systemic scleroderma (rank 1) and sclerosing cholangitis (rank 2). In order to report a diverse range of diseases, we next excluded any diseases that are descendants of immune system disease (EFO: 0000540) or lymphoma (EFO: 0000574). From the remaining diseases, we identify the next three highest-ranked models: colorectal adenoma (rank 15), atrial fibrillation (rank 20), and cutaneous melanoma (rank 21). For each disease, we list the five tissues with the largest tissue weights and their tissue group.

The tissues we associate with disease, overall, appear reasonable and generally are in line with existing knowledge about disease mechanisms. Systemic scleroderma is an autoimmune disorder that can affect skin and internal organs. We find that GM12878 lymphoblastoid cells (a type of B cell) are among the highest-weighted tissues, as were other types of B cells (primary B cells and B cell lymphoma, respectively). This is in line with previous studies that have shown that B cells play a role in system scleroderma.[28,29] Sclerosing cholangitis is an inflammatory condition that leads to scarring and narrowing of the bile ducts.[30] We highlight various inflammation-related types of blood cells, such as T cells and monocytes, which were previously suggested to play a role in the disease.[31] Colorectal adenoma is a benign tumor that develops in the lining of the colon or rectum. Our model identified rectal mucosa and stomach mucosa as the most-highly weighted tissues, and the function of rectal mucosa in colorectal cancer has been previously studied.[32] While the direct relationship between other gastrointestinal tissues and the development of colorectal adenoma has not been established, the association between gastrointestinal microbiome and colorectal adenomas has been discovered.[33] Regarding atrial fibrillation, our approach highlights fetal heart and lung tissues. In addition, we identified skeletal muscle cells. In the case of cutaneous melanoma, a type of skin cancer, our approach emphasizes foreskin melanocyte cells and a specific type of T cell. Apart from these, we highlight cervical carcinoma cell lines and endothelial primary cells.

Overall, we conclude that the tissue weights we derive carry biomedically meaningful information and are able to highlight tissue contexts that may play a role in disease etiology. To further explore this finding, we used a resource of the epimap consortium,[18] where disease-tissue associations are reported that derived differently from the ones we obtained in two key ways: first, epimap uses their enhancer definitions based on a much larger set of genome annotations. Second, epimap's enrichment test contrasts disease-associated SNP enrichment in a specific tissue's enhancer set compared to all enhancers, whereas our method effectively compares open chromatin harboring disease-associated SNPs vs. control SNPs tissue by tissue. Nevertheless, the results are summarized in Table S7, and we find that out of the 25 tissues we associate with disease terms, 14 have an estimated false discovery rate of less than 4% in the epimap analysis as well. Notably, a ground truth for these association is generally unknown, but we interpret the overlap in associations as encouraging, while complementary associations are expected, given the differences in methodology. Based on this overall finding of meaningful disease-tissue associations, we next further explored the use of tissue weights in disease characterization.

Disease-term similarity based on DHS tissue-weighted modeling reveals meaningful groups

Disease-specific tissue weights for aggregating DHS scores, which are learned by our approach, can highlight tissues

Table 3. DHS outperforms organism-level variant scores

	By disease term			Aggregated		
Score/method	Wins	Losses	Ties	Wins	Losses	Ties
DHS	474	44	37	5	0	0
GenoCanyon	314	198	43	4	1	0
LINSIGHT	298	230	27	1	2	2
GWAVA	233	289	33	1	2	2
eigen	223	299	33	1	2	2
CADD	28	510	17	0	5	0

Wins, losses, and ties refer to significantly better (or worse, or tied) performance across all possible score pairings (see materials and methods). Columns 2-4 summarize separate comparisons for each disease term (for each row, there are two other methods and 111 terms, i.e., 555 comparisons), while the last three columns represent the results of comparisons aggregated over terms. Average precision was used as the performance metric, and the Wilcoxon signed-rank test was used to determine wins and losses (p values equal or larger than 0.05 were reported as ties).

and cell types with a role in the disease (see previous section). Therefore, we derived and explored a measure for disease similarity based on these weights.

Disease similarities based on disease-specific tissue weights for non-coding variant prioritization

In our DHS tissue-weighted approach, for each disease term, DNA accessibility across the same set of tissue and cell-type contexts is used to predict whether a certain SNV is disease associated or not. This results in disease-specific tissue aggregation weights (that is, coefficients in our logistic regression model) $\{\beta^{(i)} \in \mathcal{R}^d\}_{i=1}^n$, where i is indexing disease terms, n is the number of disease terms studied, and d denotes the number of tissues/cell types with DHS scores. For our similarity measure between two diseases, say i and j, we then use a version of the Pearson correlation between $\beta^{(i)}$ and $\beta^{(j)}$ that takes uncertainty in the estimated aggregation weights into account (see materials and methods). That is, if an overlapping set of tissues/cell types drive the prioritization of SNVs for two diseases, then the similarity is high; if different tissues are used, then the similarity is low.

Using this approach, we calculated disease similarities for the 111 disease terms we study. Resulting similarities are visualized in Figure 8, where we show a similarity-based two-dimensional uniform manifold approximation and projection (UMAP) of disease terms. We observe that disease terms segregate into separate groups, with a coarse grouping between immune-related diseases (bottom left inlay, black) and others (bottom left inlay, gray). A higher-resolution group structure was obtained by subclustering, where we grouped disease terms into seven groups (Figure 8). Clusters names are based on EFO disease terms that include a large amount of cluster members as child terms (see materials and methods and Figures S10–S16); Table 6 lists disease terms per cluster. In addition to the clear separation of immune-related diseases from others, we also find a very homogeneous group consisting of mental and behavioral disorders, containing terms like

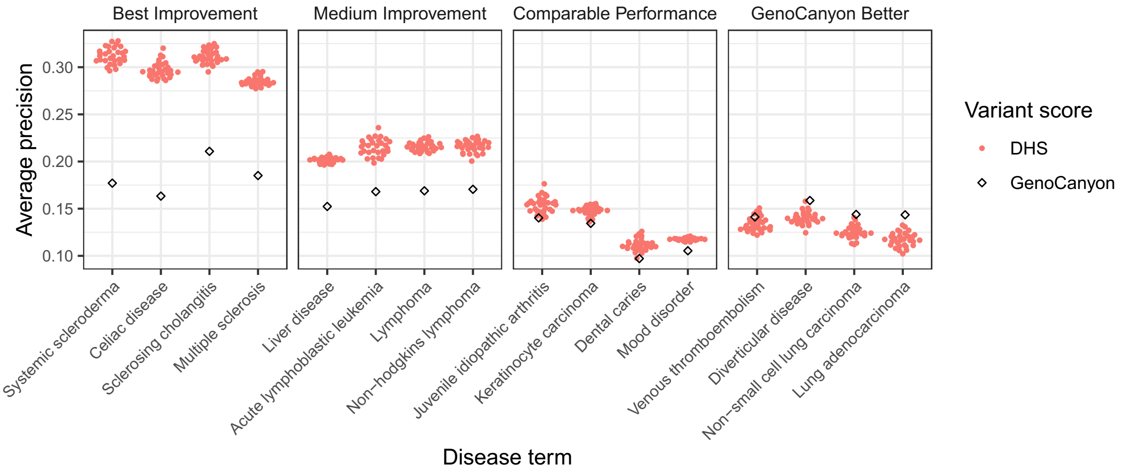

Figure 6. DHS disease-specific tissue weights improve variant prioritization compared with organism-level scores
For four strata (best improvement, middle improvement, comparable performance, and worse performance) we selected four disease terms and compared performance results. GenoCanyon (best organism-level score) performance is denoted in black and DHS tissue-weighted in red. Different performances of DHS tissue-weighted represent variation different data splits during nested cross-validation (see materials and methods).

Table 4. DHS tissue-weighted disease-specific scoring outperforms DIVAN

Score	Wins	Losses	Ties	Winning percentage (%)
DHS	34	22	2	61
GenoCanyon	26	31	1	46
DIVAN	25	32	1	44

Across 29 disease terms, this table summarizes all pairwise comparison for DHS tissue-weighted, GenoCanyon, and DIVAN using a specifically created test dataset. Wins, losses, and ties refer to significantly better (or worse, or tied) performance. Average precision was used as the performance metric, and the Wilcoxon signed-rank test was used to determine wins and losses (p values equal or larger than 0.05 were ties). Winning percentage = \#wins/(\#wins+\#losses).

schizophrenia (EFO: 0000692) and anxiety disorder (EFO: 0006788), and a group of skin cancers. The remaining three groups are more heterogeneous but with two of them containing several terms related to cardiovascular disease (EFO: 0000319) and digestive system disorders (EFO: 1000218), respectively. By design, similar tissues in each group drive SNP-disease associations, and we next examined which tissues play a role in each of the clusters.

In order to find group-specific tissues, we examined for each cluster the top five tissues that (1) contribute most to disease association and (2) are cluster specific (see materials and methods). The results are summarized in Figure 9; we note that both disease groups related to the immune system highlight blood tissues (such as E043: primary T helper cells from peripheral blood and E116: GM12878 lymphoblastoid cells; see Data SD23 for all names of standard epigenomes), with the group containing inflammatory bowel disease, Crohn disease (CD), and ulcerative colitis (UC) also containing rectum tissues (such as E101: rectal mucosa donor 29). Brain tissues contribute to disease associations for mental and behavioral disorders, skin tissues to skin cancer, and gastrointestinal/stomach tissue to the cluster with digestive system diseases. We also note that a clear association of specific tissues with a disease group correlates with the better classification performance of our model for SNP-disease association (Figure 9; for example, see the immune and immune/autoimmune clusters). We note, though, the corresponding tissue associations are not equally compelling for all clusters, as illustrated in the same figure. While the clusters we derive resemble broader disease groups, for each disease, a specific combination of tissues is used to derive whether a variant might be associated, and some tissues contribute to several clusters. For instance, one blood cell type (E116: GM12878 lymphoblastoid cells) contributes to both immune clusters but also to diseases in the digestive/cancer, heterogeneous, and skin cancer clusters. Another blood cell type (E043: primary T helper cells from peripheral blood) displays a similar pattern. Figure S9 shows the same heatmap as Figure 9 but for all tissues.

Overall, these results suggest that our modeling approach successfully identifies tissues with a role in disease etiology. Finally, we explore how our disease similarities relate to genetic similarities as measured by genetic correlation between diseases.

Model-based similarities are complementary to genetic correlation

Here, we compare the disease-disease similarities we derived (s_m) with genetic correlations from the GWAS Atlas (s_g), where genetic correlation measures shared genetic causes between two traits.[34] For 6,105 possible disease pairs of the 111 diseases terms we study, estimates of genetic correlation for 595 pairs were available from the GWAS Atlas (see materials and methods). Overall, for these 595 disease pairs, we observe only a weak (but statistically significant) correlation between model similarities and genetic correlations ($r = 0.32$, $p = 2.4E{-}15$), where the scatterplot is shown in Figure 10A.

We also see that most disease pairs are not annotated with substantial genetic correlations or with high model-based similarities (90% of disease pairs have $s_m < 0.25$ and $s_g < 0.2$). Therefore, we explored three different regimes: disease pairs where both similarity measures are high ($s_m \geq 0.25$ and $s_g \geq 0.20$), pairs with high genetic correlations and low model similarity ($s_m < 0.25$ and $s_g \geq 0.20$), and vice versa (quadrants indicated in Figure 10A, named quadrants B, C, and D). The top eight most extreme examples from each regime are summarized in Table 7. In the following, we discuss some examples in more detail. Specifically, we explore two immune system diseases for quadrant B, two mental or behavioral disorders for quadrant C, and one immune system disease and one mental or behavioral disorder for quadrant D. We note that the pairs we examine have no annotated parent-child relationships in the EFO.

UC (EFO: 0000729) and CD (EFO: 0000384) have both high genetic correlation ($s_g = 0.53$) and model similarity ($s_m = 0.84$) (see Figure 10A). This suggests that they share genetic causes and that the same tissues are informative for SNP-disease association. While shared genetic causes for UC and CD have been pointed out (e.g., Yang et al.[35]), our model for SNP-disease association allows us to explore relevant tissue contexts. In Figure 10B, we show a scatterplot of tissue weights for both diseases, where color indicates the importance of each tissue to model similarity (see materials and methods). We observe that open chromatin in blood (E116: GM12878 lymphoblastoid cells; E124: monocytes-CD14+ RO01746 primary cells; and E041: primary T helper cells PMA-I stimulated) and rectum (E102: rectal mucosa donor 31) is positively associated with SNP-disease association in both diseases; this is consistent with a previous study where blood cell

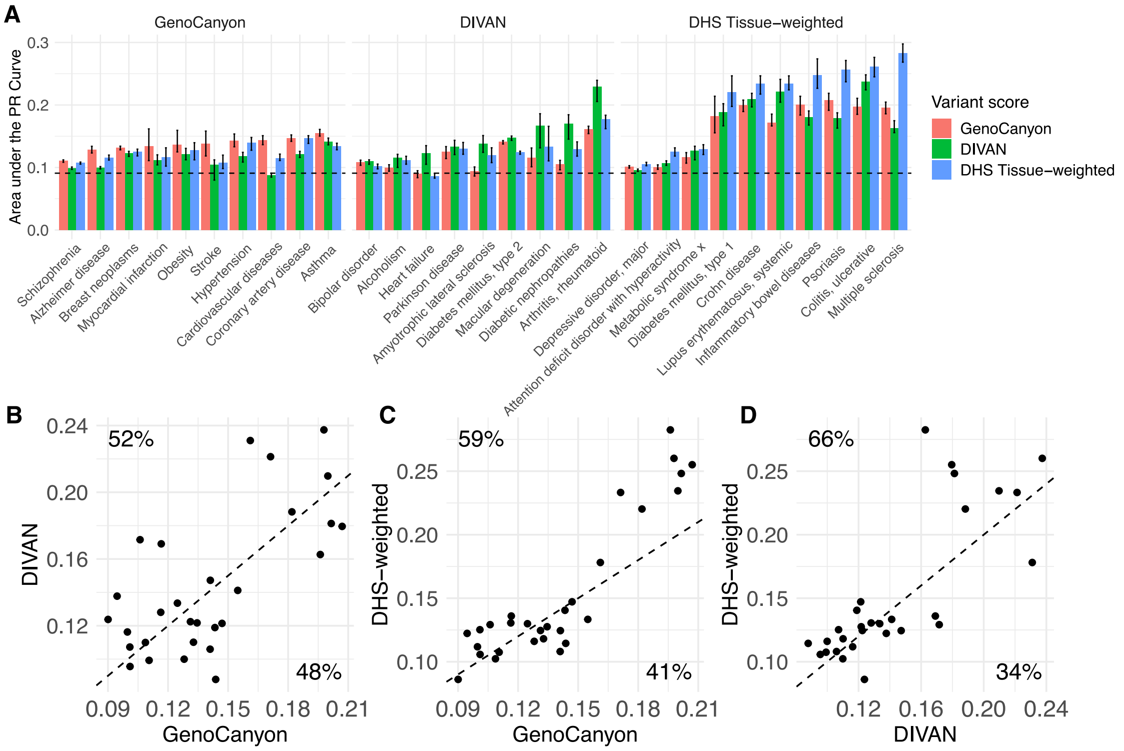

Figure 7. DHS tissue-weighted scoring outperforms DIVAN
Performance of DIVAN, GenoCanyon, and DHS tissue-weighted across a test set, with disease terms grouped by the best-performing method. Vertical stripe indicates the minimum and maximum performance of 30 bootstrap samples (A). Performance scatterplots of GenoCanyon vs. DIVAN performance (B), GenoCanyon vs. DHS-weighted (C), and DIVAN vs. DHS-weighted performance (D). Average precision was used for these plots; dashed lines denote equal performance. Percentages denote the fraction of points above and below the diagonal, respectively.

types were found to be relevant in many autoimmune diseases, including UC and CD.[36] In addition, symptoms or complications in the rectum are also observed in UC and CD.[37] Interestingly, open chromatin in gastrointestinal-intestine (E085: fetal intestine small) is negatively associated with SNP-disease association, along with other intestine tissues (E084: fetal intestine large and E109: small intestine, with the 61th and 86th smallest tissue weights, respectively, among 127 contexts). This indicates that the fetal intestine or small intestine might be less involved in UC and CD etiology compared to their juvenile and adult counterparts.

Autism spectrum disorder (ASD; EFO: 0003756) and anorexia nervosa (AN; EFO: 0004215) are an example where we observe a low genetic correlation ($s_g = -0.05$) and a moderate high model similarity ($s_m = 0.34$); a scatterplot of their tissue weights is shown in Figure 10C. Note that we did not choose one of the highlighted pairs in Table 7 for this quadrant because we already discussed an immune-system-related disease pair. We observe that both disease models give heart and brain tissue (E083: fetal heart and E081: fetal brain male) high tissue weights. This is consistent with the observation of brain abnormalities in ASD and AN.[38,39] While the presence of the fetal heart is less intuitive, we note that children with abnormal heart development are more likely to develop ASD, suggesting a connection between the disease and the fetal heart.[40] We also note that while the genetic correlation between ASD and AN is low, a link between the two diseases on the phenotypic level is being suggested[41,42]; the tissue context we identified could provide information about shared molecular aspects of disease etiology as well.

For obsessive-compulsive disorder (EFO: 0004242) and celiac disease (EFO: 0001060), we observe low model similarities ($s_m = -0.26$) and moderately high genetic correlation ($s_g = 0.36$); Figure 10D shows the scatterplot of tissue weights. Several studies have shown that nervous system disease and immune-related diseases have shared genetic backgrounds.[43,44] However, in contrast to the other two examples, there is little relation between tissue weights in these two diseases. Blood cell types are highlighted in celiac disease, while brain and fetal heart tissues are highlighted in obsessive-compulsive disorder. For celiac disease, the top six tissue contexts are blood cells, including different types of T cells (E041: primary T helper cells PMA-I stimulated; E043: primary T helper cells from peripheral blood; and E034: primary T cells from peripheral blood) and lymphoblasts (E116: GM12878 lymphoblastoid cells), which is consistent with findings that alterations in T cells and lymphoblasts can lead to celiac disease.[45,46]

Overall, these examples illustrate that the disease similarities we derive are complementary to genetic

Table 5. Top-ranked tissues for five diseases

Rank	ID	Tissue name	Group
**Systemic scleroderma**			
1	E116	GM12878 lymphoblastoid cells	blood
2	E032	primary B cells from peripheral blood	blood
3	E041	primary T helper cells PMA-I stimulated	blood
4	E123	K562 leukemia cells	blood
5	E030	primary neutrophils from peripheral blood	blood
**Sclerosing cholangitis**			
1	E116	GM12878 lymphoblastoid cells	blood
2	E061	foreskin melanocyte primary cells skin03	skin
3	E102	rectal mucosa donor 31	gi_rectum
4	E041	primary T helper cells PMA-I stimulated	blood
5	E029	primary monocytes from peripheral blood	blood
**Colorectal adenoma**			
1	E102	rectal mucosa donor 31	gi_rectum
2	E110	stomach mucosa	gi_stomach
3	E057	foreskin keratinocyte primary cells skin02	skin
4	E101	rectal mucosa donor 29	gi_rectum
5	E028	breast variant human mammary epithelial cells (vHMECs)	breast
**Atrial fibrillation**			
1	E083	fetal heart	heart
2	E108	skeletal muscle female	muscle
3	E107	skeletal muscle male	muscle
4	E088	fetal lung	lung
5	E120	HSMM skeletal muscle myoblast cells	muscle
**Cutaneous melanoma**			
1	E061	foreskin melanocyte primary cells skin03	skin
2	E059	foreskin melanocyte primary cells skin01	skin
3	E117	HeLa-S3 cervical carcinoma cell line	cervix
4	E041	primary T helper cells PMA-I stimulated	blood
5	E122	HUVEC umbilical vein endothelial primary cells	vascular

This shows the top-five tissues with the largest tissue weights in the corresponding model we derive, for five diseases. The first column is the tissue rank, the second the tissue's roadmap ID, the third the tissue name, the fourth the tissue group.

correlation. In addition, tissue contexts highlighted by our tissue weights allow for biomedical interpretations of observed similarities (i.e., which are the relevant tissue contexts) and can be used to generate molecular hypotheses about disease etiology.

In summary, our results show that disease-specific variant prioritization performs well for non-coding GWAS variants, compared with organism-level approaches. We also demonstrate that disease-specific tissue weights are biomedically meaningful and can be used to generate hypotheses about disease mechanism. Therefore, we believe that this type of variant characterization is a useful tool for researchers studying the molecular and genetic causes of disease.

Discussion

Most variant scores prioritize non-coding variants either at the level of the whole organism (e.g., CADD,[8]

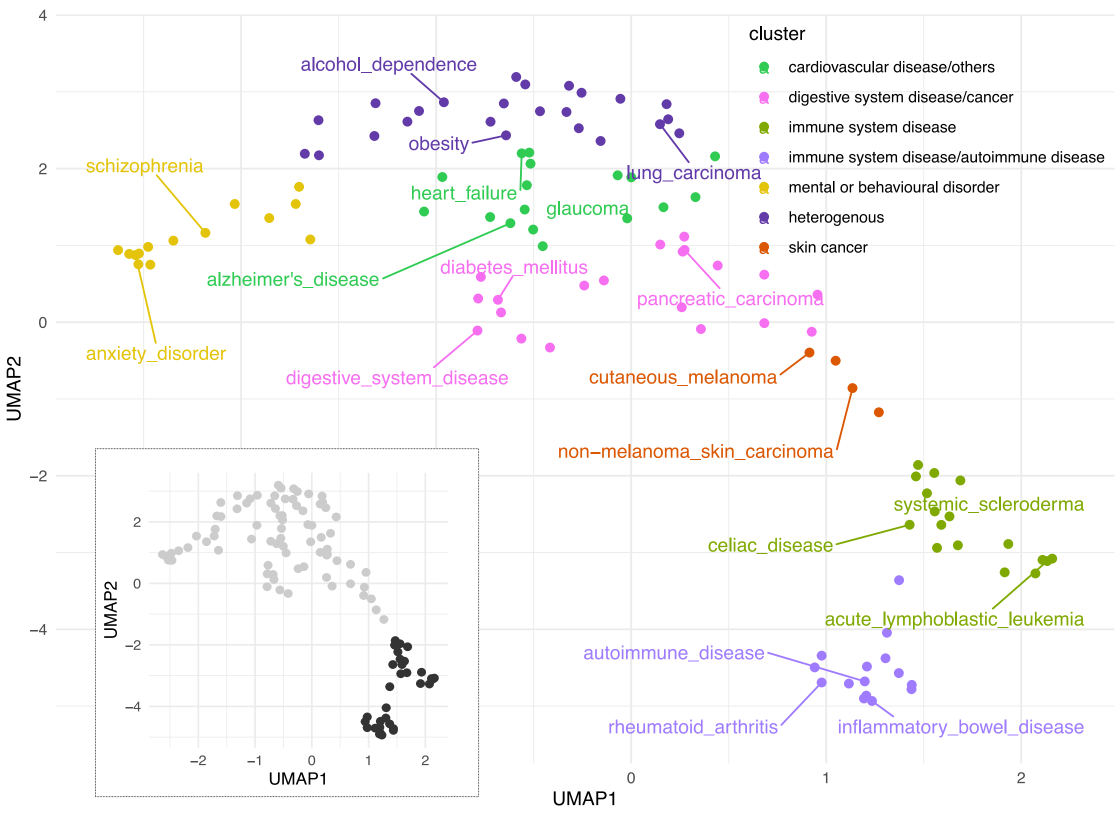

Figure 8. Similarity-based two-dimensional projection visualizes 111 diseases
Two dominant disease groups emerge in this visualization (immune system-related disease terms [black] and others [gray], in the inlay). Hierarchical clustering was used to group diseases into seven clusters, with colors indicating broad disease types (see Table 6 for details).

GenoCanyon[26]) or provide tissue-specific scores (e.g., GenoSkyline,[11] Fitcons2[12]). Here, we present a straightforward strategy to combine tissue-specific variant scores in a disease-specific manner. We show that for common genetic variants in the GWAS Catalog,[1] our approach leads to a better performance than organism-level or tissue-specific scores (see Figure 6). Pre-computed disease-specific prioritization scores are available at https://doi.org/10.7910/DVN/AUAJ7K. Comparing different variant prioritization methods, we note that we use area under the precision-recall curve as an evaluation metric and that the performance of all methods is modest. We believe that is because our analysis (1) focuses explicitly on non-coding variants, (2) stratifies SNVs by disease phenotype, and (3) utilizes unbiased matching of control SNVs (SNPsnap matching; see control variants). Each of these points affects the SNV sets we use for our analysis and therefore the performance metrics we report. For transparency, we provide all disease-associated variants we use (with matched negatives) in Data SD1. As a more general point, we also note that associations reported in the GWAS Catalog contain causal as well as non-causal SNPs, which will also contribute to sub-optimal performance measures of all the variant scores we assess.

We included a comparison with the DIVAN method in our evaluation, which also includes comparing GenoCanyon with DIVAN. Part of this comparison is analogous to results reported in Chen et al.[22]; however, the performances we observed do not agree perfectly, as detailed in Data SD15. Broadly, looking at overlapping/matching disease terms, our results appear more favorable for GenoCanyon. These differences are likely due to different test sets used in the two evaluations (i.e., the GWAS Catalog [this study] vs. Genome-Wide Repository of Associations Between SNPs and Phenotypes).

We also note that there is other research associating variants with disease terms in a similar setting, notably PINES[23] and LSMM.[47] We did not compare directly with PINES because no pre-computed scores are available; also, we note that while the performance reported in this publication in terms of the AUROC is higher than our results, a less stringent un-matched test set of random/control variants was used in these analyses. For LSMM, we note that we leverage variants associated with EFO disease terms across studies, while LSMM uses summary statistics on a per-study basis. Using aggregate data from different studies allows our approach to consider parent-child relationships of the experimental factor ontology using variant aggregation (see disease-specific non-coding variant prioritization with organism-level variant scores is only moderately successful).

We demonstrate that our approach can be used to calculate similarities between disease terms (see disease similarities based on disease-specific tissue weights for non-coding variant prioritization). Since this similarity measure is

Table 6. Disease groups based on DHS tissue-weights

■ heterogenous

- adolescent idiopathic scoliosis
- age-related macular degeneration
- ■ alcohol dependence
- amyotrophic lateral sclerosis
- chronic obstructive pulmonary disease
- ■ dental caries
- diabetic nephropathy
- ■ drug dependence
- □ endometriosis
- epilepsy
- gout
- hiv infection
- hiv-1 infection
- ■ lung adenocarcinoma
- ■ lung carcinoma
- neuropathy
- ■ non-alcoholic fatty liver disease
- ■ non-small cell lung carcinoma
- obesity
- ■ periodontitis
- peripheral neuropathy
- scoliosis
- ■ ■ squamous cell lung carcinoma
- ■ venous thromboembolism

■ digest/cancer

- ■ ■ autoimmune thyroid disease
- ■ breast carcinoma
- ■ cancer
- ■ cardiovascular disease
- ■ colorectal adenoma
- ■ ■ colorectal cancer
- ■ coronary artery disease
- ■ diabetes mellitus
- ■ ■ digestive system carcinoma
- ■ digestive system disease
- female reproductive system disease
- ■ hypertension
- ■ ■ multiple myeloma
- ■ neurotic disorder
- ■ ■ pancreatic carcinoma
- ■ prostate carcinoma
- respiratory system disease
- ■ ■ squamous cell carcinoma.
- ■ ■ ■ type i diabetes mellitus
- ■ type ii diabetes mellitus

■ immune

- ■ ■ acute lymphoblastic leukemia
- adult onset asthma
- ■ allergic rhinitis
- ■ allergy
- atopic asthma
- ■ ■ celiac disease
- childhood onset asthma
- ■ ■ chronic lymphocytic leukemia
- ■ cirrhosis of liver
- hypothyroidism
- ■ ■ juvenile idiopathic arthritis
- ■ ■ lymphoid leukemia
- ■ lymphoma
- ■ ■ neoplasm of mature b-cells
- ■ non-hodgkins lymphoma
- ■ ■ systemic lupus erythematosus
- ■ ■ systemic scleroderma

■ cardiovascular/others

- ■ alzheimer's disease
- ■ atherosclerosis
- ■ atrial fibrillation
- ■ cardiac arrhythmia
- chronic kidney disease
- ■ diverticular disease
- glaucoma
- ■ heart failure
- metabolic syndrome
- ■ migraine disorder
- osteoarthritis
- ■ ovarian carcinoma
- parkinson's disease
- ■ peripheral arterial disease
- retinopathy
- ■ stroke
- uterine fibroid

■ immune/autoimmune

- ■ ■ ankylosing spondylitis
- asthma
- ■ ■ autoimmune disease
- ■ ■ ■ crohn's disease
- ■ hypersensitivity reaction disease
- ■ immune system disease
- ■ ■ ■ inflammatory bowel disease
- kidney disease
- ■ liver disease
- ■ ■ multiple sclerosis
- ■ psoriasis
- ■ ■ rheumatoid arthritis
- ■ sclerosing cholangitis
- skin disease
- ■ ■ ■ ulcerative colitis

■ mental

- ■ anorexia nervosa
- ■ anxiety disorder
- ■ attention deficit hyperactivity disorder
- ■ autism spectrum disorder
- ■ bipolar disorder
- ■ eating disorder
- ■ mental or behavioural disorder
- ■ mood disorder
- movement disorder
- ■ obsessive-compulsive disorder
- ■ psychosis
- ■ schizophrenia
- ■ tourette syndrome
- ■ unipolar depression

■ skin cancer

- ■ ■ cutaneous melanoma
- ■ ■ keratinocyte carcinoma
- ■ ■ melanoma
- ■ ■ non-melanoma skin carcinoma

legend

- ■ digestive system disease
- ■ immune system disease
- ■ autoimmune disease
- ■ cardiovascular
- ■ mental or behavioural disorder
- ■ skin cancer
- ■ cancer

For each disease group, disease terms are shown. The colored squares denote the disease groups in the EFO ontology.

derived from non-coding SNVs associated with disease, one could expect it is largely congruent with genetic correlation between disease traits. However, that is not the case (see Figure 10), most likely because we focus on a small subset of disease-associated SNVs reported in the GWAS Catalog. For example, obsessive-compulsive disorder and celiac disease have a high genetic correlation ($s_g = 0.36$) but do not share non-coding SNPs in the GWAS Catalog (and low model similarity, $s_m = -0.26$); on the other hand, ASD and AN have a low genetic correlation ($s_g = -0.05$) but share a number of significant SNPs in the GWAS Catalog (and relative high model similarity, $s_m = 0.34$). In addition, the interpretation of model similarity between disease terms is different from genetic correlation; high model similarity implies that disease-associated SNVs reside in DNA-accessible regions in an overlapping set of tissues, but the identity of individual SNVs (and whether they overlap) is inconsequential. For example, asthma and rheumatoid arthritis (RA) have only 15 shared SNPs (out of 732 and 1,283 SNPs in RA and asthma, respectively) but exhibit high model similarity ($s_m = 0.53$). This shows that model similarity between two diseases can involve similar tissues even if they do not share a genetic background. Further on, we note that estimates of genetic correlation also may depend on the study used. For example, systemic lupus erythematosus (SLE) has a negative genetic correlation ($s_g = -0.47$) with RA (and other inflammatory diseases) when using the SLE summary statistics from Julia et al.[48] (as retrieved from the GWAS Atlas[34]), whereas another study (Lu et al.[49]) found SLE to have a positive genetic correlation ($s_g = 0.41$) with RA when using the SLE summary statistics from Bentham et al.[50]

We note that in our analyses, we used the EFO ontology to aggregate variants annotated in the NIH/EBI GWAS Catalog. That is, for each disease term, directly annotated variants were used, and, in addition, variants annotated to descendant terms in the ontology were also included. This approach allowed us to compile a more exhaustive set of variants per term. However, some amount of caution should be exercised when using disease models with more general terms, such as "cardiovascular disease," for example, as they may encompass heterogeneous diseases.

Our approach is expected to improve as more variants are associated with disease and as disease associations get more

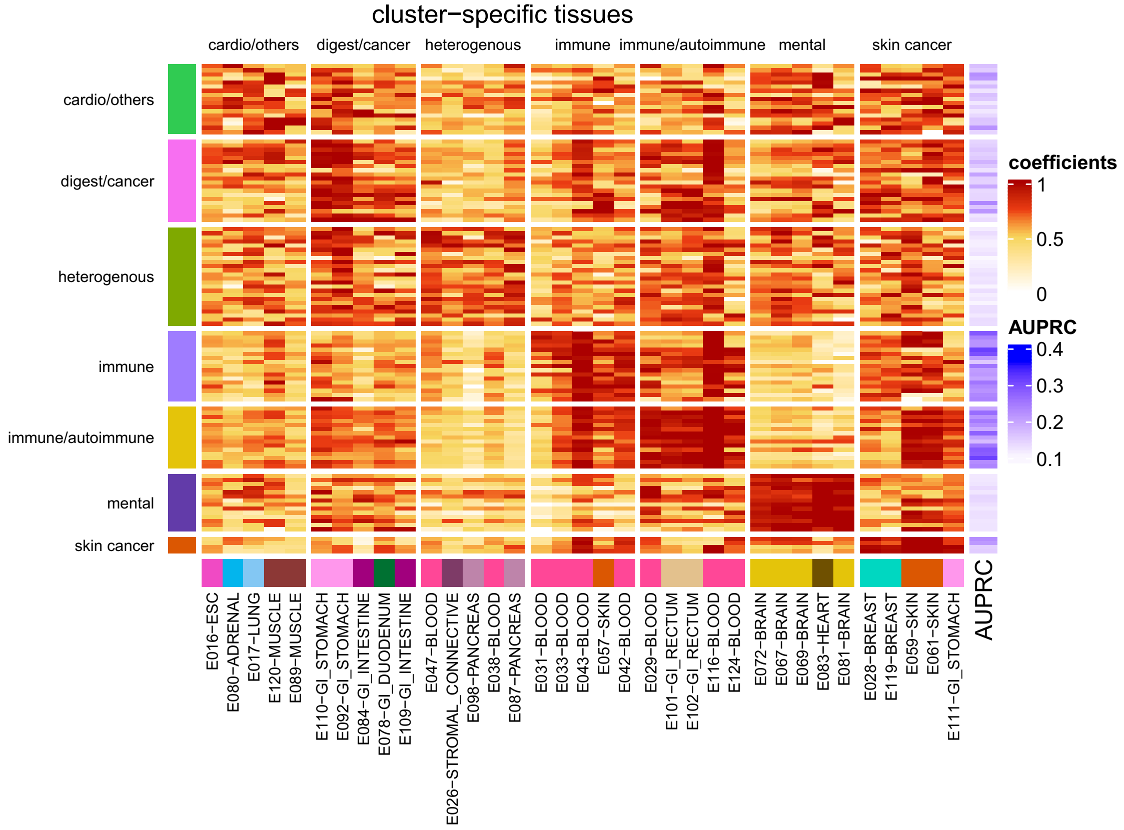

Figure 9. Heatmap of top-five tissue-weights for 111 diseases
Regularized model coefficients (i.e., tissue weights) of five disease-cluster-specific tissues (columns) are shown for 111 diseases (rows). Coefficients are scaled by disease, and rows are grouped into sets of cluster-specific tissues (see materials and methods section). Bottom annotation shows tissue names of cluster-specific tissues (names are shown in the format of "tissue name" – "tissue group"; annotation on the left side shows disease cluster, and annotation on the right side shows model performance in terms of AUPRC.

refined. In addition, increasing amounts of epigenomics data, such as epimap[18] and ENCODE5,[6] could be incorporated, and they have the potential to improve the disease associations we learn. Further on, recent studies have shown benefits of including functional annotations in rare variant association analyses.[51,52] In this context, our DHS-derived scores can be included as data-driven disease-specific combinations of functional genome annotations and included in this type of approach. It is future work to assess if this could be an approach to bring GWAS data and functional annotations to bear in rare variant analysis.

In summary, we have provided a straightforward method to leverage tissue-specific variant scores for disease-specific variant prioritization. We show that this approach performs well compared with current methods, and we show that the resulting association models are interpretable and lead to useful characterization of disease terms. Overall, our contributions are useful for the following two reasons: conceptually, because they highlight the value of disease-specific variant prioritization, and in addition, because we provide pre-computed prioritization scores for 111 disease terms that researchers can use in practice to interpret their variant data.

Materials and methods

Data sources and processing

Disease-associated variants

Disease-associated non-coding SNVs were retrieved from the NHGRI-EBI Catalog of human GWAS database (GWAS Catalog, v.2020-12-02, downloaded from https://www.ebi.ac.uk/gwas/docs/file-downloads). These data contained 122,396 unique non-coding SNPs spanning 2,782 phenotypes, where non-coding was defined as variants not overlapping with a protein-coding sequence (GENCODEv.36); we also excluded variants annotated as protein-coding sequence variants (e.g., missense variants, frameshift variants) as an SNP's "functional class" in the GWAS Catalog. Further, variants in the GWAS Catalog are annotated with phenotypes using the EFO (https://www.ebi.ac.uk/efo).[53] We focused on variants with phenotype terms annotated in the disease domain of the EFO (i.e., all terms/traits/phenotypes we consider are descendants of the term "disease" [EFO: 0000408, EFO v.3.24.0, accessed November 17, 2020]). Further on, SNPs in the human leukocyte antigen region and SNPs with a minor allele frequency (MAF) less than 1% in the European population as reported by the International Genome Sample Resource were excluded (as they cannot be matched to control SNPs with the SNPsnap approach; see below). Out of 31,103 SNVs, a total of 5,225 SNVs were removed. Finally, in our analyses, we restricted

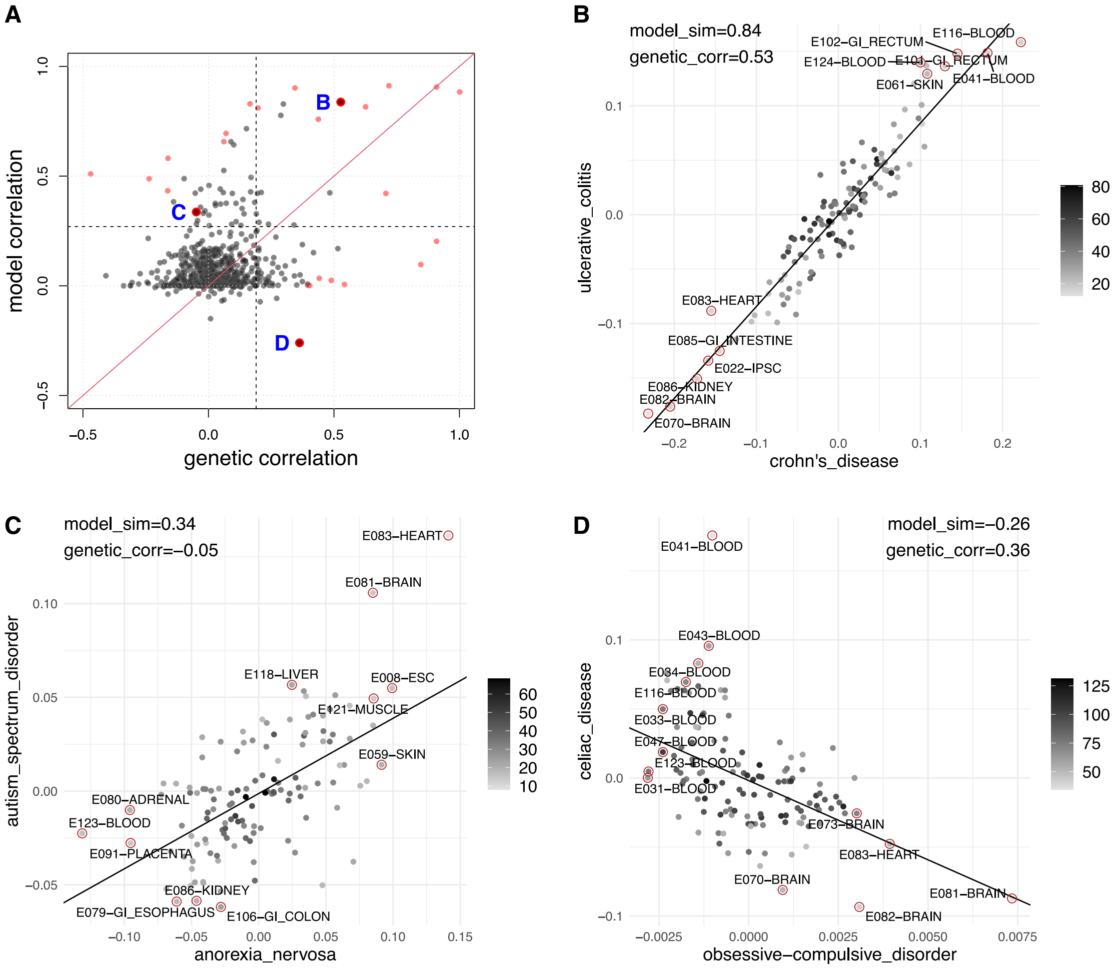

Figure 10. Genetic correlation and model similarity

(A) Genetic correlation vs. model similarity for 595 disease pairs. Each point is a disease pair, where the x axis denotes the genetic correlation and y axis is the disease model similarity. For three quadrants, we highlight disease pairs, denoted by B, C, and D.

(B–D) Scatterplot of tissue coefficients in three example disease pairs, where (B) shows Crohn disease vs. inflammatory bowel disease, (C) shows anorexia nervosa vs. autism spectrum disorder, and (D) shows celiac disease vs. obsessive-compulsive disorder. Lines denote a weighted linear regression line underlying our disease similarities. Color codes for the weight for each tissue when conducting weighted regression analysis.

ourselves to phenotypes with at least 100 annotated non-coding SNPs. Data SD1 and SD2 contain the 111 phenotypes and 77,028 phenotype-associated SNPs we used in this study. We also grouped SNPs in LD blocks (SNPsnap, $r^2 \geq 0.5$) and identify SNPs with the minimum p value per block ("representative SNP"); we provide this information, which we use in some of the analyses described below, in Data SD2.

Control variants

For each disease-associated SNP, we generated matched control non-coding variants with MAF $\geq$ 1% using four different strategies, where the non-coding is again defined discussed above (disease-associated variants). The four strategies are as follows:

- Random: for each disease-associated SNP, we selected ten SNPs from common non-coding variants in 1000G (European, EUR) at random (i.e., equal probability for all SNPs) as controls.
- Transcription start site (TSS) matching: we processed common non-coding SNVs and selected a subset of these variants as controls, where the distribution of distances to the nearest protein-coding gene's TSS are matched between control set and disease-associated SNPs (similar to GWAVA[27]). Specifically, we sorted all common non-coding SNPs by the distance to the nearest TSS and divided them into 50 bins, where each bin contains the same number of SNVs. Then, for each disease-associated SNP, we randomly selected ten control SNPs from the bin containing the disease-associated SNP's distance to the nearest gene.
- SNPsnap matching: using SNPsnap,[25] we matched control SNPs to disease-associated variants in terms of MAF, gene density (distance cutoff ld0.8), distance to the nearest gene TSS, and number of SNPs in LD. Our parameters for maximum allowable deviation were 5%, 50%, 20%, and 50%, respectively. We randomly selected ten control SNPs per disease-associated SNP form SNPsnap's results, and we ensured that there were no duplicated control SNPs for different disease-associated SNPs. If there were less than 10 control SNPs returned by SNPsnap, then we kept all of the control SNPs. If no control SNPs were matched, then we

Table 7. Example disease pairs of genetic correlation and model similarities

Disease 1	Disease 2	s_g	s_m	Quadrant
Inflammatory bowel disease	ulcerative colitis	1.00	0.88	B
Diabetes mellitus	type 2 diabetes mellitus	0.91	0.91	B
Crohn disease	inflammatory bowel disease	0.72	0.91	B
Sclerosing cholangitis	ulcerative colitis	0.63	0.82	B
Crohn disease	ulcerative colitis	0.53	0.84	B
Ankylosing spondylitis	sclerosing cholangitis	0.35	0.90	B
Inflammatory bowel disease	sclerosing cholangitis	0.44	0.76	B
Bipolar disorder	schizophrenia	0.71	0.42	B
Rheumatoid arthritis	systemic lupus erythematosus	−0.47	0.51	C
Celiac disease	systemic lupus erythematosus	−0.16	0.58	C
Sclerosing cholangitis	systemic lupus erythematosus	−0.24	0.49	C
Crohn disease	sclerosing cholangitis	0.17	0.83	C
Rheumatoid arthritis	sclerosing cholangitis	0.07	0.69	C
Crohn disease	rheumatoid arthritis	0.06	0.66	C
Systemic lupus erythematosus	ulcerative colitis	−0.16	0.43	C
Crohn disease	systemic lupus erythematosus	−0.10	0.49	C
Type 1 diabetes mellitus	type 2 diabetes mellitus	0.85	0.10	D
Diabetes mellitus	type 1 diabetes mellitus	0.91	0.20	D
Celiac disease	obsessive-compulsive disorder	0.36	−0.26	D
Diabetes mellitus	obesity	0.54	0.01	D
Obesity	osteoarthritis	0.49	0.02	D
Attention-deficit hyperactivity disorder	obesity	0.44	0.03	D
Attention-deficit hyperactivity disorder	osteoarthritis	0.40	0.00	D
Obesity	type 1 diabetes mellitus	0.40	0.00	D

This table shows the genetic correlation and model similarity for some disease pairs as we selected. For quadrants B, C, and D, we picked 8 disease pairs, where $s_g + s_m$, $s_g - s_m$, and $s_m - s_g$ are the highest, respectively. s_g, genetic correlation; s_m, model similarity.

removed the disease-associated SNVs (a total of 311 SNVs) from our analyses.

- SNPsnap-TSS matching: this is essentially the same as in SNPsnap matching but controlling only for the distance to the nearest genes (maximum allowable deviation: 20%); for three other attributes, "maximum allowable deviation" is set to 10,000%. We note that in both SNPsnap matching and SNPsnap-TSS matching, the distance is measured by the distance to the nearest gene, whereas for TSS matching, only protein-coding genes are considered.

In all four matching strategies, we excluded variants annotated in the GWAS Catalog as control SNPs. One control variant can only be matched to one disease-associated SNV. In our research, we chose SNPsnap matching for our main results, but we have compared the different performances of organism-level scores using the four different matching strategies (see supplemental methods and Figures S1 and S2). We also provided the four sets of control variants in Data SD3.

Additional data sources and variant scores

We used pre-computed SNP annotations from the following sources: CADD v.1.3 (http://krishna.gs.washington.edu/download/CADD/v1.3/1000G_phase3.tsv.gz); EigenPC v.1.1 (https://xioniti01.u.hpc.mssm.edu/v1.1); Fitcons2 (http://compgen.cshl.edu/fitCons2/hg19); GenoCanyon (http://genocanyon.med.yale.edu/GenoCanyon_Downloads.html); GenoSkylinePlus (http://genocanyon.med.yale.edu/GenoSkylineFiles/GenoSkylinePlus/GenoSkylinePlus_bed.tar.gz); GWAVA v.1.0 (ftp://ftp.sanger.ac.uk/pub/resources/software/gwava/v1.0/VEP_plugin/gwava_scores.bed.gz); LINSIGHT (http://compgen.cshl.edu/%7Eyihuang/tracks/LINSIGHT.bw); and DIVAN (https://sites.google.com/site/emorydivan). DHS accessibility: we downloaded Avocado-imputed[54] DHS signals for 127 ENCODE biological contexts (tissues/cell types) from https://noble.gs.washington.edu/proj/avocado/data/avocado_full/DNase/.

Tissue-weighted variant prioritization based on DNase1 hypersensitivity

A penalized logistic regression model for context-weighted score averaging

For predicting SNP's associations with a disease term, we consider SNPs as observations, and each is described as a vector $x \in R^d$ of variant scores in d tissues/contexts; we arrange vectors $\{x_i\}_{i=1}^n$ for n observations in a matrix $X \in R^{n \times d}$ together with a vector y of n binary entries, encoding for each SNP its

association with a specific disease term (no = 0/yes = 1). In addition, we denote the average score (across contexts) for an SNP i by $\bar{x}^i$, which is also a basline score because it aggregates across contexts.

We use a logistic regression model of the form

$$\log\frac{p_i}{1-p_i} = \alpha\bar{x}^i + \beta' x^i \ s.t.\ \alpha \geq 0, \quad \text{(Equation 1)}$$

where $\alpha_0 \in R$, $\alpha \in R_+$, and $\beta \in R^d$ are regression coefficients and p_i is the probability that SNP i is associated with a disease that is studied. We fit a regularized version of the negative log likelihood

$$\text{argmin}_{\alpha,\alpha_0,\beta} - \frac{1}{2}\sum_{i=1}^{n}\left[\log(1-p_1) + y_i \log\frac{p_i}{1-p_i}\right] + \frac{\lambda||\beta||_2^2}{2}, \quad \text{(Equation 2)}$$

where the dependence on $\{\alpha, \alpha_0, \beta\}$ of the first term is through Equation 1. For large regularization parameters λ, this will yield small $\beta \to 0$ and recover the baseline $(\bar{x})$ of unweighted averaging of context scores (scaled by a non-negative factor α). We implemented this approach using the R package glmnet (v.2.0-18[55]) and determined the regularization parameter via 5-fold cross-validation (cv.glmnet function) through maximizing the area under the (cross-validated) ROC curve. In the nested 5-fold cross-validation, we used the inner loop to select the regularization parameter λ and the selected λ to train and test the model in the outer loop. Class weights were employed to balance skewed class sizes.

Disease similarities from context-weighted score averaging

Context-weighted score averaging, as described above, results in disease-specific coefficient vectors ($\{\beta^{(i)}\}$, with i indexing disease terms), together with bootstrap estimates for the standard deviation of each coefficient (that can be arranged in corresponding vectors $\{\gamma^{(i)}\}$). Specifically, we use 5-fold cross-validation repeated 10 times, yielding 50 coefficient vectors for each disease. We use their mean for our estimate of $\beta^{(i)}$ and their standard deviation as an estimate of $\gamma^{(i)}$. For a pair of diseases (d_i, d_j), we then define a disease similarity through the similarity of associated coefficient vectors $\beta^{(i)}$ and $\beta^{(j)}$, taking into account our estimates of coefficient variability. Specifically, we fit a weighted linear regression model (i.e., regressing vectors $\beta^{(i)}$ on $\beta^{(j)}$), with regression weights taking into account coefficient variability as follows:

$$w_k^{(i,j)} = \frac{1}{\sqrt{s_k^i s_k^j}} \quad \text{and} \quad s_k^\circ = \alpha\gamma_k^{(\circ)} + (1-\alpha)m \quad \text{for} \quad \circ \in \{i,j\}, \quad \text{(Equation 3)}$$

where we chose m to be the 25% quantile of all (estimated) standard deviations observed and $\alpha = 3/4$. Therefore, s_k^i and s_k^j are shrunken versions of the standard deviations for the regression coefficients of disease i and disease j in tissue/context k, respectively. Finally, for disease pairs with a positive coefficient from the weighted linear regression, we take the coefficient of determination (r^2) as a similarity measure; for disease pairs with a negative coefficient, we take $-r.^2$ We note that for constant regression weights $\{w^{(i,j)}\}$, this is equal to the Pearson correlation between the coefficient vectors we obtain from context-weighted score averaging (i.e., cor$(\beta^{(i)}, \beta^{(j)})$).

Variant prioritization performance

Tissue-weighted cross-validation performance

To measure the cross-validation performance of tissue-weighted scores, we use repeated cross-validation[56] to reduce the variance (due to the random partitioning of data into 5-fold). Here, we repeat fold cross-validation 30 times and record the performance of each repeat. We later use the mean performance of the 30 repeats as the performance of that method, and we also show the variance in figures, such as in Figure 5.

Comparing organism-level scores

For each disease, we have disease-associated and control SNVs and corresponding pre-computed organism-level scores. With this setup, we calculate performance metrics of interest (AUROC and average precision) and obtain disease-specific performance metrics for each scoring approach. To compare performances between organism-level scores on the same disease, we use performance measures computed on 30 bootstrap samples (each bootstrap sample randomly contains 90% of disease and control variants) and then employ the Wilcoxon signed-rank test to test to assess differences in performance. This yields p values as reported in Data SD4.

With respect to aggregating comparisons across diseases, we note that disease terms can (and do) share SNVs, so performance metrics in different terms are not necessarily independent. Also, disease terms can vary substantially in the number of annotated SNPs. We again use the Wilcoxon signed-rank test[57] on performance metrics (computed using all disease-associated and control SNVs for each disease term) to compare two organism-level variant score aggregates across diseases. This approach yields p values, as reported in Data SD5.

Comparing tissue-weighted scores

Tissue-weighted baseline scores (see above) are calculated in the same way as organism-level scores. For tissue-weighted scores with data-driven tissue-specific weighting (see above), we use cross-validated performance measure for each bootstrap sample and the same 30 bootstrap samples as when we compared between organism-level scores. And then, we use the same Wilcoxon signed-rank tests to measure the difference. For comparing scores aggregated across diseases, we again proceed analogous to organism-level scores and use a Wilcoxon signed-rank test on cross-validated disease-specific performance measures. Results are summarized in Data SD8 and SD9.

Comparing organism-level and tissue-weighted scores

For comparisons between organism-level and tissue-combined scores, we again use a bootstrap approach: for a specific disease term, we use the Wilcoxon signed-rank tests as discussed above to compare performance measures from organism-level scores with tissue-weighted scores. We note that this approach does not take into account (1) variability in the organism-level scores originating form variability of the data they are derived from or (2) the possibility that organism-level scores may have already used SNPs in their score derivation process, and we use them again for evaluation in their score derivation process. However, we do not expect these issues to substantially confound or results, and we note that incurred bias in our comparisons would expected to be in favor of organism-level scores. The results are summarized in Data SD6–SD11.

DIVAN performance assessment and comparison

To assess and compare our performance with DIVAN,[22] we generated a test set of SNPs from the GWAS Catalog that were (1) added after DIVAN had been published (i.e., after May 28, 2016), (2) not present in the database used to train DIVAN (association result browser: https://www.ncbi.nlm.nih.gov/projects/gapplus/sgap_plus.htm), (3) not within a 1 kb distance around SNPs used to train DIVAN, and (4) annotated to a disease phenotype addressed by DIVAN. Control SNPs were generated using SNPsnap matching, as described above. To be able to satisfy criterion (4), we mapped our disease terms (EFO terms) to disease terms used

by DIVAN (MeSH terms) using the EMBL-EBI Ontology Xref Service (https://www.ebi.ac.uk/spot/oxo/, retrieved on April 19, 2020) and were able to resolve 41 out of 45 terms (Data SD12). Of these, we keep terms with 20 or more disease-associated SNPs in the test set and 50 or more SNPs in a training set that we also constructed (see below), yielding 29 overall disease phenotypes that we used in our analysis. In order to fairly compare DIVAN with our logistic regression approach, we constructed a training set using disease-associated SNPs from the GWAS Catalog and the Phenotype-Genotype Integrator (https://www.ncbi.nlm.nih.gov/gap/phegeni),[58] excluding SNPs in the test dataset describe above or SNPs within 1 kb around test SNPs. Data SD13 summarizes test and training data used for this analysis. The results are summarized in Data SD14.

Performance assessment using chromosome holdout

To assess the performance of our DHS tissue-weighted score, we also used a chromosome hold-out strategy with test SNPs on different chromosomes from training data. Specifically, for each disease, we choose a set of chromosomes that contains approximately 20% SNVs with a 1/10 positive-to-negative ratio (the same as the cross-validation setting) as a test set. The selection of test chromosomes is performed for each disease term separately, as disease-associated SNPs differ. To automate the procedure, we deployed (binary) linear programming to pick out chromosomes in the test set for each disease. Specifically, for each disease term, we solve the optimization problem

$$\text{argmax}_{\{x_i\}_{i=1}^{22}} \sum_{i=1}^{22} c_i x_i,$$

$$\text{subject to} \sum_{i=1}^{22} w_i^+ x_i \le 0 \text{ and } x_i \in \{0,1\}, \quad \text{(Equation 4)}$$

where $\{x_i\}$ are binary indicator variables whether a chromosome is included in the test/hold-out set, w_i^+ and w_i^- are the fractions of disease-associated (w_i^+) and control SNPs (w_i^-) on chromosome I, and weights in the objective function are defined as $c_i = w_i^+ - |w_i^+ - w_i^-|$. This approach selects, for each disease term, a set of chromosomes to hold out that contain about 20% of disease-associates SNPs and that approximately reflects the overall imbalance between disease-associated and control SNPs. Figures S17 and S18 contain performance evaluations on chromosome hold-out sets.

Performance assessment using one SNP per LD block

To assess the effect of SNP correlation on our results, we also performed analyses using only a single representative SNP per LD block (defined by $r^2 \ge 0.5$; see disease-associated variants). The results are shown in Figures S19 and S20.

Comparison with genetic correlation

We retrieved genetic correlation values of disease pairs from the GWAS Atlas.[34] To be able to use these data, we mapped EFO disease terms (used in the NIH-NCBI GWAS Catalog and in our study) to terms used in the GWAS Atlas study. To do so, we extracted synonyms of each EFO term (as listed on EFO ontology) and compared each synonym to the "trait" and "uniqtrait" columns in the GWAS Atlas data. All matches (with one tolerated letter substitution) were used.

In this approach, a single EFO term can map to multiple GWAS Atlas traits and studies. To estimate the genetic correlation between two EFO terms (say, d_i and d_j), we use a weighted combination of genetic correlation values:

$$r_g(d_i, d_j) = \sum_{l,m} W_{lm} r_g\left(s(d_i)_l, s(d_j)_m\right), \quad \text{(Equation 5)}$$

where $r_g(\cdot,\cdot)$ is the genetic correlation of two diseases, $\{s(d_i)\}_{i=1}^r$ and $\{s(d_j)\}_{j=1}^s$ are the GWAS Atlas studies that are mapped to EFO term d_i and d_j, respectively, and w_{lm} is a weight for each combination of the GWAS Atlas studies accounting for the sample sizes of different studies used to estimate genetic correlation values. We choose

$$w_{lm} = \tilde{w}(s(d_i)_l) \cdot \tilde{w}\left(s(d_j)_m\right), \quad \text{(Equation 6)}$$

where

$$\tilde{w}(s(d_i)_l) = size(s(d_i)_l) \Big/ \sum_k \text{size}(s(d_i)_k), \quad \text{(Equation 7)}$$

where "size" denotes the sample size of a study. This scheme puts higher weights on studies with large sample sizes and smaller weights on studies with smaller sample sizes.

Notes about epimap comparison, cluster annotation, and display

Epimap trait-tissue association

To create Table 5, we obtained the latest SNP-centric GWAS enrichments table from the EpiMap Repository at http://compbio.mit.edu/epimap/. We retrieve tissues with adjusted p values for each disease. We map the tissue names used in our study (Standard Roadmap Epigenomes, as labeled by EID) to tissue names used in epimap (biosamples, as labeled by BSS biosample ID) by adapting the scripts from https://github.com/cboix/EPIMAP_ANALYSIS/blob/master/metadata_scripts/get_roadmap_mapping.R. If there are more than one biosample tissues mapped to roadmap tissues, then we report the p value of the tissue with the most significant result.

Disease group names

To name each cluster/group of diseases/EFO terms in Table 6, we choose the EFO term that contains most of the cluster/group members. In Data SD21, we summarize the terms with high term frequency in each cluster, where term frequency is the fraction of the descendant terms present. For example, the EFO term "immune system disease" (EFO: 0000540) has a term frequency of 0.588 in the "immune-1 cluster"; this means that 58.8% of EFO terms in that cluster are descendants of EFO: 0000540. We exclude the terms that are overly broad such as the term "disease" or "experimental factor ontology." For each cluster, we rank the cluster member EFO terms using term frequency and select as the name a meaningful term with the high term frequency. For one cluster where no term had high frequency, we chose the name "heterogeneous."

We also show a diagrams of EFO disease term relationships in each cluster in Figures S10–S126. Occasionally, we include ancestor EFO terms not present in the cluster in a diagram, which are marked by asterisks.

Dimension reduction and coefficient heatmap

UMAP plot: the two-dimensional UMAP plot of the 111 EFO disease terms in Figure 8 is based on disease similarities based on context-weighted score averaging (see disease similarities from context-weighted score averaging). The umap function of the uwotR package was used with the parameters n neighbors = 15, ret model = TRUE, and PCA center = FALSE.

Coefficient heatmap: the heatmap in Figure 9 displays coefficient vectors of models for disease association (see a penalized

logistic regression model for context-weighted score averaging), normalized for each disease. Specifically, for each disease and tissue coefficient x_i,

$$\tilde{x}_i = \begin{cases} (x_i - x_{\min})/x_{95} & x_i \leq x_{95} \\ 1 & x_i > x_{95} \end{cases}, \quad \text{(Equation 8)}$$

where $x_{\min}$ is the minimum coefficient for a disease and x_{95} is the 95% quantile.

Cluster-associated tissues: for each cluster, we show the top five tissues that are most associated with the cluster (Figure 9). To identify these tissues, we conduct a two-sample Wilcoxon test (one-sided) on every tissue, where we compare normalized tissue coefficients for this cluster to the other with the highest coefficients on average. The five tissues with the smallest p value are then selected as the top five tissues.

Tissue-associated clusters: for the heatmap with all tissues in Figure S9, we assigned a cluster to each tissue. For each tissue, we calculated the median (across disease terms of a cluster) of the normalized coefficients for all clusters; the cluster with the highest median was assigned.

Data and code availability

Public data repositories were used as detailed in the materials and methods section, and data underlying the tables and figures are available as supplemental information online. 25-bp-resolution tissue-weighted DHS scores are available for download at https://doi.org/10.7910/DVN/AUAJ7K, and the computer code used to generate the analyses presented is available at https://github.com/kostkalab/nc-gwassnps-score_manuscript.

Supplemental information

Supplemental information can be found online at https://doi.org/10.1016/j.xhgg.2024.100310.

Declaration of interests

The authors declare no competing interests.

Received: October 25, 2023
Accepted: May 16, 2024

Web resources

CADD v.1.3, http://krishna.gs.washington.edu/download/CADD/v1.3/1000G_phase3.tsv.gz

EpiMap, http://compbio.mit.edu/epimap/

DIVAN, https://sites.google.com/site/emorydivan

EigenPC v.1.1, https://xioniti01.u.hpc.mssm.edu/v1.1

EMBL-EBI EFO, https://www.ebi.ac.uk/efo

Fitcons2, http://compgen.cshl.edu/fitCons2/hg19

GenoCanyon, ttp://genocanyon.med.yale.edu/GenoCanyon_Downloads.html

GenoSkyline, http://genocanyon.med.yale.edu/GenoSkylineFiles/GenoSkylinePlus/GenoSkylinePlus_bed.tar.gz

GitHub, https://github.com/cboix/EPIMAP_ANALYSIS/blob/master/metadata_scripts/get_roadmap_mapping.R

GWAVA, ftp://ftp.sanger.ac.uk/pub/resources/software/gwava/v1.0/VEP_plugin/gwava_scores.bed.gz

LINSIGHT, http://compgen.cshl.edu/%7Eyihuang/tracks/LINSIGHT.bw

NCBI gapplus, https://www.ncbi.nlm.nih.gov/projects/gapplus/sgap_plus.htm

DNase Hypersensitivity, https://noble.gs.washington.edu/proj/avocado/data/avocado_full/DNase/

References

1. Buniello, A., MacArthur, J.A.L., Cerezo, M., Harris, L.W., Hayhurst, J., Malangone, C., McMahon, A., Morales, J., Mountjoy, E., Sollis, E., et al. (2019). The NHGRI-EBI GWAS Catalog of published genome-wide association studies, targeted arrays and summary statistics 2019. Nucleic Acids Res. *47*, D1005–D1012. https://doi.org/10.1093/nar/gky1120.
2. An, J.-Y., Lin, K., Zhu, L., Werling, D.M., Dong, S., Brand, H., Wang, H.Z., Zhao, X., Schwartz, G.B., Collins, R.L., et al. (2018). Genome-wide de novo risk score implicates promoter variation in autism spectrum disorder. Science (New York, N.Y.) *362*, eaat6576. https://doi.org/10.1126/science.aat6576.
3. Turro, E., Astle, W.J., Megy, K., Gräf, S., Greene, D., Shamardina, O., Allen, H.L., Sanchis-Juan, A., Frontini, M., Thys, C., et al. (2020). Whole-genome sequencing of patients with rare diseases in a national health system. Nature *583*, 96–102. https://doi.org/10.1038/s41586-020-2434-2.
4. Lindblad-Toh, K., Garber, M., Zuk, O., Lin, M.F., Parker, B.J., Washietl, S., Kheradpour, P., Ernst, J., Jordan, G., Mauceli, E., et al. (2011). A high-resolution map of human evolutionary constraint using 29 mammals. Nature *478*, 476–482. https://doi.org/10.1038/nature10530.
5. Roadmap Epigenomics Consortium, Kundaje, A., Meuleman, W., Ernst, J., Bilenky, M., Yen, A., Heravi-Moussavi, A., Kheradpour, P., Zhang, Z., Wang, J., et al. (2015). Integrative analysis of 111 reference human epigenomes. Nature *518*, 317–330. https://doi.org/10.1038/nature14248.
6. ENCODE Project Consortium, Moore, J.E., Purcaro, M.J., Pratt, H.E., Epstein, C.B., Shoresh, N., Adrian, J., Kawli, T., Davis, C.A., Dobin, A., et al. (2020). Expanded encyclopaedias of DNA elements in the human and mouse genomes. Nature *583*, 699–710. https://doi.org/10.1038/s41586-020-2493-4.
7. Lee, P.H., Lee, C., Li, X., Wee, B., Dwivedi, T., and Daly, M. (2018). Principles and methods of in-silico prioritization of non-coding regulatory variants. Hum. Genet. *137*, 15–30. https://doi.org/10.1007/s00439-017-1861-0.
8. Kircher, M., Witten, D.M., Jain, P., O'Roak, B.J., Cooper, G.M., and Shendure, J. (2014). A general framework for estimating the relative pathogenicity of human genetic variants. Nat. Genet. *46*, 310–315. https://doi.org/10.1038/ng.2892.
9. Ionita-Laza, I., McCallum, K., Xu, B., and Buxbaum, J.D. (2016). A spectral approach integrating functional genomic annotations for coding and noncoding variants. Nat. Genet. *48*, 214–220. https://doi.org/10.1038/ng.3477.
10. Huang, Y.-F., Gulko, B., and Siepel, A. (2017). Fast, scalable prediction of deleterious noncoding variants from functional and population genomic data. Nat. Genet. *49*, 618–624. https://doi.org/10.1038/ng.3810.
11. Lu, Q., Powles, R.L., Wang, Q., He, B.J., and Zhao, H. (2016). Integrative tissue-specific functional annotations in the human genome provide novel insights on many complex traits

and improve signal prioritization in genome wide association studies. PLoS Genet. *12*, e1005947.

12. Gulko, B., and Siepel, A. (2019). An evolutionary framework for measuring epigenomic information and estimating cell-type-specific fitness consequences. Nat. Genet. *51*, 335–342. https://doi.org/10.1038/s41588-018-0300-z.
13. Backenroth, D., He, Z., Kiryluk, K., Boeva, V., Pethukova, L., Khurana, E., Christiano, A., Buxbaum, J.D., and Ionita-Laza, I. (2018). FUN-LDA: A Latent Dirichlet Allocation Model for Predicting Tissue-Specific Functional Effects of Noncoding Variation: Methods and Applications. Am. J. Hum. Genet. *102*, 920–942. https://doi.org/10.1016/j.ajhg.2018.03.026.
14. He, Z., Liu, L., Wang, K., and Ionita-Laza, I. (2018). A semi-supervised approach for predicting cell-type specific functional consequences of non-coding variation using MPRAs. Nat. Commun. *9*, 5199. https://doi.org/10.1038/s41467-018-07349-w.
15. Zhang, S., He, Y., Liu, H., Zhai, H., Huang, D., Yi, X., Dong, X., Wang, Z., Zhao, K., Zhou, Y., et al. (2019). regBase: whole genome base-wise aggregation and functional prediction for human non-coding regulatory variants. Nucleic Acids Res. *47*, e134. https://doi.org/10.1093/nar/gkz774.
16. Vervier, K., and Michaelson, J.J. (2018). TiSAn: estimating tissue-specific effects of coding and non-coding variants. Bioinformatics *34*, 3061–3068. https://doi.org/10.1093/bioinformatics/bty301.
17. Jia, P., Dai, Y., Hu, R., Pei, G., Manuel, A.M., and Zhao, Z. (2020). TSEA-DB: a trait–tissue association map for human complex traits and diseases. Nucleic Acids Res. *48*, D1022–D1030. https://doi.org/10.1093/nar/gkz957.
18. Boix, C.A., James, B.T., Park, Y.P., Meuleman, W., and Kellis, M. (2021). Regulatory genomic circuitry of human disease loci by integrative epigenomics. Nature *590*, 300–307. https://doi.org/10.1038/s41586-020-03145-z.
19. Richter, F., Morton, S.U., Kim, S.W., Kitaygorodsky, A., Wasson, L.K., Chen, K.M., Zhou, J., Qi, H., Patel, N., DePalma, S.R., et al. (2020). Genomic analyses implicate noncoding de novo variants in congenital heart disease. Nat. Genet. *52*, 769–777. https://doi.org/10.1038/s41588-020-0652-z.
20. Yousefian-Jazi, A., Sung, M.K., Lee, T., Hong, Y.-H., Choi, J.K., and Choi, J. (2020). Functional fine-mapping of noncoding risk variants in amyotrophic lateral sclerosis utilizing convolutional neural network. Sci. Rep. *10*, 12872. https://doi.org/10.1038/s41598-020-69790-6.
21. Yousefian-Jazi, A., Jung, J., Choi, J.K., and Choi, J. (2020). Functional annotation of noncoding causal variants in autoimmune diseases. Genomics *112*, 1208–1213. https://doi.org/10.1016/j.ygeno.2019.07.006.
22. Chen, L., Jin, P., and Qin, Z.S. (2016). DIVAN: accurate identification of non-coding disease-specific risk variants using multi-omics profiles. Genome Biol. *17*, 252. https://doi.org/10.1186/s13059-016-1112-z.
23. Bodea, C.A., Mitchell, A.A., Bloemendal, A., Day-Williams, A.G., Runz, H., and Sunyaev, S.R. (2018). PINES: phenotype-informed tissue weighting improves prediction of pathogenic noncoding variants. Genome Biol. *19*, 173. https://doi.org/10.1186/s13059-018-1546-6.
24. Gao, L., Uzun, Y., Gao, P., He, B., Ma, X., Wang, J., Han, S., and Tan, K. (2018). Identifying noncoding risk variants using disease-relevant gene regulatory networks. Nat. Commun. *9*, 702. https://doi.org/10.1038/s41467-018-03133-y.
25. Pers, T.H., Timshel, P., and Hirschhorn, J.N. (2015). SNPsnap: a Web-based tool for identification and annotation of matched SNPs. Bioinformatics *31*, 418–420. https://doi.org/10.1093/bioinformatics/btu655.
26. Lu, Q., Hu, Y., Sun, J., Cheng, Y., Cheung, K.-H., and Zhao, H. (2015). A Statistical Framework to Predict Functional Non-Coding Regions in the Human Genome Through Integrated Analysis of Annotation Data. Sci. Rep. *5*, 10576. https://doi.org/10.1038/srep10576.
27. Ritchie, G.R.S., Dunham, I., Zeggini, E., and Flicek, P. (2014). Functional annotation of noncoding sequence variants. Nat. Methods *11*, 294–296. https://doi.org/10.1038/nmeth.2832.
28. Kraaij, M.D., and van Laar, J.M. (2008). The role of B cells in systemic sclerosis. Biologics. *2*, 389–395.
29. Thoreau, B., Chaigne, B., and Mouthon, L. (2022). Role of B-cell in the pathogenesis of systemic sclerosis. Front. Immunol. *13*, 933468.
30. (US), M.I.B.M.N.L.o.M. (2023). Primary Sclerosing Cholangitis.
31. Kunzmann, L.K., Schoknecht, T., Poch, T., Henze, L., Stein, S., Kriz, M., Grewe, I., Preti, M., Hartl, J., Pannicke, N., et al. (2020). Monocytes as potential mediators of pathogen-induced T-helper 17 differentiation in patients with primary sclerosing cholangitis (PSC). Hepatology *72*, 1310–1326.
32. Keku, T.O., Galanko, J.A., Murray, S.C., Woosley, J.T., and Sandler, R.S. (1998). Rectal mucosal proliferation, dietary factors, and the risk of colorectal adenomas. Cancer Epidemiol. Biomarkers Prev. *7*, 993–999.
33. Dulal, S., and Keku, T.O. (2014). Gut microbiome and colorectal adenomas. Cancer J. *20*, 225–231.
34. Watanabe, K., Stringer, S., Frei, O., Umićević Mirkov, M., de Leeuw, C., Polderman, T.J.C., van der Sluis, S., Andreassen, O.A., Neale, B.M., and Posthuma, D. (2019). A global overview of pleiotropy and genetic architecture in complex traits. Nat. Genet. *51*, 1339–1348. https://doi.org/10.1038/s41588-019-0481-0.
35. Yang, Y., Musco, H., Simpson-Yap, S., Zhu, Z., Wang, Y., Lin, X., Zhang, J., Taylor, B., Gratten, J., and Zhou, Y. (2021). Investigating the shared genetic architecture between multiple sclerosis and inflammatory bowel diseases. Nat. Commun. *12*, 5641. https://doi.org/10.1038/s41467-021-25768-0.
36. Farh, K.K.H., Marson, A., Zhu, J., Kleinewietfeld, M., Housley, W.J., Beik, S., Shoresh, N., Whitton, H., Ryan, R.J.H., Shishkin, A.A., et al. (2015). Genetic and epigenetic fine mapping of causal autoimmune disease variants. Nature *518*, 337–343. https://doi.org/10.1038/nature13835.
37. McDowell, C., Farooq, U., and Haseeb, M. (2022). Inflammatory Bowel Disease. In StatPearls (StatPearls Publishing LLC).
38. Lord, C., Elsabbagh, M., Baird, G., and Veenstra-Vanderweele, J. (2018). Autism spectrum disorder. Lancet *392*, 508–520. https://doi.org/10.1016/s0140-6736(18)31129-2.
39. Olivo, G., Gaudio, S., and Schiöth, H.B. (2019). Brain and Cognitive Development in Adolescents with Anorexia Nervosa: A Systematic Review of fMRI Studies. Nutrients *11*, 1907. https://doi.org/10.3390/nu11081907.
40. Sigmon, E.R., Kelleman, M., Susi, A., Nylund, C.M., and Oster, M.E. (2019). Congenital Heart Disease and Autism: A Case-Control Study. Pediatrics *144*, e20184114. https://doi.org/10.1542/peds.2018-4114.
41. Boltri, M., and Sapuppo, W. (2021). Anorexia Nervosa and Autism Spectrum Disorder: A Systematic Review. Psychiatr. Res. *306*, 114271. https://doi.org/10.1016/j.psychres.2021.114271.

42. Zhou, Z.C., McAdam, D.B., and Donnelly, D.R. (2018). Endophenotypes: A conceptual link between anorexia nervosa and autism spectrum disorder. Res. Dev. Disabil. *82*, 153–165. https://doi.org/10.1016/j.ridd.2017.11.008.
43. Tylee, D.S., Sun, J., Hess, J.L., Tahir, M.A., Sharma, E., Malik, R., Worrall, B.B., Levine, A.J., Martinson, J.J., Nejentsev, S., et al. (2018). Genetic correlations among psychiatric and immune-related phenotypes based on genome-wide association data. Am. J. Med. Genet. B Neuropsychiatr. Genet. *177*, 641–657. https://doi.org/10.1002/ajmg.b.32652.
44. Li, C.Y., Yang, T.M., Ou, R.W., Wei, Q.Q., and Shang, H.F. (2021). Genome-wide genetic links between amyotrophic lateral sclerosis and autoimmune diseases. BMC Med. *19*, 27. https://doi.org/10.1186/s12916-021-01903-y.
45. Yu, X., Vargas, J., Green, P.H.R., and Bhagat, G. (2021). Innate Lymphoid Cells and Celiac Disease: Current Perspective. Cell. Mol. Gastroenterol. Hepatol. *11*, 803–814. https://doi.org/10.1016/j.jcmgh.2020.12.002.
46. Jabri, B., and Sollid, L.M. (2017). T Cells in Celiac Disease. J. Immunol. *198*, 3005–3014. https://doi.org/10.4049/jimmunol.1601693.
47. Ming, J., Dai, M., Cai, M., Wan, X., Liu, J., and Yang, C. (2018). LSMM: a statistical approach to integrating functional annotations with genome-wide association studies. Bioinformatics *34*, 2788–2796. https://doi.org/10.1093/bioinformatics/bty187.
48. Julià, A., López-Longo, F.J., Pérez Venegas, J.J., Bonàs-Guarch, S., Olivé, À., Andreu, J.L., Aguirre-Zamorano, M.Á., Vela, P., Nolla, J.M., de la Fuente, J.L.M., et al. (2018). Genome-wide association study meta-analysis identifies five new loci for systemic lupus erythematosus. Arthritis Res. Ther. *20*, 100. https://doi.org/10.1186/s13075-018-1604-1.
49. Lu, H., Zhang, J., Jiang, Z., Zhang, M., Wang, T., Zhao, H., and Zeng, P. (2021). Detection of Genetic Overlap Between Rheumatoid Arthritis and Systemic Lupus Erythematosus Using GWAS Summary Statistics. Front. Genet. *12*, 656545. https://doi.org/10.3389/fgene.2021.656545.
50. Bentham, J., Morris, D.L., Graham, D.S.C., Pinder, C.L., Tombleson, P., Behrens, T.W., Martín, J., Fairfax, B.P., Knight, J.C., Chen, L., et al. (2015). Genetic association analyses implicate aberrant regulation of innate and adaptive immunity genes in the pathogenesis of systemic lupus erythematosus. Nat. Genet. *47*, 1457–1464. https://doi.org/10.1038/ng.3434.
51. Li, X., Li, Z., Zhou, H., Gaynor, S.M., Liu, Y., Chen, H., Sun, R., Dey, R., Arnett, D.K., Aslibekyan, S., et al. (2020). Dynamic incorporation of multiple in silico functional annotations empowers rare variant association analysis of large whole-genome sequencing studies at scale. Nat. Genet. *52*, 969–983. https://doi.org/10.1038/s41588-020-0676-4.
52. Li, Z., Li, X., Zhou, H., Gaynor, S.M., Selvaraj, M.S., Arapoglou, T., Quick, C., Liu, Y., Chen, H., Sun, R., et al. (2022). A framework for detecting noncoding rare-variant associations of large-scale whole-genome sequencing studies. Nat. Methods *19*, 1599–1611. https://doi.org/10.1038/s41592-022-01640-x.
53. Malone, J., Holloway, E., Adamusiak, T., Kapushesky, M., Zheng, J., Kolesnikov, N., Zhukova, A., Brazma, A., and Parkinson, H. (2010). Modeling sample variables with an Experimental Factor Ontology. Bioinformatics *26*, 1112–1118. https://doi.org/10.1093/bioinformatics/btq099.
54. Schreiber, J., Bilmes, J., and Noble, W.S. (2020). Completing the ENCODE3 compendium yields accurate imputations across a variety of assays and human biosamples. Genome Biol. *21*, 82. https://doi.org/10.1186/s13059-020-01978-5.
55. Friedman, J., Hastie, T., and Tibshirani, R. (2010). Regularization Paths for Generalized Linear Models via Coordinate Descent. J. Stat. Software *33*, 1–22.
56. Vanwinckelen, G., and Blockeel, H. (2012). On estimating model accuracy with repeated cross-validation. In BeneLearn 2012: Proceedings of the 21st Belgian-Dutch Conference on Machine Learning, pp. 39–44.
57. Demšar, J. (2006). Statistical comparisons of classifiers over multiple data sets. J. Mach. Learn. Res. *7*, 1–30.
58. Ramos, E.M., Hoffman, D., Junkins, H.A., Maglott, D., Phan, L., Sherry, S.T., Feolo, M., and Hindorff, L.A. (2014). Phenotype-Genotype Integrator (PheGenI): synthesizing genome-wide association study (GWAS) data with existing genomic resources. Eur. J. Hum. Genet. *22*, 144–147. https://doi.org/10.1038/ejhg.2013.96.

HGGA, Volume *5*

Supplemental information

Disease-specific prioritization of non-coding GWAS variants based on chromatin accessibility

Qianqian Liang, Abin Abraham, John A. Capra, and Dennis Kostka

Contents

1 Supplemental Methods 2

2 Supplemental Tables 3

3 Supplemental Data Legends 6

SD1 Phenotypes used in this study 6
SD2 Disease-associated SNVs used in this study 6
SD3 Control SNVs used in this study 6
SD4 Pairwise comparisons of organism-level scores for each disease term 6
SD5 Pairwise comparisons of organism-level scores, aggregated across diseases 6
SD6 Pairwise comparison of Tissue-weighted scores vs. Tissue-mean scores for each disease term 7
SD7 Pairwise comparison of Tissue-weighted scores vs. Tissue-mean scores, aggregated across diseases 7
SD8 Pairwise comparison of three Tissue-weighted scores for each disease term 7
SD9 Pairwise comparison of three Tissue-weighted scores, aggregated across diseases 7
SD10 Pairwise comparison of Tissue-weighted-DHS vs five organism-level scores for each disease term 7
SD11 Pairwise comparison of tissue-weighted-DHS and five organism-level scores aggregated . 8
SD12 Mapping of mesh terms to EFO terms 8
SD13 Training and test SNVs used to compare Tissue-weighted with DIVAN (including disease and matched control SNVs) 8
SD14 Pairwise comparison of DIVAN vs. GenoCanyon vs Tissue-weighted-DHS for each disease term 8
SD15 GenoCanyon vs DIVAN in our study and in Chen study (the DIVAN study) 8
SD16 Tissue-weighted prediction scores for SNVs across 111 diseases 9
SD17 Beta coefficients of the logistic regression models in 111 diseases (using DHS score) . . . 9
SD18 Standard deviation of the beta coefficients in SD1 9
SD19 Disease-disease similarities derived from the logistic regression model (DHS) 9
SD20 Clusters assigned to 111 diseases 9
SD21 Term frequency in 7 disease clusters 9
SD22 Top five tissues in 7 disease clusters 9
SD23 ID, name and group of standard epigenomes 9
SD24 Genetic correlation of the disease pairs 10
SD25 Performance of Tissue-weighted (DHS) in different held-out strategies 10

4 Supplemental Figures 10

1 Supplemental Methods

Four different matching strategies for control SNVs

For each disease-associated SNV, we have matched control SNVs from four different matching strategies: 1) random; 2) SNPsnap_TSS; 3) SNPsnap; and 4) TSS (see **Methods**). We measured the performance of five organism level scores on four different control sets in 111 diseases. Among them, random matching is considered as the least stringent way as we don't have any constraint on it. Therefore, we choose the random matching as the baseline, and we normalize the performance of the other three control sets on random matching for each disease. We plot the normalized performance in **Suppl. Fig.** S1 and S2, using each disease as a panel.

From here, we observe that three normalized performances in CADD are all distributed around 1. This indicates that the CADD is robust in different matching strategies. The normalized performances of Eigen, GenoCanyon, GWAVA and LINSIGHT are all less than 1. This indicates that those three matched control sets are more stringent than randomly selecting control variants. Among those three control sets, TSS is the most stringent, followed by SNPsnap and SNPsnap_TSS.

It is important to note that TSS and SNPsnap TSS are both matched using the distance to the nearest TSS; however, TSS uses the distance to the nearest protein-coding gene while SNPsnap_TSS uses the distance to the nearest gene. TSS matched SNVs have similar distribution to disease SNVs in both all genes and protein-coding genes; in contrast, SNPsnap_TSS SNVs have similar distribution to disease SNVs in all genes but not in protein-coding genes (**Suppl. Fig.** S3 and S4). Therefore, TSS is more stringent than SNPsnap_TSS and also more stringent than SNPsnap even though SNPsnap has matched with additional three criteria.

Here, SNPsnap matching strategy is neither too stringent nor too loose and it matches with four criteria (see **Methods**). Thus, we choose the control set matched by SNPsnap in our study.

DHS-weighted performance using two additional strategies to prevent overfitting

To prevent overfitting, we also deployed two additional strategies to test the performance of tissue-weighted DHS. In the first one, we used the 'representative SNVs' so that any two disease-associated SNVs are not in the same LD block. In the second one, we deployed a chromosome held-out strategy so that the SNVs in the test and train set are on different chromosomes (see **Methods**). These two strategies ensure that the SNVs in the test and train set are seperated or in different chromosome to reduce overfitting. We observe that in any of these two settings, we can still observe a significant increase with the tissue-weighted model, which is consistent with our previous finding, even though the amount of the improvement is in a lesser degree in some diseases (See See **Suppl. Fig.** S17-S20).

2 Supplemental Tables

Score	Wins	Losses	Ties	Wins (agg)	Losses (agg)	Ties (agg)
GenoCanyon	335	87	22	4	0	0
LINSIGHT	303	124	17	3	1	0
eigen	247	177	20	2	2	0
GWAVA	168	256	20	1	3	0
CADD	15	424	5	0	4	0

Supplemental Table ST1: ***Relative performance of organism-level variant scores, measured by AUROC***. Wins, Losses, Ties refers to significantly better (or worse, or tied) performance across all possible pairings (see **Methods**). The first three columns summarize separate comparisons for each disease term (for each row there are four other methods and 111 terms), while the last three columns represent results of aggregate comparisons across terms. Average precision was used as the performance metric, and the Wilcoxon singed-ranks test to determine wins and losses (p-values less than 0.05 were ties).

Score	Wins	Losses	Ties	Wins (agg)	Losses (agg)	Ties (agg)
DHS	138	54	30	2	10	0
Fitcons2	80	111	31	0	1	1
Genoskyline	69	122	31	0	1	1

Supplemental Table ST2: ***Relative performance of disease-specific (tissue-weighted) variant scores, measured by AUROC***. Wins, Losses, Ties refers to significantly better (or worse, or tied) performance across all possible pairings (see **Methods**). The first three columns summarize separate comparisons for each disease term (for each row there are two other methods and 111 terms), while the last three columns represent results of aggregate comparisons across terms. Average precision was used as the performance metric, and the Wilcoxon singed-ranks test to determine wins and losses (p-values less than 0.05 were ties).

Score/Method	By disease term Wins	Losses	Ties	Aggregated Wins	Losses	Ties
DHS	375	127	53	4	0	1
GenoCanyon	375	144	36	4	0	1
LINSIGHT	342	184	29	3	2	0
eigen	273	250	32	2	3	0
GWAVA	186	338	31	1	4	0
CADD	19	527	9	0	5	0

Supplemental Table ST3: ***DHS outperforms organism-level variant scores, measured by AUROC***. Wins, Losses, Ties refer to significantly better (or worse, or tied) performance across all possible score pairings (see **Methods**). The first three columns summarize separate comparisons for each disease term (for each row there are two other methods and 111 terms, i.e., 555 comparisons), while the last three columns represent results of comparisons aggregated over terms. Average precision was used as the performance metric, and the Wilcoxon singed-ranks test to determine wins and losses (p-values less than 0.05 were reported as ties).

Score/Method	Performance vs. DHS Wins	Losses	Ties	Winning percent
LINSIGHT	17	84	10	20
GenoCanyon	7	92	12	12
GWAVA	12	93	6	14
eigen	4	98	8	8
CADD	4	107	2	4

Supplemental Table ST4: ***Disease-specific variant prioritization outperforms organism-level approaches, measured by AUPR***. Wins losses and ties of organism-level scores against tissue-weighted DHS scores (performance measured by average precision, Wilcoxon signed-ranks test for determining significance). Winning percent was calculated as number of wins plus half the number of ties, divided by the number of comparisons, and rounded to the nearest integer. Rows have been ordered by winning percent.

Score/Method	Performance vs. DHS Wins	Losses	Ties	Winning percent
GenoCanyon	40	57	14	36
LINSIGHT	39	60	12	35
eigen	26	73	12	23
GWAVA	18	82	11	16
CADD	4	103	4	4

Supplemental Table ST5: ***Disease-specific variant prioritization outperforms organism-level approaches, measured by AUROC***. Wins losses and ties of organism-level scores against tissue-weighted DHS scores (performance measured by average precision, Wilcoxon signed-ranks test for determining significance). Winning percent was calculated as number of wins plus half the number of ties, divided by the number of comparisons, and rounded to the nearest integer. Rows have been ordered by winning percent.

Score	Wins	Losses	Ties	Winning percent
GenoCanyon	31	25	2	55
DHS	27	26	5	51
DIVAN	24	31	3	44

Supplemental Table ST6: ***DHS tissue-weighted disease-specific scoring outperforms DIVAN***. Across 30 disease terms, this table summarizes all pairwise comparison for DHS tissue-weighted, GenoCanyon and DIVAN using a specifically created test dataset. Wins, losses, ties refer to significantly better (or worse, or tied) performance. Average precision was used as the performance metric, and the Wilcoxon singed-ranks test to determine wins and losses (p-values less than 0.05 were ties). Winning percent = #Wins/(#Wins+#Losses)

Rank	ID	Tissue name	Group	epimap FDR
Systemic scleroderma				
1	E116	GM12878 Lymphoblastoid Cells	blood	0.04
2	E032	Primary B cells from peripheral blood	blood	0.16
3	E041	Primary T helper cells PMA-I stimulated	blood	0.08
4	E123	K562 Leukemia Cells	blood	1.00
5	E030	Primary neutrophils from peripheral blood	blood	0.86
Sclerosing cholangitis				
1	E116	GM12878 Lymphoblastoid Cells	blood	$<$0.001
2	E061	Foreskin Melanocyte Primary Cells skin03	skin	0.18
3	E102	Rectal Mucosa Donor 31	gi_rectum	$<$0.001
4	E041	Primary T helper cells PMA-I stimulated	blood	$<$0.001
5	E029	Primary monocytes from peripheral blood	blood	$<$0.001
Colorectal adenoma				
1	E102	Rectal Mucosa Donor 31	gi_rectum	0.05
2	E110	Stomach Mucosa	gi_stomach	0.008
3	E057	Foreskin Keratinocyte Primary Cells skin02	skin	0.12
4	E101	Rectal Mucosa Donor 29	gi_rectum	0.004
5	E028	Breast variant Human Mammary Epithelial Cells (vHMEC)	breast	0.20
Atrial fibrillation				
1	E083	Fetal Heart	heart	$<$0.001
2	E108	Skeletal Muscle Female	muscle	0.01
3	E107	Skeletal Muscle Male	muscle	0.009
4	E088	Fetal Lung	lung	0.002
5	E120	HSMM Skeletal Muscle Myoblasts Cells	muscle	0.18
Cutaneous melanoma				
1	E061	Foreskin Melanocyte Primary Cells skin03	skin	0.08
2	E059	Foreskin Melanocyte Primary Cells skin01	skin	0.08
3	E117	HeLa-S3 Cervical Carcinoma Cell Line	cervix	0.40
4	E041	Primary T helper cells PMA-I stimulated	blood	0.50
5	E122	HUVEC Umbilical Vein Endothelial Primary Cells	vascular	0.87

Supplemental Table ST7: ***Top-ranked tissues for five diseases.*** For five diseases when show the top-five tissues with the largest tissue weights in the corresponding model we derive. The first column is the tissue rank, the second the tissue's roadmap ID, the third the tissue name, the fourth the tissue group, and the fifth listst the adjusted p-value in an enrichment analysis performed by CA Boix et al. Nature, 590, pages 300–307 (2021).

3 Supplemental Data Legends

SD1 Phenotypes used in this study

Filename: `sup_data_disease-terms.csv.gz`

The first column denotes the EFO name of disease phenotypes used. Column #2 is the EFO ID. Column #3 shows the number of SNVs associated with the term (coding and non-coding). Columns #4 shows the number of non-coding SNVS used in the study before aggregation and #5 shows the number of non-coding SNVs used after aggregation. Non-EUR 1KG SNVs and SNVs in the HLA region have been removed in column #4 and #5.

SD2 Disease-associated SNVs used in this study

Filename: `sup_data_disease-snvs.csv.gz`

The first column denotes the SNV ID. Column #2 is the rsID. Column #3 is the phenotype. Columns #4 and 5 are the chromosome and the specific location (hg19 coordinates). Column #6 is the LD block cluster id where this SNV resides(SNVs in the same LD block will have the same cluster id), and column #7 indicates whether this SNV is selected as the representative SNV for the block (1 as selected, 0 as not selected). SNVs associated with multiple diseases appear in more than one row.

SD3 Control SNVs used in this study

Filename: `sup_data_control-snvs.csv.gz`

For each disease-associated SNV, this table lists $\sim$10 randomly-selected control SNVs by four different methods (see **Methods**). The first column denotes the SNV ID. Column #2 is the rs ID. Column #3 is the phenotype. Column #4 and 5 are the chromosome and the specific location (hg19) of that SNV. Column #6 is the matching strategy (i.e. snpsnap, snpsnap_tss, tss, random) and column #7 is the SNV ID of the corresponding disease-associated SNV.

SD4 Pairwise comparisons of organism-level scores for each disease term

Filename: `sup_data_pairwise-org-individual.csv.gz`

For each combination of organism-level scores we report p-values for a Wilcoxon signed-ranks test for each individual disease (see **Methods**). Column #1 is the score name. Column #2 is the median performance across bootstrap runs for that score. Column #3 is the second score name. Column #4 is the median performance for the second score. Column #5 is the disease term for which the comparison was performed. Column #6 is the curve type we used for the area under the curve performance metric (ROC or PR). Column #7 is the p-value of the test. Column #8 is the score with the higher median.

SD5 Pairwise comparisons of organism-level scores, aggregated across diseases

Filename: `sup_data_pairwise-org-aggregated.csv.gz`

For each combination of organism-level scores we report p-values for a Wilcoxon signed-ranks test, aggregated across 111 diseases (see **Methods**). Column #1 is the score name. Column #2 is the median performance across 111 diseases. Column #3 is the second score name. Column #4 is the median performance for the second score across 111 diseases. Column #5 is the curve type for the area under the curve performance metric (ROC or PR). Column #6 is the p-value of the test. Column #7 is the score with the higher median.

SD6 Pairwise comparison of Tissue-weighted scores vs. Tissue-mean scores for each disease term

Filename: sup_data_pairwise-tis-individual.csv.gz

For each score we report p-values for a Wilcoxon signed-ranks test between the tissue-mean and tissue-weighted version for each individual disease. Column #1 is the score name. Column #2 is the median performance across bootstrap runs for that score. Column #3 is the second score name. Column #4 is the median performance for the second score. Column #5 is the disease term for which the comparison was performed. Column #6 is the curve type we used for the area under the curve performance metric (ROC or average precision). Column #7 is the p-value of the test. Column #8 is the score with the higher median.

SD7 Pairwise comparison of Tissue-weighted scores vs. Tissue-mean scores, aggregated across diseases

Filename: sup_data_pairwise-tis-aggregated.csv.gz

For each score we report p-values for a Wilcoxon signed-ranks test between the tissue-mean and tissue-weighted version, aggregated across all diseases. Column #1 is the score name. Column #2 is the median performance across all diseases for that score. Column #3 is the second score name. Column #4 is the median performance for the second score. Column #5 is the curve type for the area under the curve performance metric (ROC or PR). Column #6 the p-value of the test. Column #7 is the score with the higher median.

SD8 Pairwise comparison of three Tissue-weighted scores for each disease term

Filename: sup_data_pairwise-tis-weighted-individual.csv.gz

For each combination of Tissue-weighted scores we report p-values for a Wilcoxon signed-ranks test for each individual disease (see **Methods**). Column #1 is the score name. Column #2 is the median performance across bootstrap runs for that score. Column #3 is the second score name. Column #4 is the median performance for the second score. Column #5 is the disease term for which the comparison was performed. Column #6 is the curve type we used for the area under the curve performance metric (ROC or PR). Column #7 is the p-value of the test. Column #8 is the score with the higher median.

SD9 Pairwise comparison of three Tissue-weighted scores, aggregated across diseases

Filename: sup_data_pairwise-tis-weighted-aggregated.csv.gz

For each combination of Tissue-weighted scores we report p-values for a Wilcoxon signed-ranks test, aggregated across all diseases (see **Methods**). Column #1 is the score name. Column #2 is the median performance across all diseases for that score. Column #3 is the second score name. Column #4 is the median performance for the second score. Column #5 is the curve type for the area under the curve performance metric (ROC or PR). Column #6 is the p-value of the test. Column #7 is the score with the higher median.

SD10 Pairwise comparison of Tissue-weighted-DHS vs five organism-level scores for each disease term

Filename: sup_data_pairwise-tis-vs-org-individual.csv.gz

We report p-values for a Wilcoxon signed-ranks test between the Tissue-weighted-DHS and five organism-level scores for each individual disease. Column #1 is the score name. Column #2 is the median performance across bootstrap runs for that score. Column #3 is the second score name. Column #4 is the median performance for the second score. Column #5 is the disease term for which the comparison was performed.

Column #6 is the curve type we used for the area under the curve performance metric (ROC or PR). Column #7 is the p-value of the test. Column #8 is the score with the higher median.

SD11 Pairwise comparison of tissue-weighted-DHS and five organism-level scores aggregated

Filename: sup_data_pairwise-tis-org-aggregated.csv.gz

We report p-values for a Wilcoxon signed-ranks test between the Tissue-weighted-DHS and five organism-level scores, aggregated across all diseases. Column #1 is the score name. Column #2 is the median performance across bootstrap runs for that score. Column #3 is the second score name. Column #4 is the median performance for the second score. Column #5 is the curve type we used for the area under the curve performance metric (ROC or PR). Column #6 is the p-value of the test. Column #7 is the score with the higher median.

SD12 Mapping of mesh terms to EFO terms

Filename: sup_data_mapping-efo-mesh.csv.gz

The first and second columns are the mesh term id and mesh term label used by DIVAN. The third and fourth columns are the EFO ID and EFO label that is mapped to the mesh terms. (Note: there are two MeSH terms that are matched to more than 1 EFO term.)

SD13 Training and test SNVs used to compare Tissue-weighted with DIVAN (including disease and matched control SNVs)

Filename: sup_data_divan-snvs.csv.gz

Column #1 denotes the SNV ID. Column #2 is the rs ID of the SNV. Column #3 is the phenotype. Column #4 and #5 are the chromosome and location of the SNV. Column #6 indicates whether the variant is a disease-associated or a control variant. Column #7 is the SNV ID of the corresponding disease-associated SNV. Column #8 indicates whether the variant is in training or test set.

SD14 Pairwise comparison of DIVAN vs. GenoCanyon vs Tissue-weighted-DHS for each disease term

Filename: sup_data_pairwise-divan-individual.csv.gz

For each combination of DIVAN vs. GenoCanyon vs. Tissue-weighted-DHS we report p-values for a Wilcoxon signed-ranks test for each individual disease (see **Methods**). Column #1 is the score name. Column #2 is the median performance across bootstrap runs for that score. Column #3 is the second score name. Column #4 is the median performance for the second score. Column #5 is the disease term for which the comparison was performed. Column #6 is the curve type we used for the area under the curve performance metric (ROC or PR). Column #7 is the p-value of the test. Column #8 is the score with the higher median.

SD15 GenoCanyon vs DIVAN in our study and in Chen study (the DIVAN study)

Filename: sup_data_perf-divan-our-vs-chen.csv.gz

Column #1 is the disease names of 27 overlapping diseases. Column #2 indicates whether GenoCanyon is better than DIVAN in our study. Column #3 indicates whether GenoCanyon is better than DIVAN as published by DIVAN.

SD16 Tissue-weighted prediction scores for SNVs across 111 diseases

Filename: `sup_data_prediction-scores-dhs-weighted.csv.gz`

Column #1 is the the SNV_ID (chr:position). (If a SNV is annotated to multiple phenotypes, there will be multiple entries.) Column #2 is the phenotype that is annotated to the SNVs. Column #3 indicates whether this SNV is a disease-associated variant or a control variant. Column #4-6 are Tissue-weighted prediction scores in Genoskyline, DHS and Fitcons2

SD17 Beta coefficients of the logistic regression models in 111 diseases (using DHS score)

Filename: `sup_data_beta-coefficients-mean-dhs.csv.gz`

Column #1 is the phenotypes. Column #2-128 are the mean of the coefficients of 127 tissues.

SD18 Standard deviation of the beta coefficients in SD1

Filename: `sup_data_beta-coefficients-sd-dhs.csv.gz`

Column #1 is the phenotypes. Column #2-128 are the standard deviation of the coefficients in 127 tissues.

SD19 Disease-disease similarities derived from the logistic regression model (DHS)

Filename: `sup_data_beta-model-similarity-dhs.csv.gz`

column #1 and column #2 are the names of the disease pairs. Column #3 is the weighted disease-disease similarity derived from the model.

SD20 Clusters assigned to 111 diseases

Filename: `sup_data_cluster-id-name.csv.gz`

Column #1 is the disease name. Column #2 is the cluster id. Column #3 is the cluster name.

SD21 Term frequency in 7 disease clusters

Filename: `sup_data_cluster_term_frequency.csv.gz`

Column #1 is the term name. Column #2 is the term id. Column #3 is the term frequency of a term in the cluster. Column #4 is cluster id. Term frequency means the fraction of diseases in this cluster that is a descendant of this term. For example, immune system disease with a term frequency 0.588 in cluster immune-1 means that 58.8% of diseases in immune-1 cluster is a immune system disease.

SD22 Top five tissues in 7 disease clusters

Filename: `sup_data_top-five-tissues.csv.gz`

Column #1 and #2 are the cluster id and name. Column #3-5 are the tissue id, tissue name and tissue anatomy.

SD23 ID, name and group of standard epigenomes

Filename: `sup_data_standard-epigenomes`

Column #1 is the ID of the standard epigenomes (e.g. E043). Column #2 is the group name (e.g. Blood & T-cell). Column #3 is the standardized epigenome name. Column #4 is the anatomy(e.g. blood). Column #5 is the type (e.g. PrimaryCell).

SD24 Genetic correlation of the disease pairs

`Filename: sup_data_genetic-correlation.csv.gz`

column #1 and column #2 are the name of the disease pairs. Column #3 is the genetic correlation derived from the GWAS ATLAS

SD25 Performance of Tissue-weighted (DHS) in different held-out strategies

`Filename: sup_data_perf-chrom-heldout.csv.gz`

Column #1 is the disease name. Column #2-#10 are the performance of Tissue-weighted (DHS) and Tissue-mean (DHS) measured in different held-out strategies. CV-B: cross-validation, baseline; CV-LR: cross-validation logistic regression; CV-LR (SD): standard deviation of CV-LR; random-B: randomly sampled test set, baseline; random-B (SD): random-B standard deviation; random-LR: randomly sample test set, logistic regression; random-LR (SD): random-LR standard deviation; chr-B: test set held out by chromosome, baseline; chr-B (SD): chr-B standard deviation.

4 Supplemental Figures

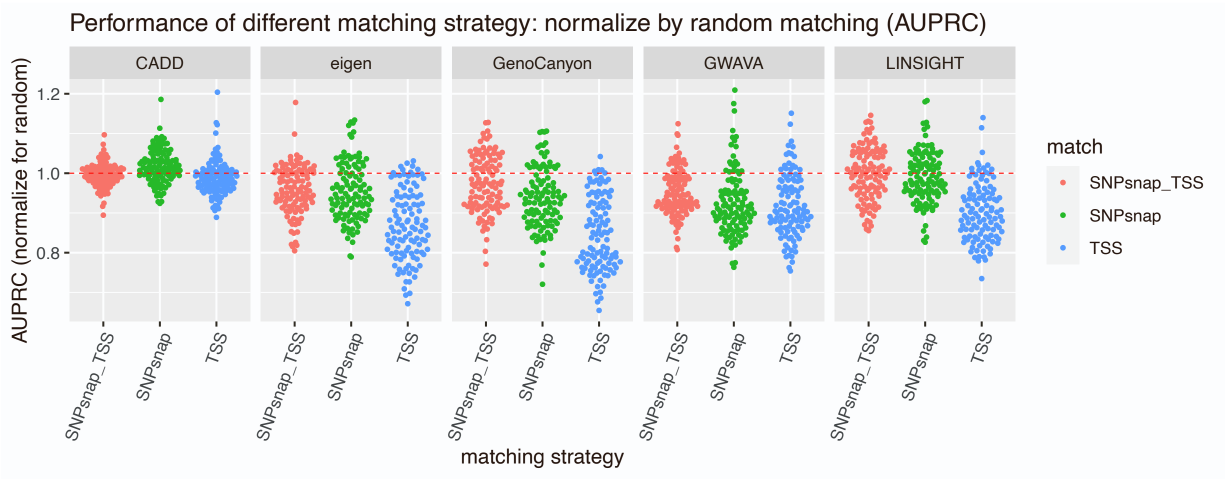

Supplemental Figure S1: ***Performance of different matching strategy, measured by area under the PR curve***. X-axis delineates three different matching strategies (i.e. snpsnap-tss, snpsnap, tss). Y axis shows the performance in terms of area under precision recall curve, normalized by random matching. Each point represents a specific disease term. Horizontal lines spanning the dataset denotes the scenario that the normalized performance equals to 1.

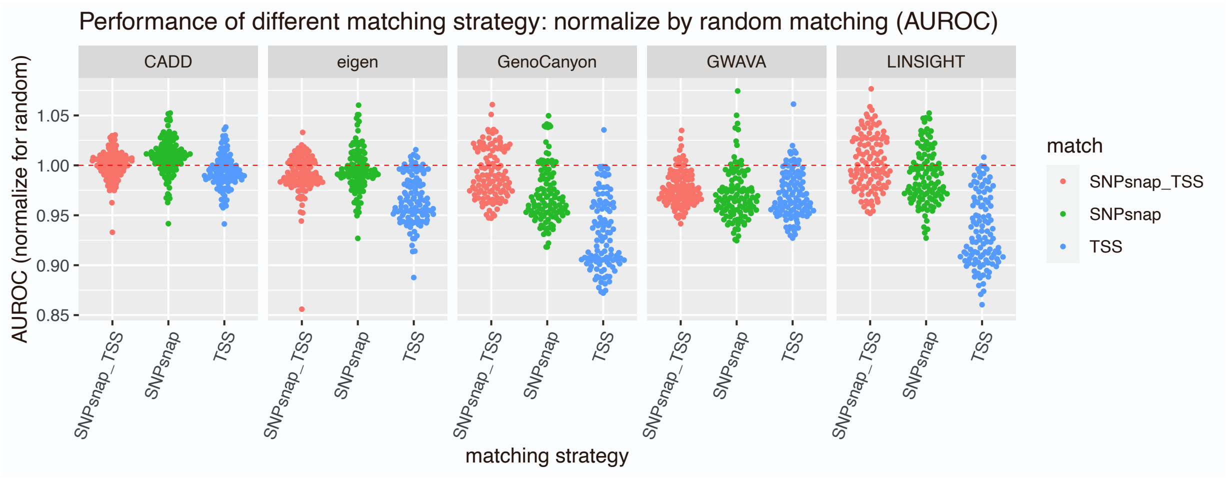

Supplemental Figure S2: ***Performance of different matching strategy, measured by area under the ROC curve***. X-axis delineates three different matching strategies (i.e. snpsnap-tss, snpsnap, tss). Y axis shows the performance in terms of area under receiver operating characteristic curve, normalized by random matching. Each point represents a specific disease term. Horizontal lines spanning the dataset denotes the scenario that the normalized performance equals to 1.

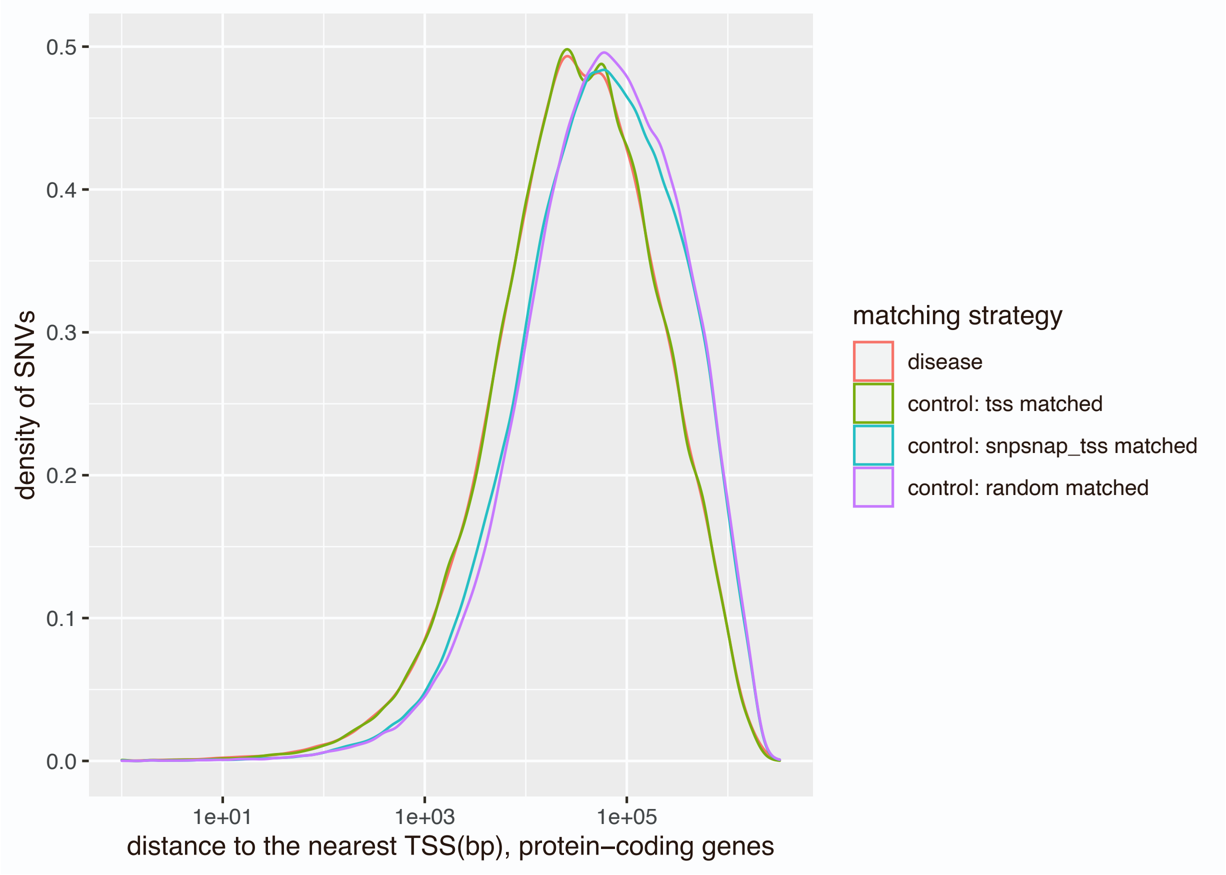

Supplemental Figure S3: ***A density plot showing the distribution of distance to nearest TSS (protein coding genes) in disease SNVs and three different control SNVs***. X-axis shows the distance to the nearest TSS of the protein-coding genes and is log 10 scaled. Y axis shows the density of SNVs.

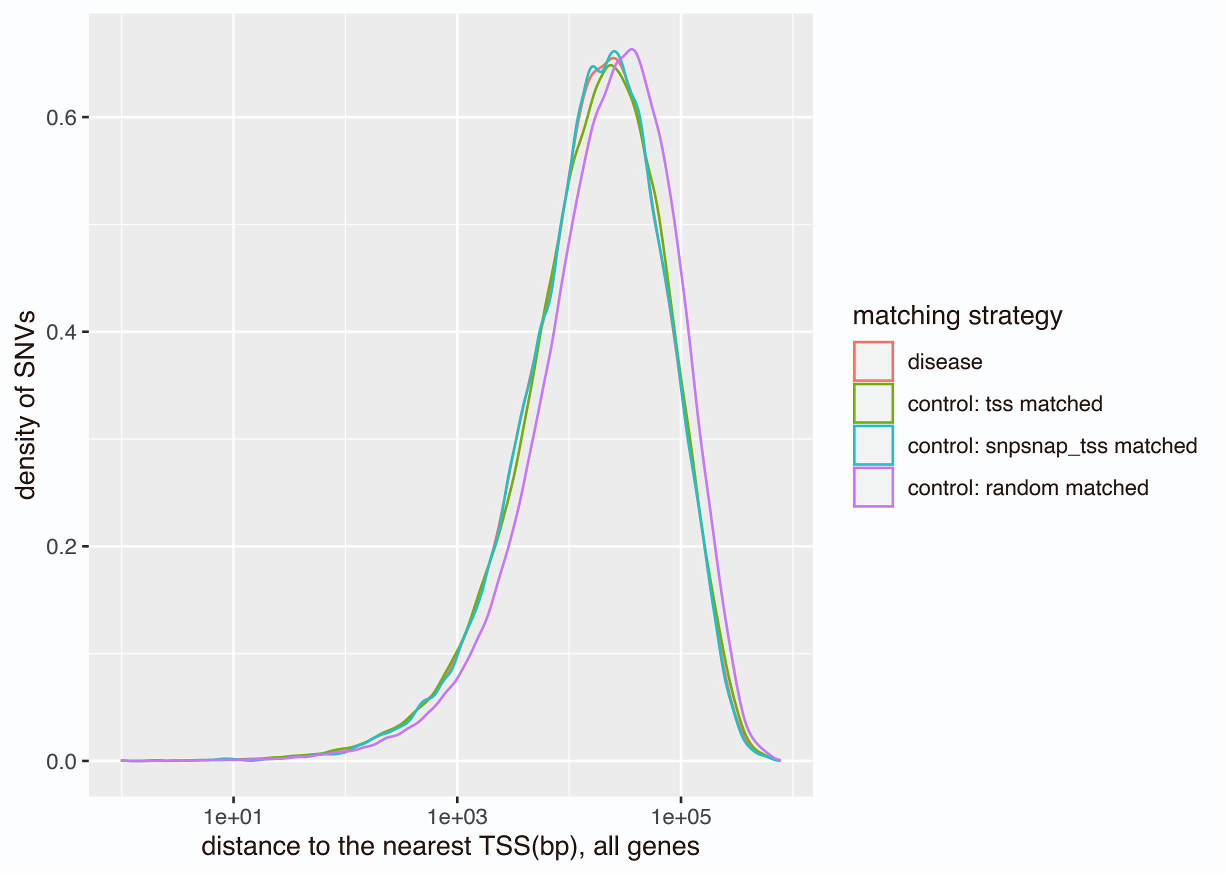

Supplemental Figure S4: ***A density plot showing the distribution of distance to nearest TSS (all genes) in disease SNVs and three different control SNVs***. X-axis shows the distance to the nearest TSS of the protein-coding genes and is log 10 scaled. Y axis shows the density of SNVs.

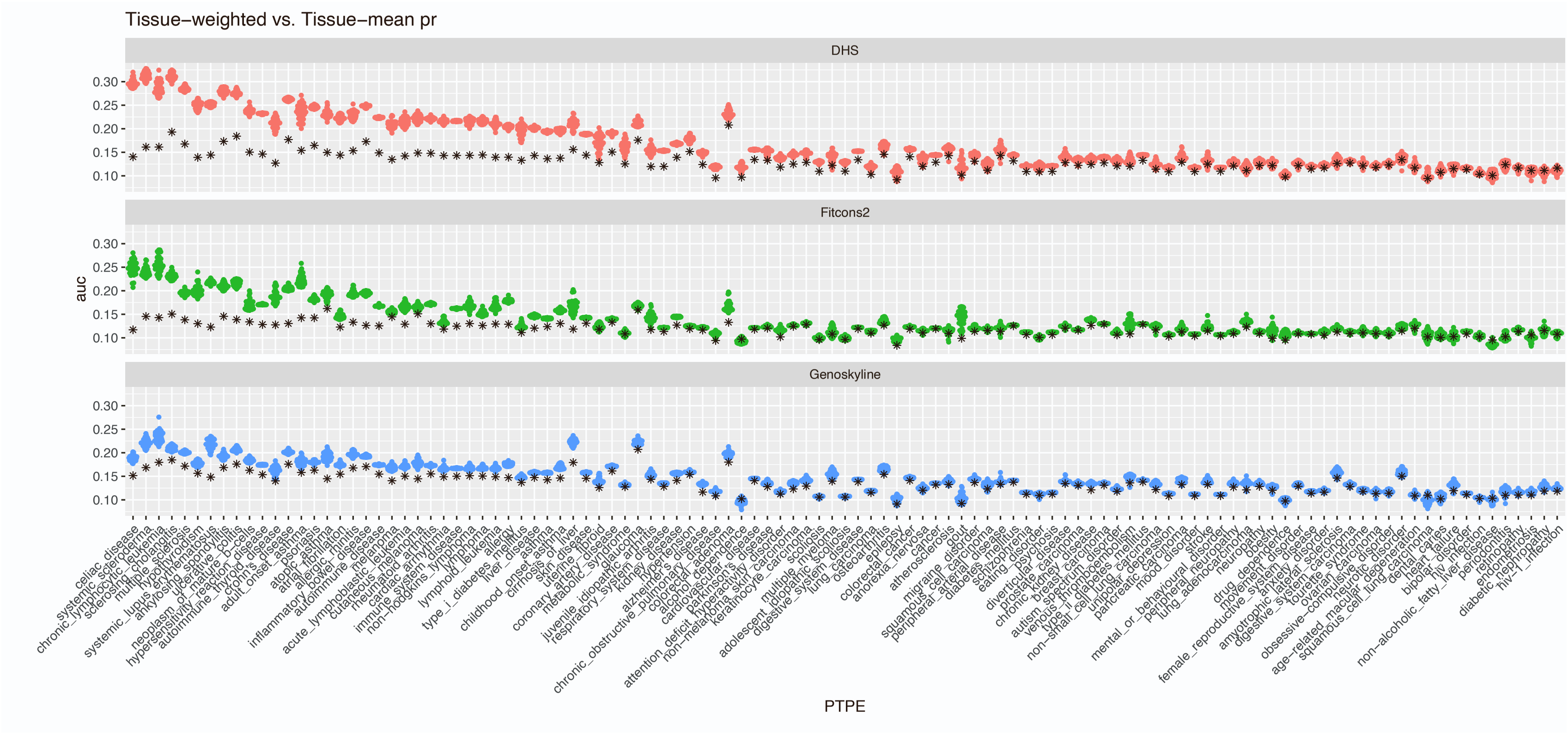

Supplemental Figure S5: ***Tissue-Weighted performance compared with Tissue-Mean in 111 diseases***. X-axis delineates different diseases and y-axis is the performance in terms of area under the precision recall curve. The star represents the Tissue-Mean and the colored dots are Tissue-Weighted with 30 replicates.

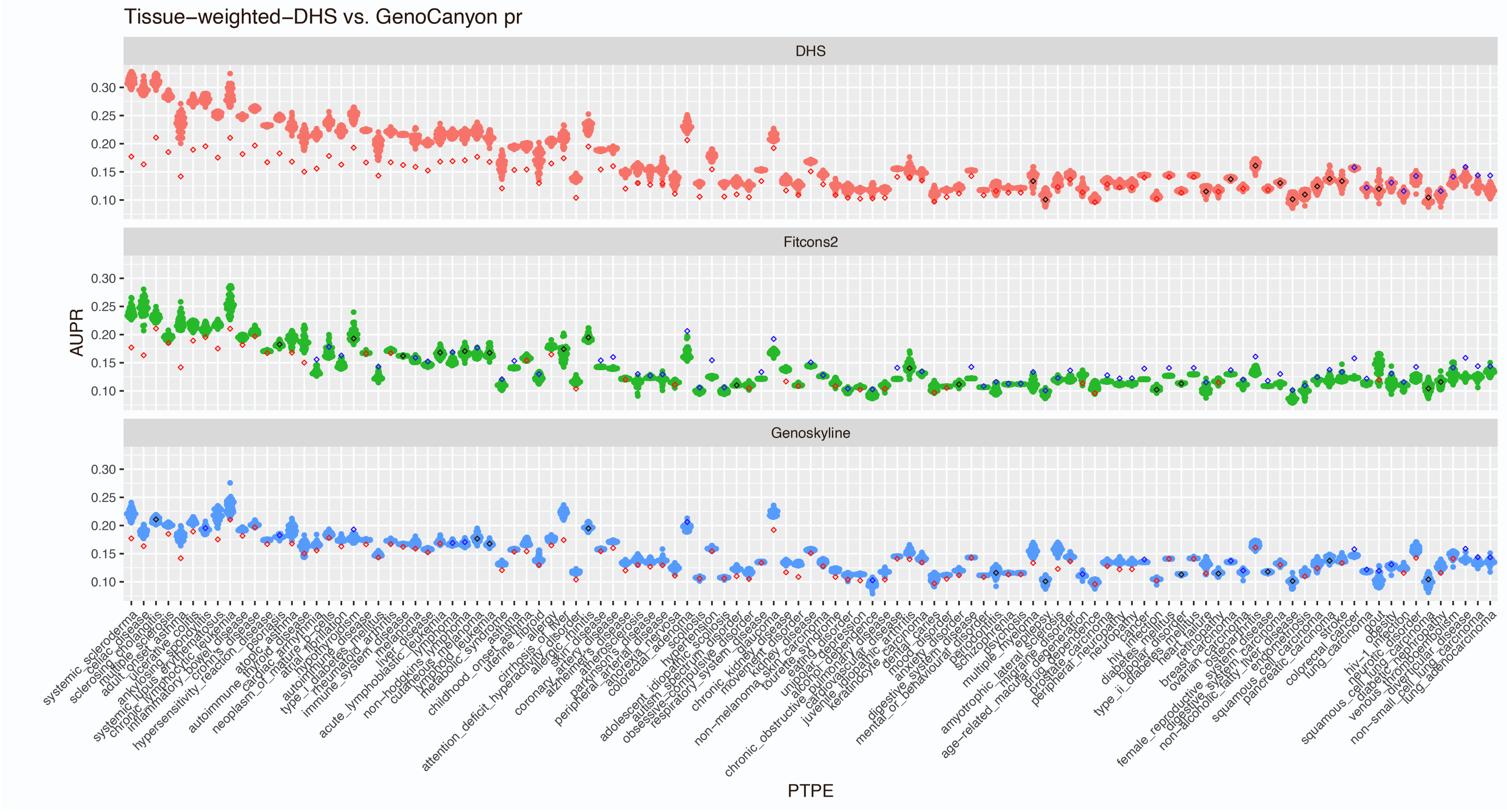

Supplemental Figure S6: ***Tissue-Weighted-DHS performance compared with GenoCanyon in 111 diseases***. X-axis delineates different diseases and y-axis is the performance in terms of area under the precision recall curve. The diamond represents the GenoCanyon (red: Tissue-Weighted better; black: comparable performance; blue: GenoCanyon better) and the colored dots are Tissue-Weighted with 30 replicates.

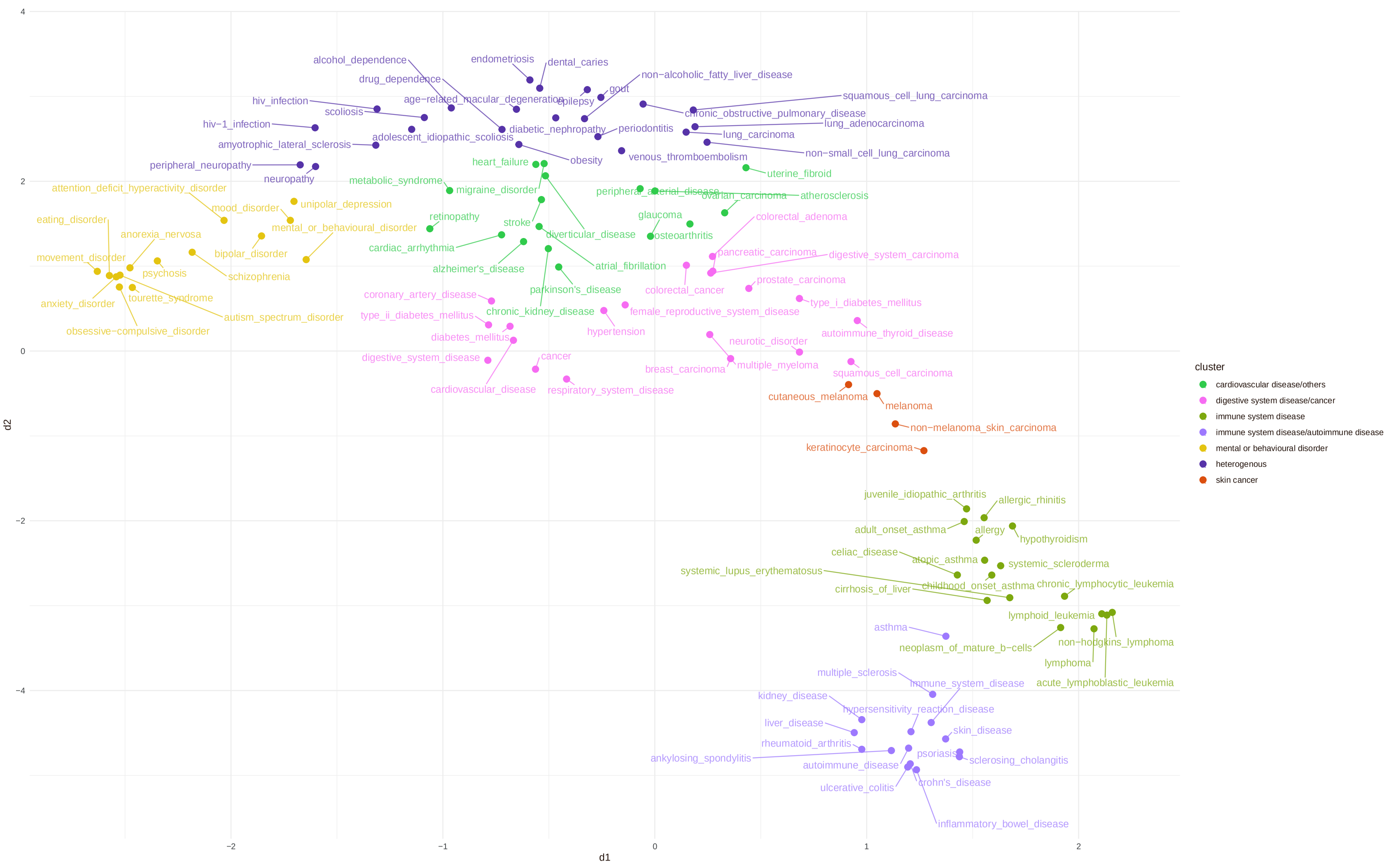

Supplemental Figure S7: ***Umap plot shows 7 clusters of 111 diseases***. Hierarchical clustering was used to group diseases into 7 clusters.

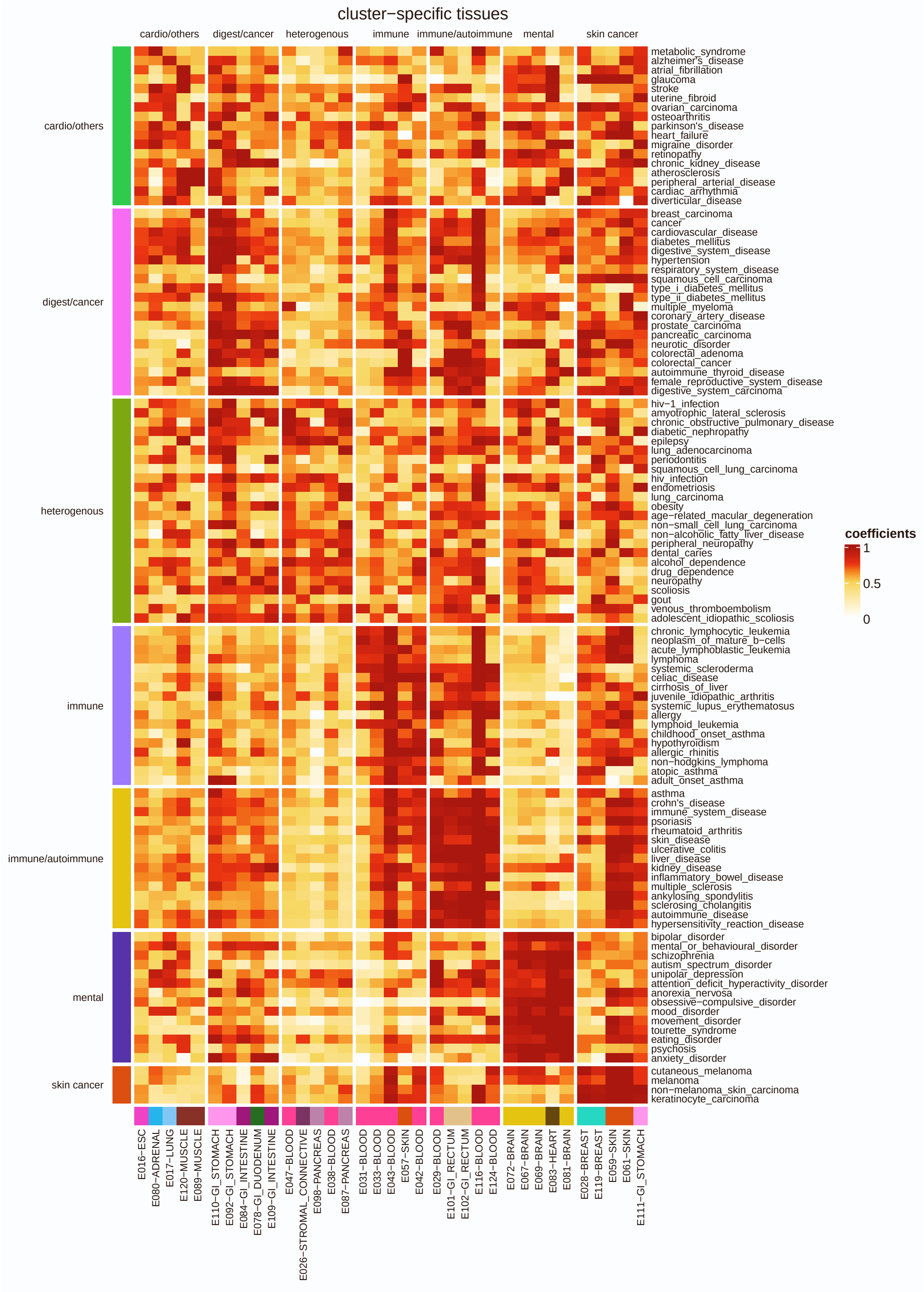

Supplemental Figure S8: ***Heatmap plot of coefficients of 111 diseases*** . Coefficients are regularized by each disease.

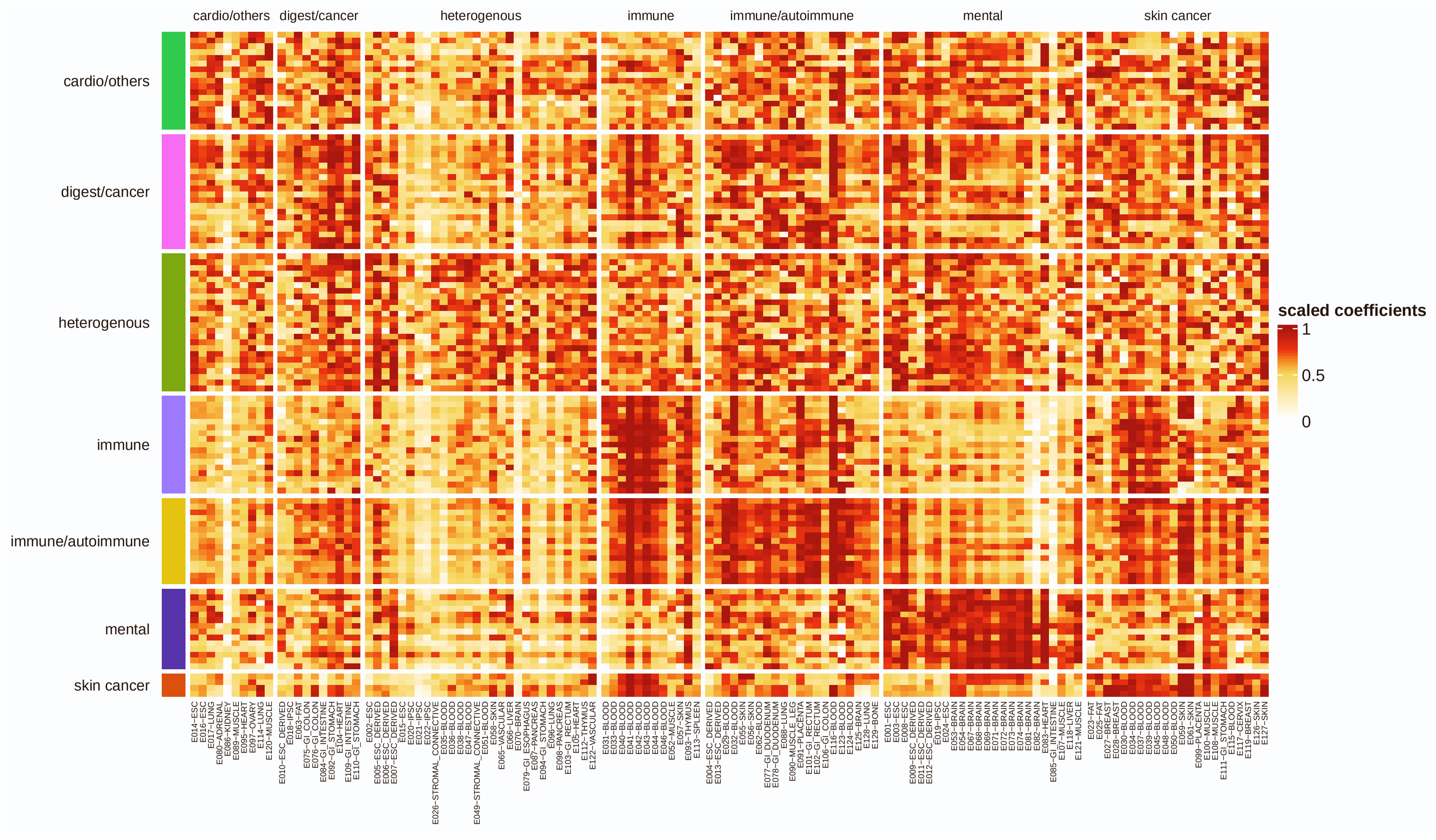

Supplemental Figure S9: ***Heatmap plot of coefficients of 111 diseases on 5 cluster-specific tissues***. Coefficients are regularized by each disease. Tissue names are shown by 'Tissue name-group' from 127 standard epigenomes.

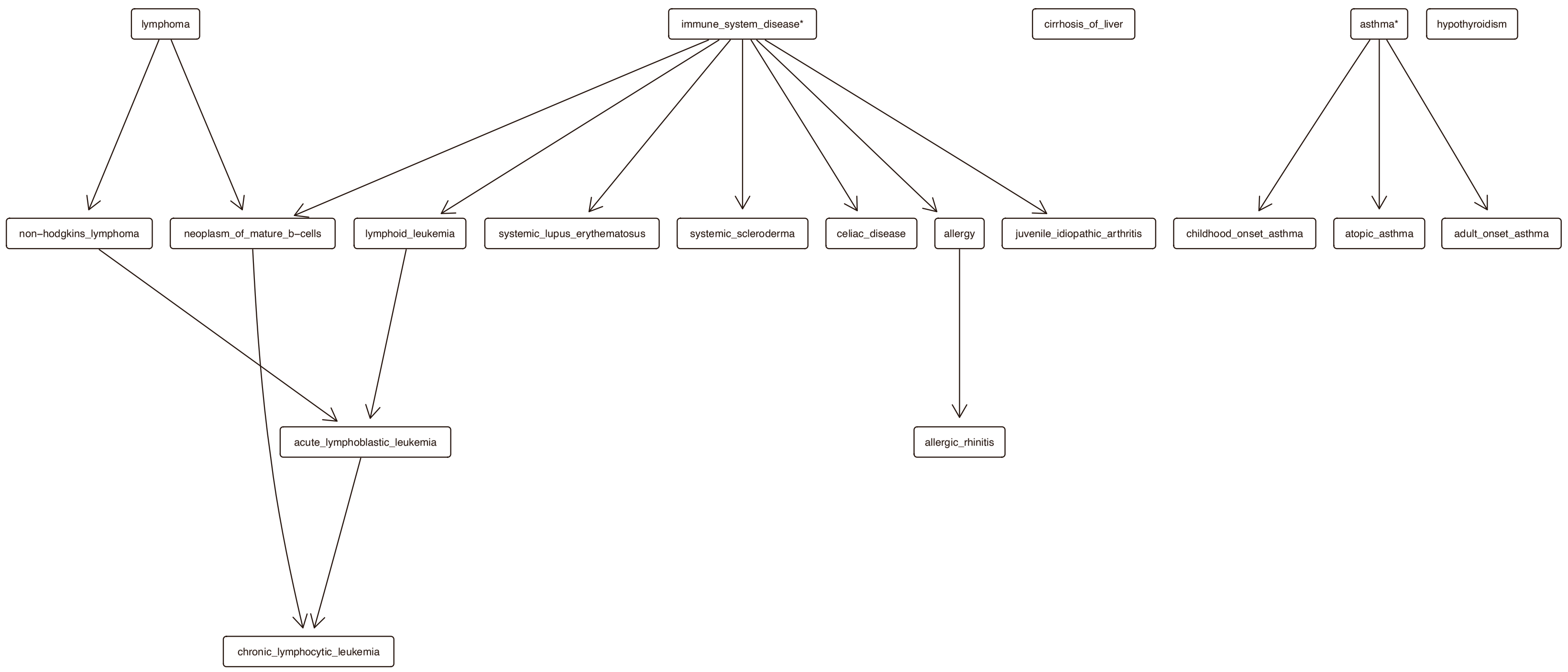

Supplemental Figure S10: ***Disease relationships for immune1 cluster***. The diseases placed at the top are more general than the diseases at the bottom. Arrow points from a more general term to a more specific term. A disease marked with one star indicates that it is not in this cluster but among the 111 diseases we studied. Diseases with two stars indicate that they are not among the 111 diseases.

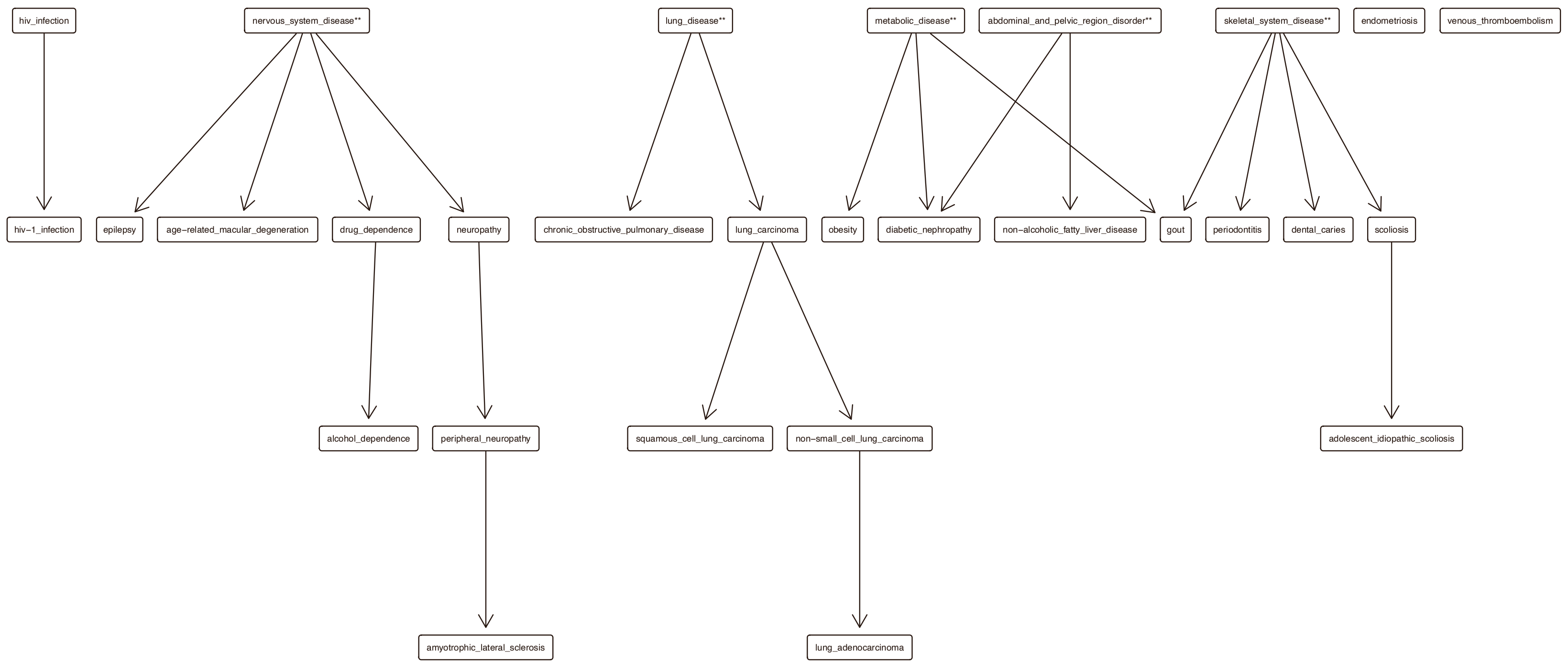

Supplemental Figure S11: ***Disease relationships for others cluster***. The diseases placed at the top are more general than the diseases at the bottom. Arrow points from a more general term to a more specific term. A disease marked with one star indicates that it is not in this cluster but among the 111 diseases we studied. Diseases with two stars indicate that they are not among the 111 diseases.

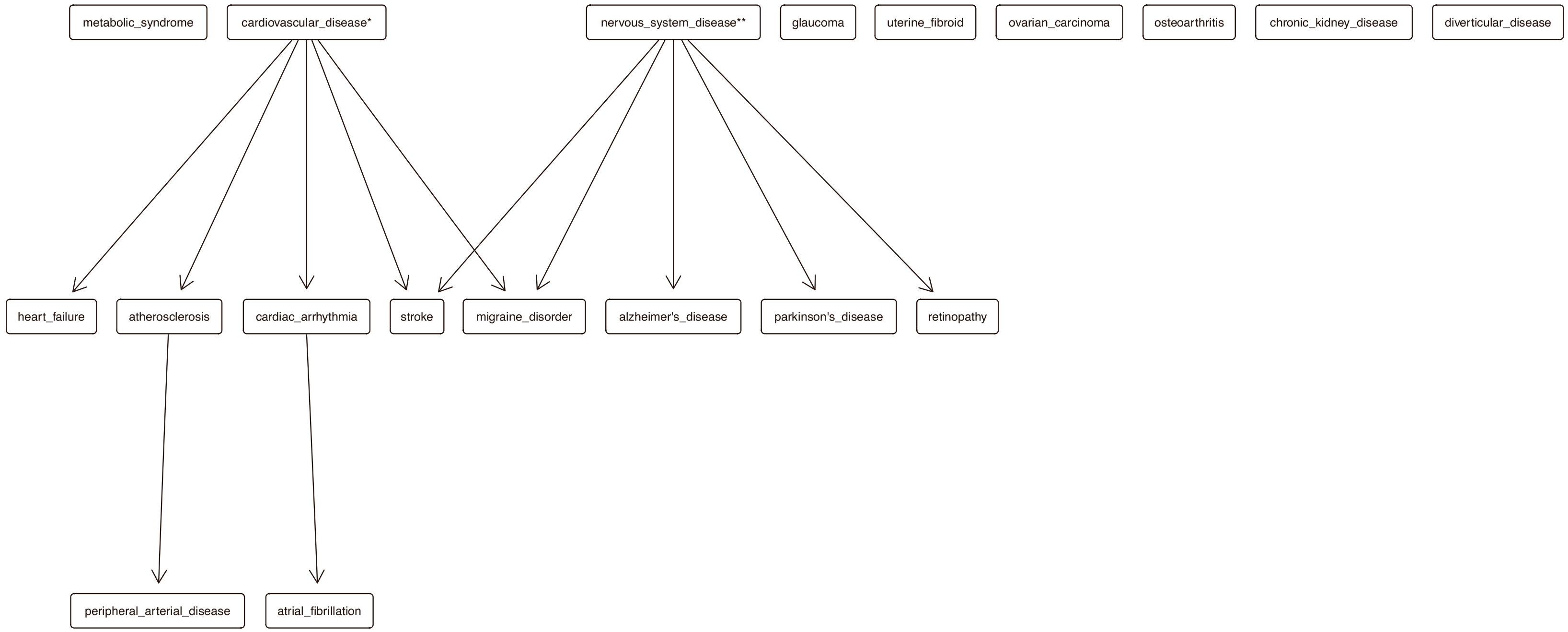

Supplemental Figure S12: ***Disease relationships for cardiovasular disease and others cluster***. The diseases placed at the top are more general than the diseases at the bottom. Arrow points from a more general term to a more specific term. A disease marked with one star indicates that it is not in this cluster but among the 111 diseases we studied. Diseases with two stars indicate that they are not among the 111 diseases.

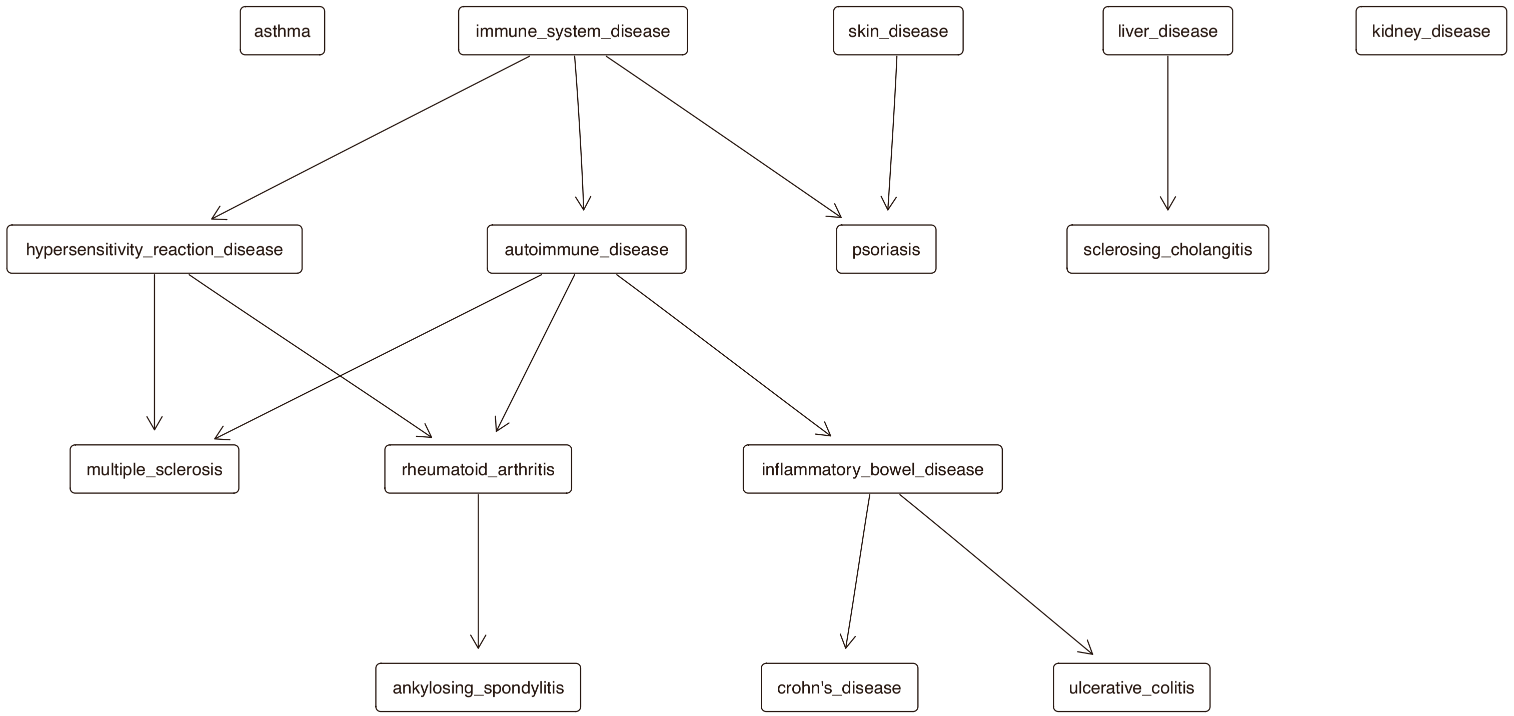

Supplemental Figure S13: ***Disease relationships for immune2 cluster***. The diseases placed at the top are more general than the diseases at the bottom. Arrow points from a more general term to a more specific term. A disease marked with one star indicates that it is not in this cluster but among the 111 diseases we studied. Diseases with two stars indicate that they are not among the 111 diseases.

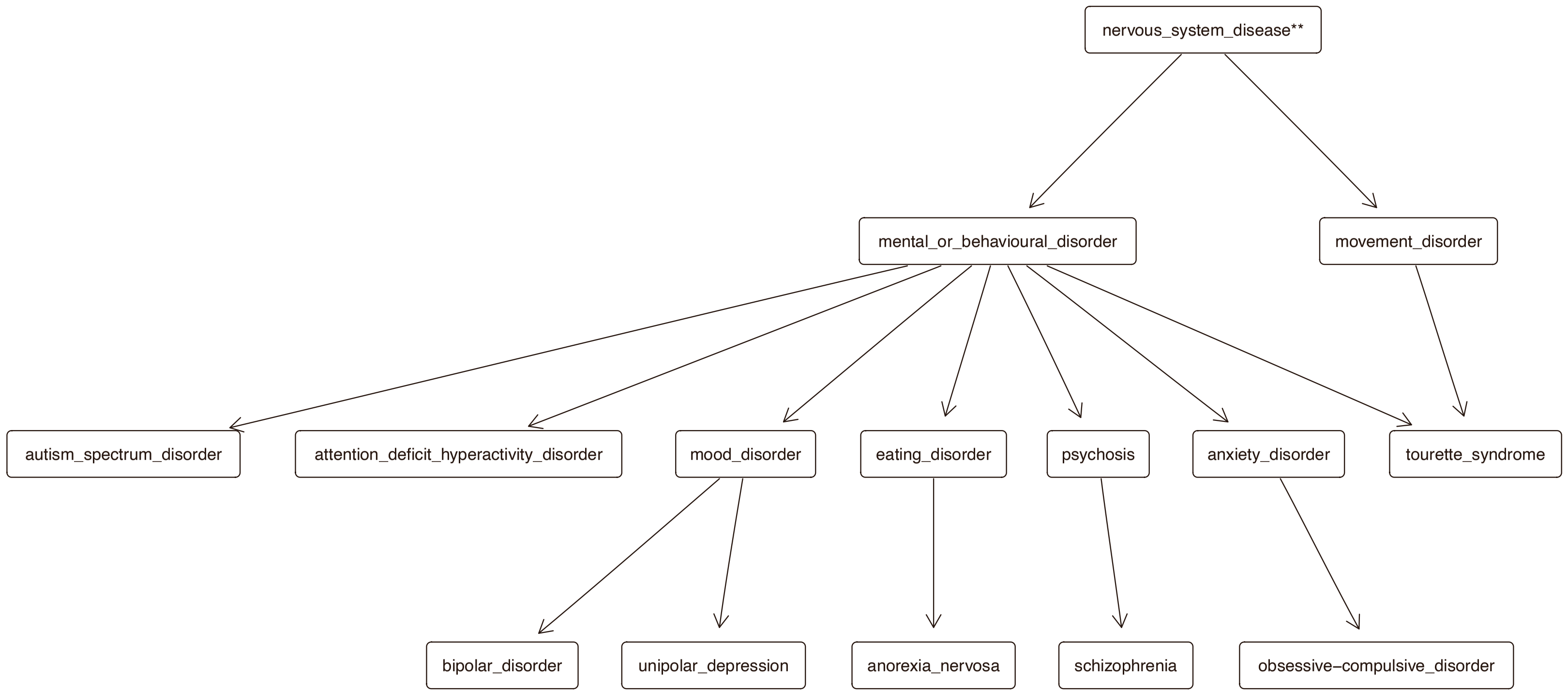

Supplemental Figure S14: ***Disease relationships for mental or behavioural disorder cluster***. The diseases placed at the top are more general than the diseases at the bottom. Arrow points from a more general term to a more specific term. A disease marked with one star indicates that it is not in this cluster but among the 111 diseases we studied. Diseases with two stars indicate that they are not among the 111 diseases.

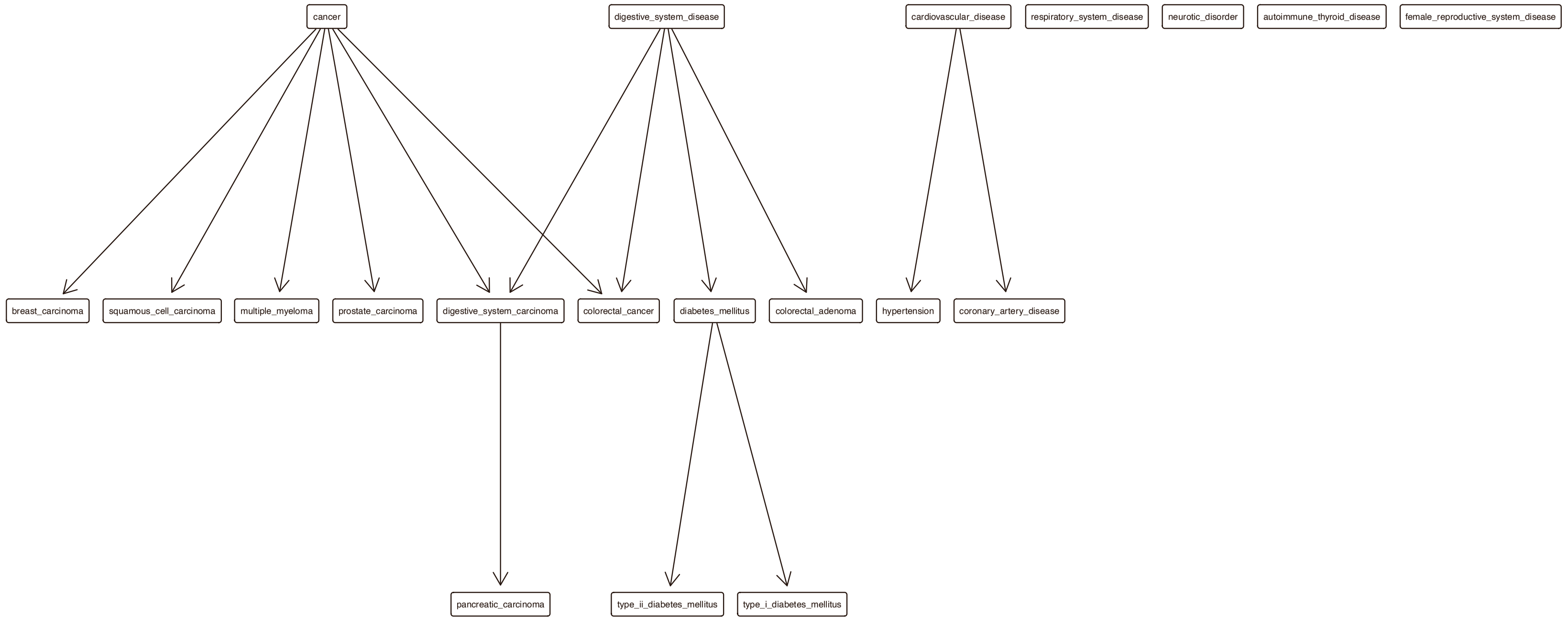

Supplemental Figure S15: ***Disease relationships for digestive and cancer cluster***. The diseases placed at the top are more general than the diseases at the bottom. Arrow points from a more general term to a more specific term. A disease marked with one star indicates that it is not in this cluster but among the 111 diseases we studied. Diseases with two stars indicate that they are not among the 111 diseases.

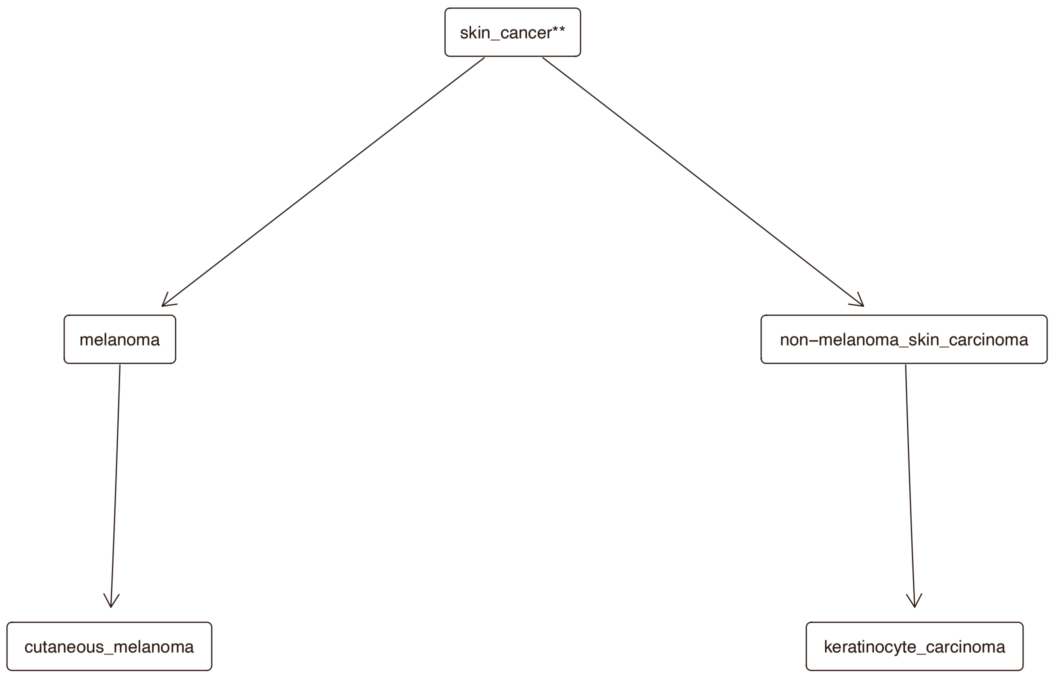

Supplemental Figure S16: ***Disease relationships for skin cancer cluster***. The diseases placed at the top are more general than the diseases at the bottom. Arrow points from a more general term to a more specific term. A disease marked with one star indicates that it is not in this cluster but among the 111 diseases we studied. Diseases with two stars indicate that they are not among the 111 diseases.

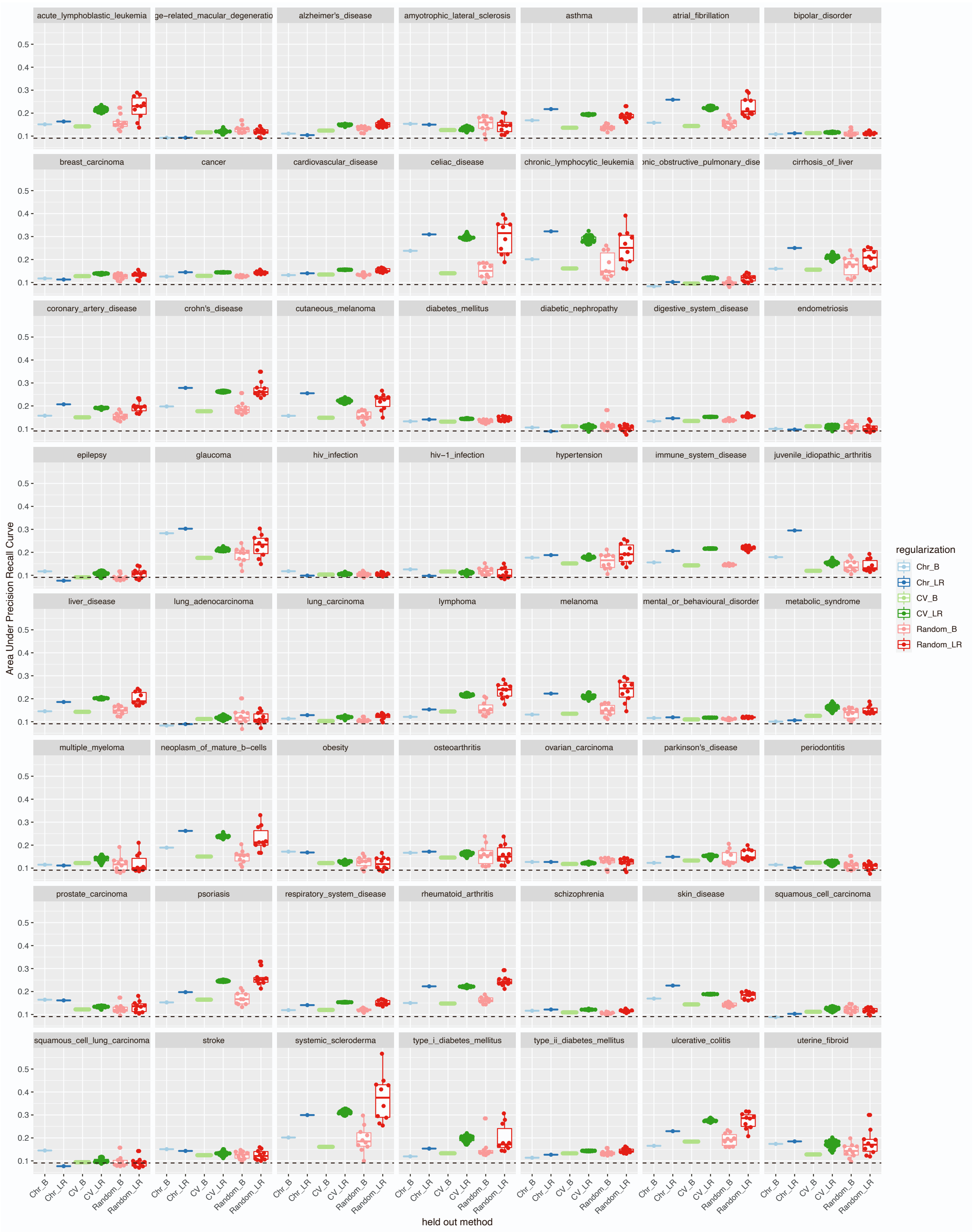

Supplemental Figure S17: ***Performance of Tissue-weighted (DHS) in different held-out strategies***. Chr-B: test set held out by chromosome, baseline; Chr-LR: test set held out by chromosome, logistic regression; CV-B: cross-validation, baseline; CV-LR: cross-validation logistic regression; random-B: randomly sampled test set, baseline; random-LR: randomly sample test set, logistic regression.

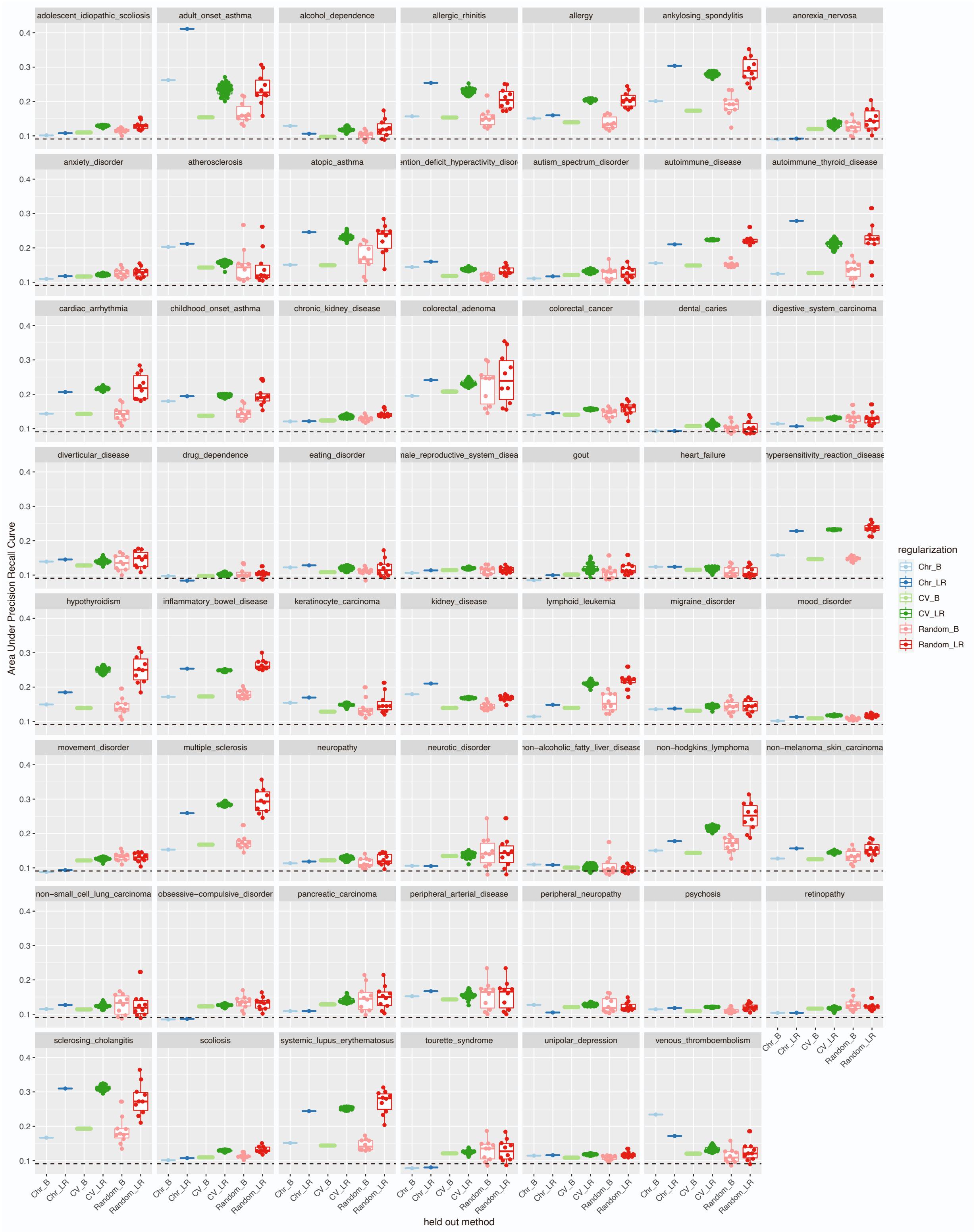

Supplemental Figure S18: ***Performance of Tissue-weighted (DHS) in different held-out strategies, continued***. Chr-B: test set held out by chromosome, baseline; Chr-LR: test set held out by chromosome, logistic regression; CV-B: cross-validation, baseline; CV-LR: cross-validation logistic regression; random-B: randomly sampled test set, baseline; random-LR: randomly sample test set, logistic regression.

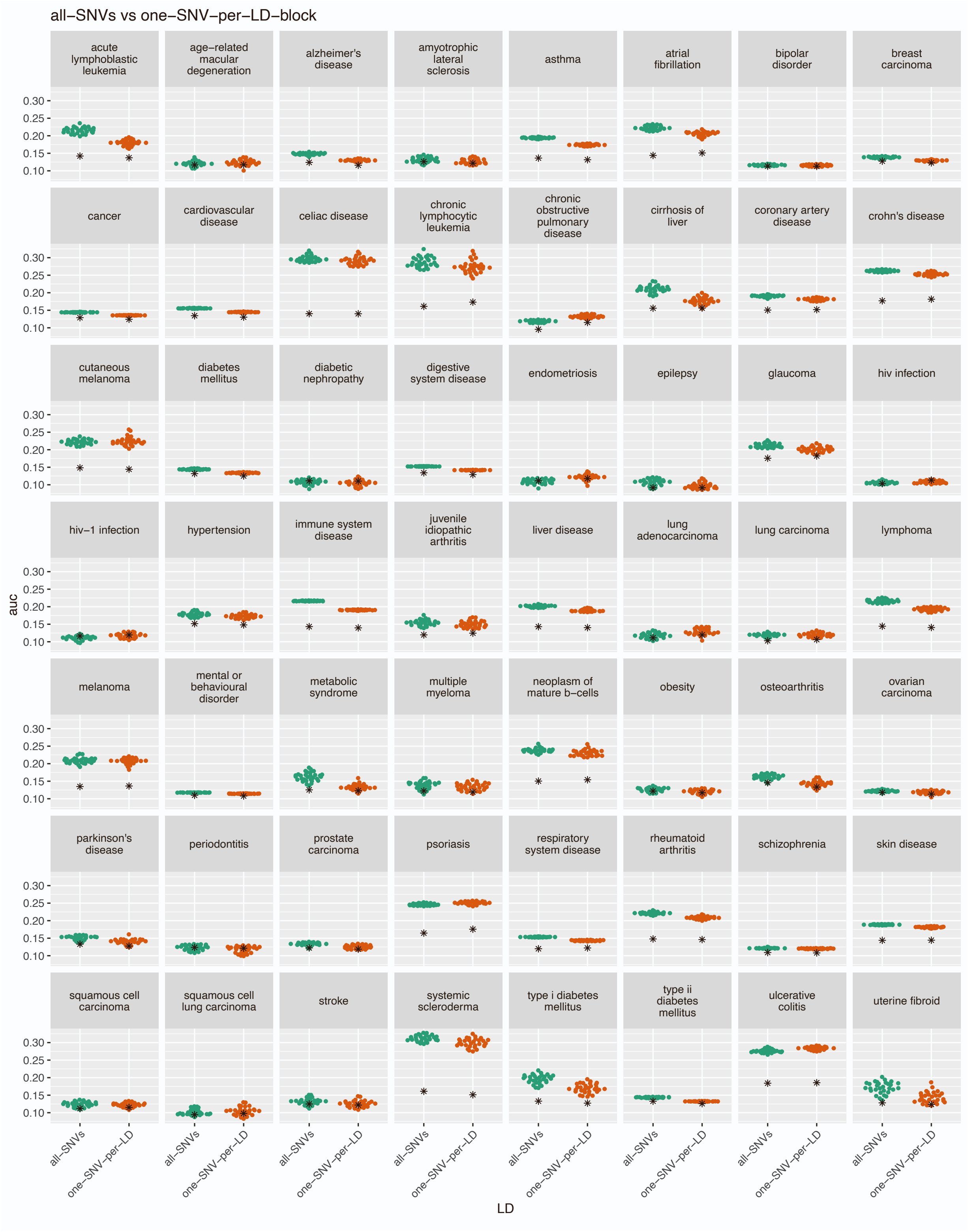

Supplemental Figure S19: ***Performance of Tissue-weighted (DHS) in all SNVs or representative SNVs (one SNV per LD block)***. Colored dots represent the performance of tissue-weighted (DHS) in all SNVs or representative SNVs. Stars represent the baseline performance (tissue-mean DHS) in all SNVs or representative SNVs.

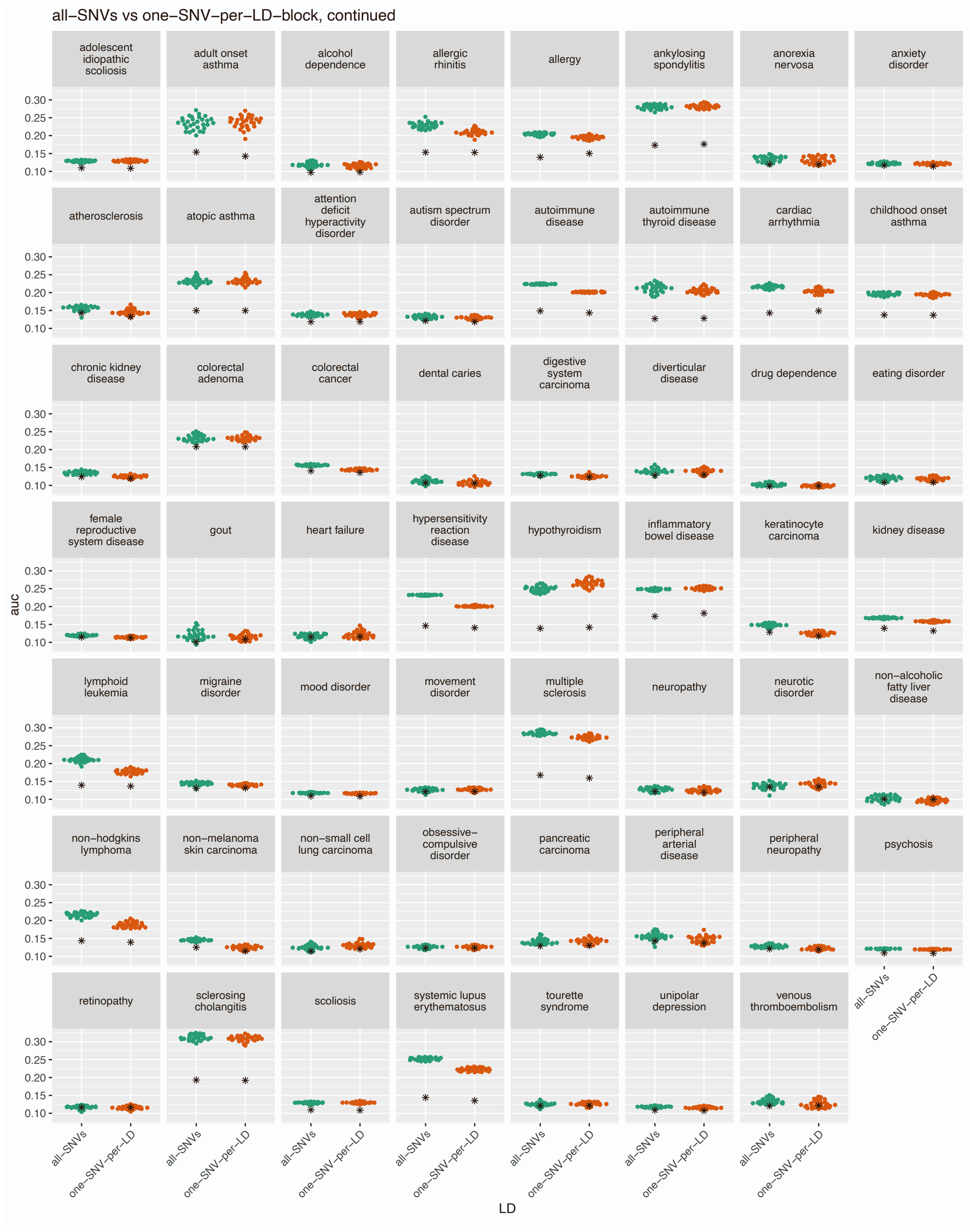

Supplemental Figure S20: ***Performance of Tissue-weighted (DHS) in all SNVs or representative SNVs (one SNV per LD block), continued***. Colored dots represent the performance of tissue-weighted (DHS) in all SNVs or representative SNVs. Stars represent the baseline performance (tissue-mean DHS) in all SNVs or representative SNVs.
